# Digital Design of Filtration and Washing of Active Pharmaceutical Ingredients via Mechanistic Modeling

**DOI:** 10.1021/acs.oprd.2c00165

**Published:** 2022-12-06

**Authors:** Sara Ottoboni, Cameron J. Brown, Bhavik Mehta, Guillermo Jimeno, Niall A. Mitchell, Jan Sefcik, Chris J. Price

**Affiliations:** † EPSRC Future Manufacturing Hub in Continuous Manufacturing and Advanced Crystallisation, University of Strathclyde, GlasgowG1 1RD, U.K.; ‡ Department of Chemical and Process Engineering, University of Strathclyde, GlasgowG1 1XJ, U.K.; § Strathclyde Institute of Pharmacy & Biomedical Science (SIPBS), University of Strathclyde, GlasgowG4 0RE, U.K.; ∥ Siemens Process Systems Engineering Ltd., LondonW6 7HA, U.K.

**Keywords:** filtration, washing, modeling, gPROMS, model comparison

## Abstract

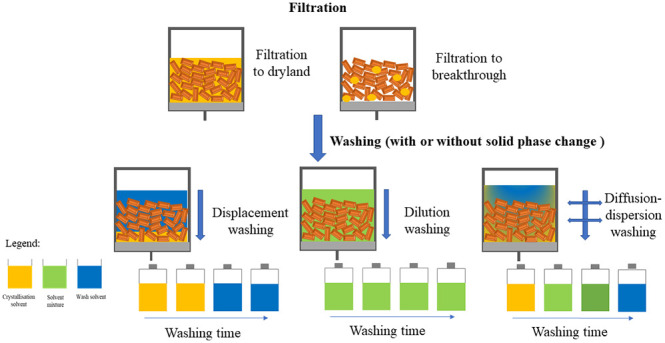

To facilitate integrated end-to-end pharmaceutical manufacturing using digital design, a model capable of transferring material property information between operations to predict product attributes in integrated purification processes has been developed. The focus of the work reported here combines filtration and washing operations used in active pharmaceutical ingredient (API) purification and isolation to predict isolation performance without the need of extensive experimental work. A fixed Carman–Kozeny filtration model is integrated with several washing mechanisms (displacement, dilution, and axial dispersion). Two limiting cases are considered: case 1 where there is no change in the solid phase during isolation (no particle dissolution and/or growth), and case 2 where the liquid and solid phases are equilibrated over the course of isolation. In reality, all actual manufacturing conditions would be bracketed by these two limiting cases, so consideration of these two scenarios provides rigorous theoretical bounds for assessing isolation performance. This modeling approach aims to facilitate the selection of most appropriate models suitable for different isolation scenarios, without the requirement to use overly complex models for straightforward isolation processes. Mefenamic acid and paracetamol were selected as representative model compounds to assess a range of isolation scenarios. In each case, the objective of the models was to identify the purity of the product reached with a fixed wash ratio and minimize the changes to the crystalline particle attributes that occur during the isolation process. This was undertaken with the aim of identifying suitable criteria for the selection of appropriate filtration and washing models corresponding to relevant processing conditions, and ultimately developing guidelines for the digital design of filtration and washing processes.

## 1 Introduction

The pharmaceutical industry is beginning to adopt continuous active pharmaceutical ingredient (API) manufacturing to reduce production costs, improve manufacturing flexibility, reduce infrastructure costs, reduce manufacturing lead time (typically 6 months to 10 days), and improve sustainability.^1,2^ A further driver is the reduction of variance in API quality critical attributes.^3,4^ To facilitate the transition from batch to continuous manufacturing, it is necessary to “smartly” integrate single continuous unit operations to achieve a continuous material flow from synthesis through to formulation.^5^ To achieve this smart integration of unit operations, a combination of modeling, online measurement, and advanced control techniques is vital to predict product property outcomes, to design and control processes, and to reduce the risk of nonconforming products.^2,6^ Another challenge facing the pharmaceutical industry is to reduce the amount of material consumed during process development.^7,8^ An ambitious goal is to consume just 100 g of API (and the corresponding precursors) and complete development in 100 days.^9^ Digital design of continuous API manufacturing offers a path to achieving this goal. This includes modeling and predicting process performance as a function of the operating conditions for both individual continuous unit operations and for the integrated processes with the aim of optimizing process design and reducing the laboratory time and cost needed to develop new products. While a few examples of modeling integrated continuous unit operations using flowsheet models^10−16^ have been published, these are mainly focused on secondary drug product manufacture rather than API synthesis, crystallization, and isolation.^17^

Classical isolation models do not consider integrated filtration and washing processes, but filtration and washing are seen as two separate process operations, with different models used to model these two processes. Dead-end filtration can be studied using different models, but the common model used is the conventional cake filtration theory.^18^ Conventional filtration theory describes the relevant continuity equations, the closing relationship, and the appropriate initial boundary conditions and moving boundary conditions^19,20^ of a filtration process. A further detailed description of the existing filtration models was reviewed by Ottoboni et al.^21^ Nagy et al.,^22^ Other filtration models were reported by Benyahia et al.^23^ and Destro et al.^24,25^ In general, these models are based on Darcy’s law, but different theories are used to calculate cake porosity and cake resistance, such as the Yu et al.^26^ approaches proposed by Destro et al.^24^ or the Endo Alonso theory proposed by Nagy et al.^22^

One of the first washing models developed was proposed by Rhodes,^27,28^ used to describe the variables affecting the washing curve. This is further described in Section 3.1. Different behaviors are observed according to the nature of the mother liquor and the washing solvent.^29^ In general, it appears that when the mother liquor has a strong wetting preference for the solid, the nonwetting fluid (wash solvent (WS)) tends to occupy the largest pores, and the wetting fluid the finer ones. Thus, there may be two separate networks, each of which contains its own fluid phase. This behavior was described by the main and side channel models.^28,30,31^ Other researchers have tried to model the washing process,^30,32,33^ considering only the diffusion–dispersion washing mechanism, without including the risk of solid phase dissolution. Another approach to predict the washing curve considering a washing process driven by displacement, diffusion, and dilution washing is reported by Svarovsky^33^ and Wakeman and Attwook.^34^ Järveläinen and Nordén,^35^ Backhurst et al.,^36^ Arora et al.,^37^ and Destro et al.^24^ discussed the effect of Peclet number and diffusivity coefficient on the shape of the wash curve. Benyahia et al.^23^ reported a washing model based on a simple mass balance.

In 2009, Ruslim et al.^38^ tried to modify the classical washing model to study cases where the API is soluble in mother liquor and wash solvent because product loss during washing is an important parameter to consider. However, this work mainly considers the variation of the wash curve, without considering the possibility of using the model as a tool to predict the particle size variation caused by agglomeration, dissolution, and deposition. So far only empirical approaches have been studied to investigate the role of solvents, particle characteristics, and process conditions^39−42^ in agglomeration, dissolution of the solid phase during washing,^43^ and deposition during washing.^44^ The approach proposed in this work considers isolation as an integrated unit operation, where filtration and washing are modeled using the input slurry composition generated during the crystallization upstream process. No experimental data have been used for this, and instead hypothetical cases have been used to show the effect of the different filtration and washing models. In future activity, impurity removal work will be considered, including experimental data collection for validating the various filtration and washing models. In this work, filtration is modeled considering two different filtration-stopping procedures: stopping filtration at dryland or continuing to breakthrough. Halting the filtration at dryland ensures that the cake remains fully saturated with impure mother liquor that occupies all of the interparticulate pores. The stopping condition for breakthrough is typically when air or nitrogen from above the cake forms bubbles with the mother liquor emerging on the low-pressure side of the medium supporting the cake. In this case, more of the impure mother liquor is removed. Deliquoring is not considered in this paper. The difference between dryland and breakthrough is estimated by the free liquid height and the cake mass fraction, respectively. The method here proposed does not account for multiphase flow, capillarity, surface tension, and porosity effects that have been previously included to model filtration to breakthrough in more comprehensive deliquoring models.^24,45^ Two limiting cases are considered: case 1 where there is no change in the solid phase during isolation (no particle dissolution and growth), and case 2 where the liquid and solid phases are equilibrated over the course of isolation. In reality, all actual manufacturing conditions would be bracketed by these two limiting cases, so consideration of these two scenarios provides rigorous theoretical bounds for assessing isolation performance. Different isolation scenarios were demonstrated through computational investigation of a series of factors (e.g., crystallization and wash solvent, isolation driving force, and volume and number of washes used) to identify their effect on filtration and washing responses for two model compounds, paracetamol (PCM) and mefenamic acid (MA).

## 2 Materials

The model is based on the isolation of paracetamol and mefenamic acid, based on the particle properties of paracetamol supplied by Mallinckrodt, Inc. (typically crystalline), and mefenamic acid supplied by Sigma-Aldrich. The solvents used were isopropanol, *n*-heptane, acetonitrile, *n*-dodecane, water, and 2-butanol. The solvent properties were collected from the literature.

## 3 Model Methodology

**Figure 1:**
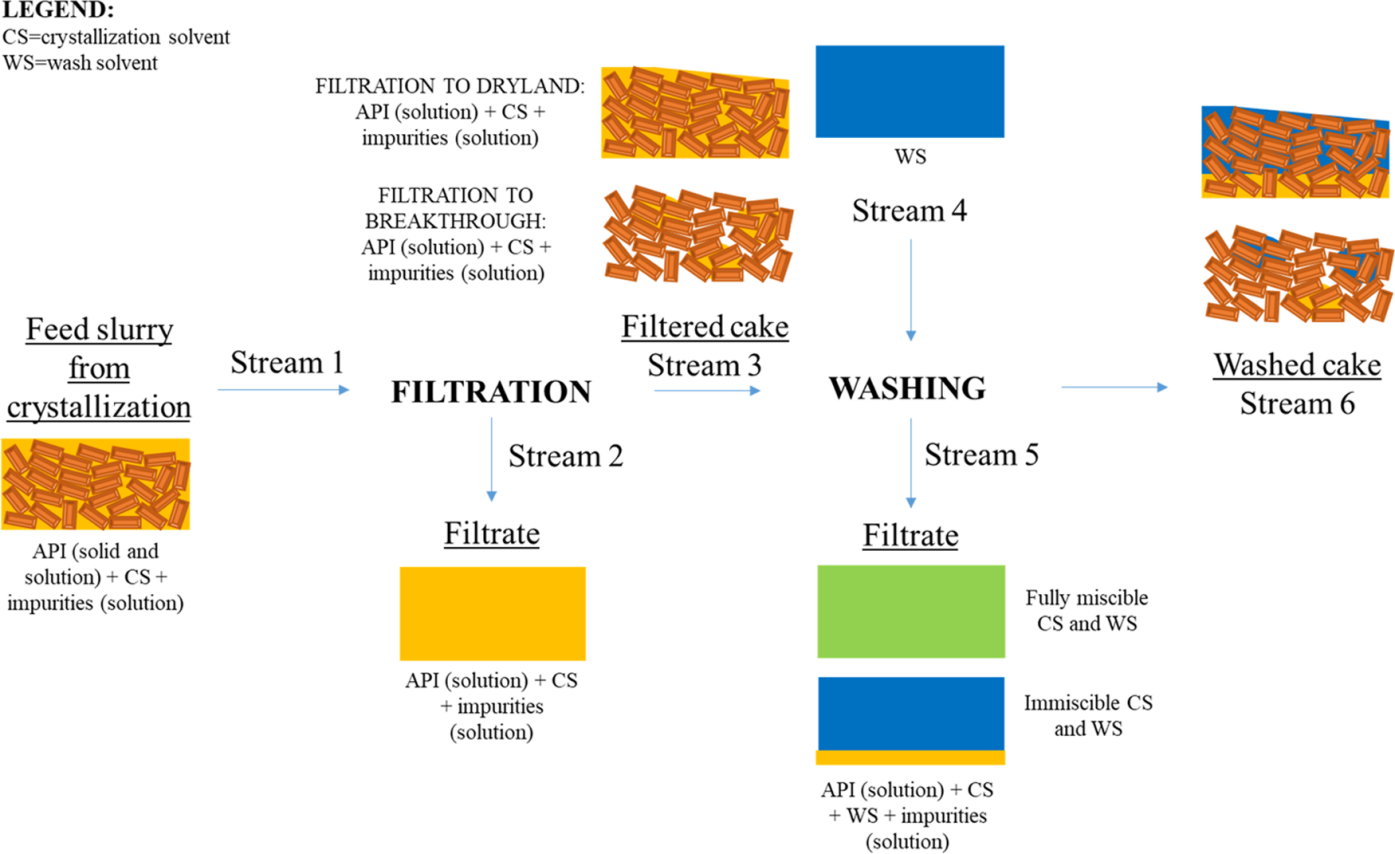
Schematic flow diagram of the input and output streams of an isolation process.

Isolation comprises three different subprocesses: filtration, washing, and drying. A schematic representation is shown in Figure 1.

Stream 1 is the input stream for isolation and corresponds to the outlet stream of crystallization. During filtration, the suspension is separated into two phases, the cake and the filtrate, by generating a pressure differential across the filter chamber.^41,42^ Stream 2 corresponds to the filtrate removed during filtration, while stream 3 corresponds to the cake part of the filtered suspension. The cake consists of the solid part of the suspension (crystallized active ingredient) and a small fraction of the mother liquor; the amount of the residual mother liquor fraction left in the cake can vary according to the filtration end point used (dryland or breakthrough). The filter cake and residual mother liquor (stream 3) and wash solvent (stream 4) system is then split into two fractions by again generating a pressure differential across the filter chamber: stream 5 (filtrate) and stream 6 (washed cake). During washing, a series of mechanisms act simultaneously to remove the residual mother liquor and to remove impurities deposited on the crystal surfaces: displacement, dilution, diffusion, and dispersion.^31,46−51^

The washing curve provides a graphical representation of the variation of species concentration during washing, it has on the *y*-axis the dimensionless solute concentration of the wash filtrate, this is plotted against the wash ratio.^31,51^ The initial phase of the washing curve is a result of a direct hydrodynamic displacement of the residual liquor from the larger pores due to the wash liquid entering the cake. The second intermediate stage occurs when there is direct displacement from the smaller flow pores in the cake; during this stage, the wash solvent starts to dilute the filtrate from the larger pores in which a mass transfer process has started. In the third regime, the mass transfer stage, the solute diffuses into the wash solvent; this takes place over the entire void volume of the cake. The relative importance of each of these stages depends on the physical operating conditions, the microstructures of the flow, the pore network of the filter cake, and the properties of the mother liquor and the wash solvent.

In this work, we propose a series of models considering the different washing mechanisms and interaction with the solid cake, through dissolution or growth, as follows:

Case 1—Assumes that no changes in solid phase are considered (no particle dissolution or growth). Thus, case 1 can describe three different washing mechanisms:

(a) Pure displacement.
(b) Dilution with perfect liquid mixing.
(c) Diffusion with axial dispersion.

Case 2—Allows for the possibility of cake and impurity dissolution during washing. This case presumes that kinetic aspects can be neglected and solid–liquid equilibrium is reached instantaneously. As in case 1, three different washing mechanisms are considered:

(a) Displacement washing process where the mother liquor and the wash solvent are immiscible, and dissolution of the solid phase is caused by the non-null solubility of the compound in the wash solvent.
(b) Dilution and dissolution are considered for a system where instant liquid-phase mixing is assumed in the entire volume of the voids in the cake.
(c) Diffusion with axial dispersion and dissolution are considered. The system shows vertical heterogeneity and there is a composition gradient along the height of the cake.

### 3.1 Filtration: Equations, Assumptions, and Constrains

Dead-end filtration is the most common method of filtration and can be studied using different models. The simplest model is the conventional cake filtration theory.^30,52,53^

Cake porosity is the fraction of the bulk volume of the cake that is occupied by pore/void space and can be defined as
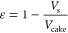
1In general, the specific resistance of the cake of a filter cake is defined as the resistance of fluid to pass through the cake; this parameter is inversely related to the permeability of the cake, *k*, and the equivalent diameter corresponding to the particle volume, *x*_sv_
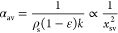
2According to the Carman–Kozeny equation,^54^ cake resistance is also related to cake porosity
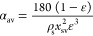
3Cake porosity is independent of particle size, but it is a function of particle size distribution (PSD), as explained above. Other approaches are commonly used to determine cake resistance in accordance with the particle size distribution (PSD) and the shape of particles.^46,55,56^

The approximations used in these models are reported in Table 1.

**Table 1:** Assumptions Used for the Filtration Model

assumption/approximation	description
cake resistance equation—particle size	the particle size used corresponds to a single particle size, the Sauter mean/volumetric mean diameter
cake resistance equation—particle shape	the model uses the Carman–Kozeny equation which does not consider particle aspect ratio as a parameter that affects cake resistance; the model is based on approximating particle shape as equivalent spheres; for future consideration, the model can be improved using other approaches (e.g., Endo Alonso)^52,54^ that consider shape and texture of particles can be represented by a fractal structure or aspect ratio distribution

The resistance of the cake can be then used to calculate the flow rate, along with media resistance of the medium and other filtration parameters using Darcy’s law for constant pressure^53^
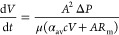
4Filtration was simulated using a gPROMS FormulatedProducts filtration model, where the Carman–Kozeny theory was used. The filtration process was modeled using the initial conditions reported in Table 2.

**Table 2:** Initial Filtration Conditions Selected for Paracetamol and Mefenamic Acid Case Study Simulations Performed with gPROMS

initial conditions	unit measure	paracetamol case	mefenamic acid case
Equipment and Operation^a^			
media resistance	m^–1^	1 × 10^7^	7.05 × 10^7^
filter diameter	mm	27	27
driving force	mbar	500	500
equipment volume	mL	50	50
Cake Resistance			
sphericity^b^		0.4127	0.4680
cake porosity^c^		0.44	0.3916
initial cake mass	g	0	0
settling index			
Initial Conditions			
mass solid phase	g	5.895	4.34
mass liquid phase	g	39.9	43.4
crystallization solvent liquid-phase mass fraction		0.88	0.93
solute liquid-phase mass fraction		0.12	0.07
wash solvent liquid-phase mass fraction		0	0
filtration temperature	°C	25	25
particle mean size	μm	77	94
particle size distribution standard deviation	μm	174	174

a Equipment geometry is equivalent to the Biotage unit system.^57^ The driving force applied is a set value in the range of driving force applied during filtration and washing processes done with the Biotage unit.

b Empirically estimated from particle size analysis.

c Empirically estimated.

Additional filtration model input parameters (e.g., suspension composition, liquid phase properties, crystal phase properties, solubility, and particle size distribution) are reported in the Supporting Information. Filtration outputs considered are filtration time (s), filtrate flow rate (m^3^ s^–1^), cake resistance (m kg^–1^), cake volume (m^3^), cake height (m), and volume of liquid trapped at the end of the filtration (m^3^). Filtration was modeled as a batch process, considering the Richardson–Zaki sedimentation equation with sedimentation occurring during filtration. Filtration is halted at dryland point (*D*) or, for models 1b and 2b, halting at breakthrough (*B*) was also simulated.

### 3.2 Washing: Equations, Assumptions, and Constrains

In all cases, the wash ratio, *W*_r_, was defined as the ratio between the volume of wash added, *V*_w_, and the volume of voids in the cake, *V*_v_. The wash ratio at a particular time point can also be related to time, *t*, by considering the superficial wash velocity, *u*_s_, and cake height, *L*
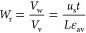
5Models 1a and 2a were designed as a simple mass balance in Microsoft Excel. Models 1b, 1c, and models 2b and 2c were simulated using gPROMS FormulatedProducts. Initial conditions, such as system information, liquid properties, crystal properties, solubility of the solid phase, and grid parameters, are reported in Tables S1–S3. The parameters for each test compound–solvent combination are reported in the Supporting Information. Model 1 results are the liquid phase composition at different washing ratio (wash curve). Model 2 outcomes instead consider not only the liquid phase composition evolution during washing but also the evolution of the particle size distribution. For model 2c, cake porosity and liquid phase saturation are also investigated as model outcomes. Particle agglomeration/breakage is not investigated in this work.

#### 3.2.1 Case 1a Purely Displacement Washing

Model 1a describes a washing process governed by displacement, where the solid phase of the suspension does not interact with the liquid phase and no dissolution or deposition is occurring. Displacement washing is a simplistic approach to model a washing process that considers the volume of liquid within the filter cake to be finite. Therefore, any added wash (Figure 1, stream 4) causes an equal removal of the mother liquor (Figure 1, stream 5). As no mixing between the wash and mother liquor is assumed, the liquid exit composition will remain constant (as the mother liquor composition) until the full volume of mother liquor in the cake has been displaced by the wash liquid. At this point, the liquid exit composition will be that of the input wash. This can be represented by the piecewise function
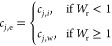
6The diameter of the filter media was used to calculate the filtration cross section (m^2^), while from the solid mass, knowing the solid density and the void fraction, the solid volume, cake volume, and the height of the cake were calculated.

Model 1a was designed as a simple mass balance in Microsoft Excel. To calculate the concentration of the different species in the liquid phase at the end of washing, the parameters listed in Table S2 were used.

#### 3.2.2 Case 1b Purely Dilution Washing

Case 1b describes a washing process in which dilution is the governing mechanism. This model assumes no solid phase variation or dissolution during the washing process. The solute present in the mother liquor phase is diluted by the addition of a wash solvent. This model is therefore used to simulate resuspension of the cake (reslurrying) by adding extra solvent (wash solvent) to the mother liquor left after the filtration process without allowing filtrate removal. Dilution washing is another simplistic approach to the model washing process which considers that any wash liquid that is added to the filter cake is instantaneously mixed with the existing liquid in the pores (mother liquor). In this regard, the liquid within the filter cake can be treated as a perfectly mixed system and is modeled by the following set of equations. The rate of change of any given species, *j*
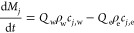
7By assuming that the exit flow rate, *Q*_e_, is equal to the wash flow rate, *Q*_w_, eq 7 can be simplified to
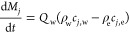
8As the system is perfectly mixed, it can be assumed that the composition within the filter cake is the same as the outlet stream
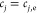
9The composition of material within the unit is, therefore, given by
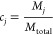
10
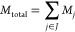
11Model 1b was built using a well-mixed crystallizer with hold-up equivalent to the cake volume predicted in the filtration model (mL) where a liquid source, a liquid sink, and a liquid composition sensor are connected. The assumptions/approximations of the model are reported in Table 3.

**Table 3:** Assumptions Used for the Dilution Washing Model

assumption/approximation	description
particle size variation	no nucleation, reaction, crystal growth, dissolution, or agglomeration is considered during the washing process
initial liquid volume	equivalent to the volume of the void volume; this parameter was predicted during the filtration simulation, as the volume of the cake formed at the end of filtration; two cases are considered: cake saturated with mother liquor (dryland), and partially deliquored cake (breakthrough)
initial liquid and solid-phase composition	equivalent to the one used during the filtration process

Further filtration model parameters are reported in the Supporting Information.

#### 3.2.3 Case 1c Diffusion–Dispersion Washing

Model 1c describes a washing process where the initial wet packed bed obtained by filtering a suspension to dryland is washed by diffusion–dispersion mechanisms. Diffusion and dispersion washing can be modeled using the main and side channel models.^31,50,51^ The assumptions used in this model are reported in Table 4.

**Table 4:** Assumptions Used for Diffusion–Dispersion Washing Model

assumption/approximation	description
mass transfer washing period	considered as the time required to replace the mother liquor left in the cake after filtration with the same volume of wash solvent; in general, in the event that the cake is fully saturated before the onset of washing, part of the solute may be flushed out by the initial charge of washing, filling the main channels; the remaining portion of the cake void volume, formed by side channels, at this stage is completely filled with residual filtrate; to remove this fraction of the mother liquor, diffusion–dilution–washing mechanisms are required
diffusional displacement	occurs during the mass transfer stage to remove filtrate from the side channels; filtrate is removed from side channels and leaves the cake by plug flow (PF) in the main channels
mixing between mother liquor and wash solvent	instant process (as assumed in model 1b); since the mixing time between wash solvent and mother liquor is approximated to zero, the diffusion coefficient used for model 1c is very small (fixed at 1 × 10^–9^)^30,31,50,51,56^

As reported by Tien,^30^ washing can be considered as a mass transfer process that takes place in porous media. However, considering diffusion and dilution washing, the diffusion of the wash solvent in mother liquor needs to be considered. Considering a homogeneous medium, with a uniform pore liquid flow rate and the diffusion–dispersion effect limited in the flow direction
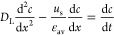
12For the initial conditions of
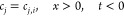
13And boundary conditions (assuming that the axial dispersion effect is ignored at the top of the cake)
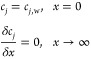
14Model 1c was implemented in two different approaches: the washing process was simulated as a plug flow (PF) crystallizer considering the diffusion–dispersion mechanisms of washing by assuming a fixed molecular diffusivity coefficient and by calculating the axial dispersion coefficient as reported by Huhtanen et al. and Tien.^30,31^ The second approach used considered the washing process simulated with a cascade of 10 well-mixed crystallizers where the approach used to mimic the dispersion washing mechanism modeled with the PF approach is clearly described by Levenspiel in the compartment models Chapter 12.^58^ Further details of the two diffusion–dispersion models developed are reported in the Supporting Information.

The input and output parameters considered for model 1c are reported in the Supporting Information.

**Figure 2:**
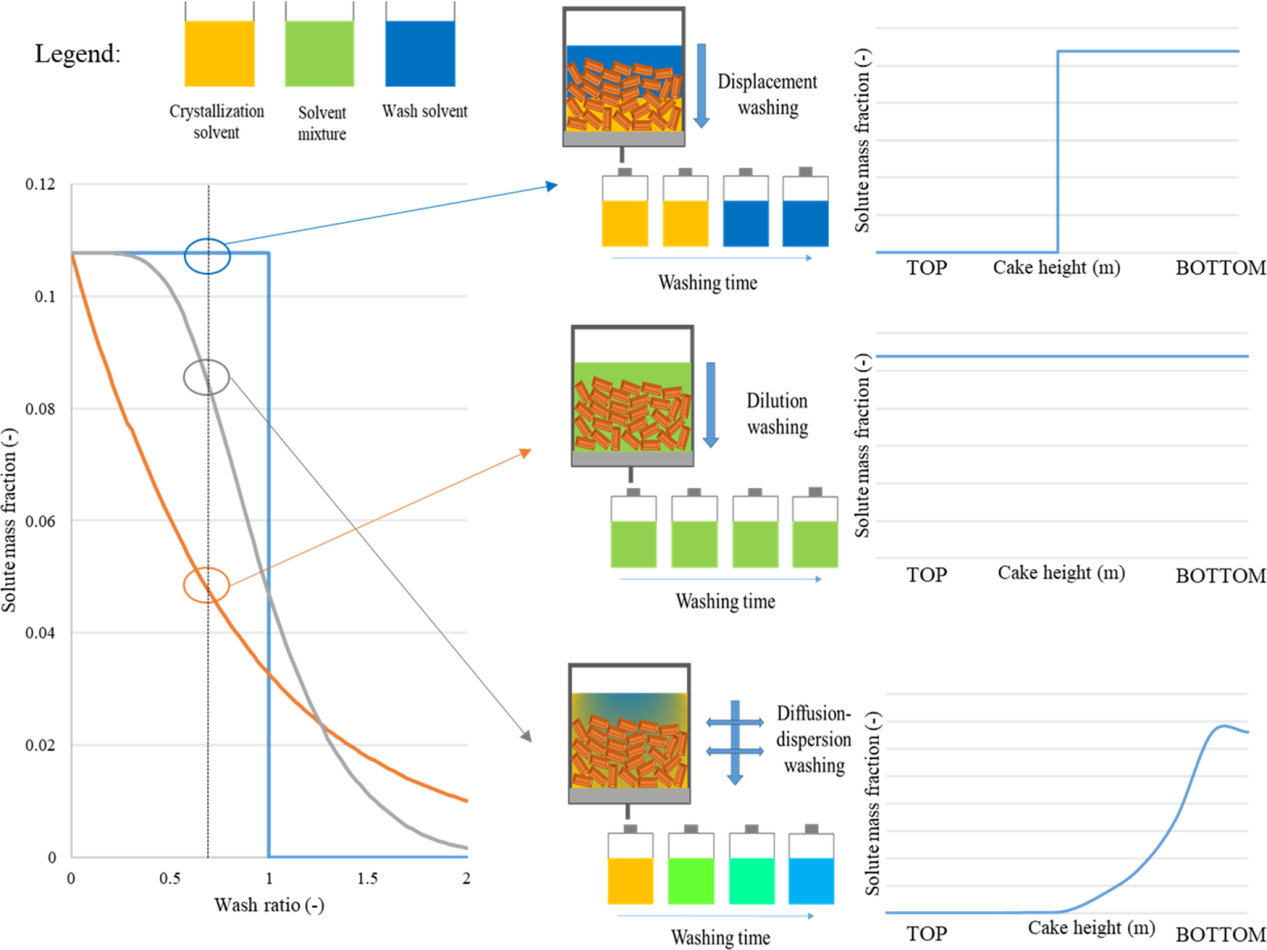
(Left) Typical profile of wash curves for displacement, dilution, and diffusion–dispersion models with no change in the solid phase. The center panel shows a schematic representation of the solvent front in the cake during washing and the composition of the filtrate solvent. The mass fraction is calculated as the solute concentration in the filtrate over the solute concentration in the mother liquor at the end of the crystallization. The wash ratio is defined as the volume of the wash solvent over the void volume of the cake that evolves during the washing time. (Right) Typical solute mass fraction evolution along the cake height for displacement, dilution, and diffusion–dispersion washing mechanisms.

A schematic comparison of these different case 1 approaches is shown in Figure 2.

#### 3.2.4 Case 2a Displacement with Soluble Wash Solvent

In case 2a, the equations used correspond to the equation reported for model 1a. Case 2a is designed to model pure displacement washing where the wash solvent shows non-null compound solubility, thus causing the partial dissolution of the solid phase during the wash solvent passage through the cake. The dissolution process is considered homogeneous across the cake. As for model 1a, also in model 2a, one equivalent cake volume of wash solvent is used to wash the cake.

The assumptions used in this model are reported in Table 5.

**Table 5:** Assumptions Used for Displacement Washing Model with Solid-Phase Dissolution

assumption/approximation	description
particle size variation	this is estimated from the solubility of the solid phase in the wash solvent and the volume of the dissolved particles; from the solubility, the volume of material dissolved from the particles is estimated (mass of solid dissolved converted in volume), and therefore the modified diameter of the particles is calculated

The model allows for the simulation of the crude wash curve (pure mass balance of the different liquid-phase species) and the simulated particle size reduction after the homogeneous dissolution process.

#### 3.2.5 Case 2b Instant Complete Liquid-Phase Mixing, Dilution, and Dissolution of Solid Phase

Model 2b combines the model approach described in case 1b with the dissolution model (see the Supporting Information). Dissolution of the solid phase can be included through the inclusion of additional equations. The widely used Nernst–Brunner equation^59^ relates the dissolution rate to the diffusion coefficient
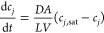
15Taking into account the total mass of the liquid phase, eq 7 can be modified to consider the change in the mass of a species. This can then be incorporated into eq 8 resulting in eqs 16 and 17, respectively.
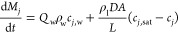
16
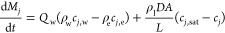
17To verify that the system is in equilibrium through the washing process, the evolution of the solute concentration (*c*_*j*_) during washing is compared with the solubility evolution (*c*_*j*,sat_). This model is able to identify the risk of API cake dissolution and of solute deposition due to the progressive variation in the system solubility during washing. The use of binary solubility plots enables the simulation of stream 5 composition at increasing wash: mother liquor solvent ratios. This model is based on a series of assumptions (Table 6).

**Table 6:** Assumptions Used for Diffusion–Dispersion Washing Model

assumption/approximation	description
miscibility of mother liquor and wash solvent	instantaneous process across the entire cake volume; the composition of the liquid phase inside the cake instantly evolves from pure mother liquor to pure wash solvent solution
amount of API dissolved	calculated from the volume of solvent used for each washing
particle size variation	● empirical power law growth/dissolution expression to model solid-phase dissolution^60^ with activation growth/dissolution energy set to zero
	● growth/dissolution rate constant set to a value to ensure the system remains in equilibrium throughout the entire washing process
	● for each solvent combination, the same selected value of the growth/dissolution rate constant was used

#### 3.2.6 Case 2c Gradient Diffusion and Dispersion with Dissolution

This model describes washing as 10 layers of solvent each of equal thickness, summing together to the thickness of the cake but of varying composition where in each layer instant mixing of the two liquid phases occurs. This model combines the four different washing mechanisms: diffusion, dilution, dispersion, and dissolution. The liquid composition in each layer evolves over time from pure mother liquor to pure wash solvent. This model is based on a series of assumptions (Table 7).

**Table 7:** Assumptions Used for Diffusion–Dispersion Washing Model

assumption	description
cake layer composition	● layer 1 corresponds to the liquid phase adjacent to the surface of the cake, while layer 10 is the layer near the filter media; at the beginning of the washing process, layer 1 is made of pure wash solvent, while layer 10 is made of pure mother liquor
	● the liquid composition of each layer changes following the binary plot solubility curve in layer 10, the liquid composition gradually moves from pure mother liquor to pure wash solvent

To simulate the evolution of the liquid and solid phases, the model was designed as 10 different continuous stirred reactors (CSTR) in series with dimensions combining to be equivalent to the volume of the cake volume predicted in the filtration model (mL). Model 2c input and output parameters are reported in detail in the Supporting Information.

**Figure 3:**
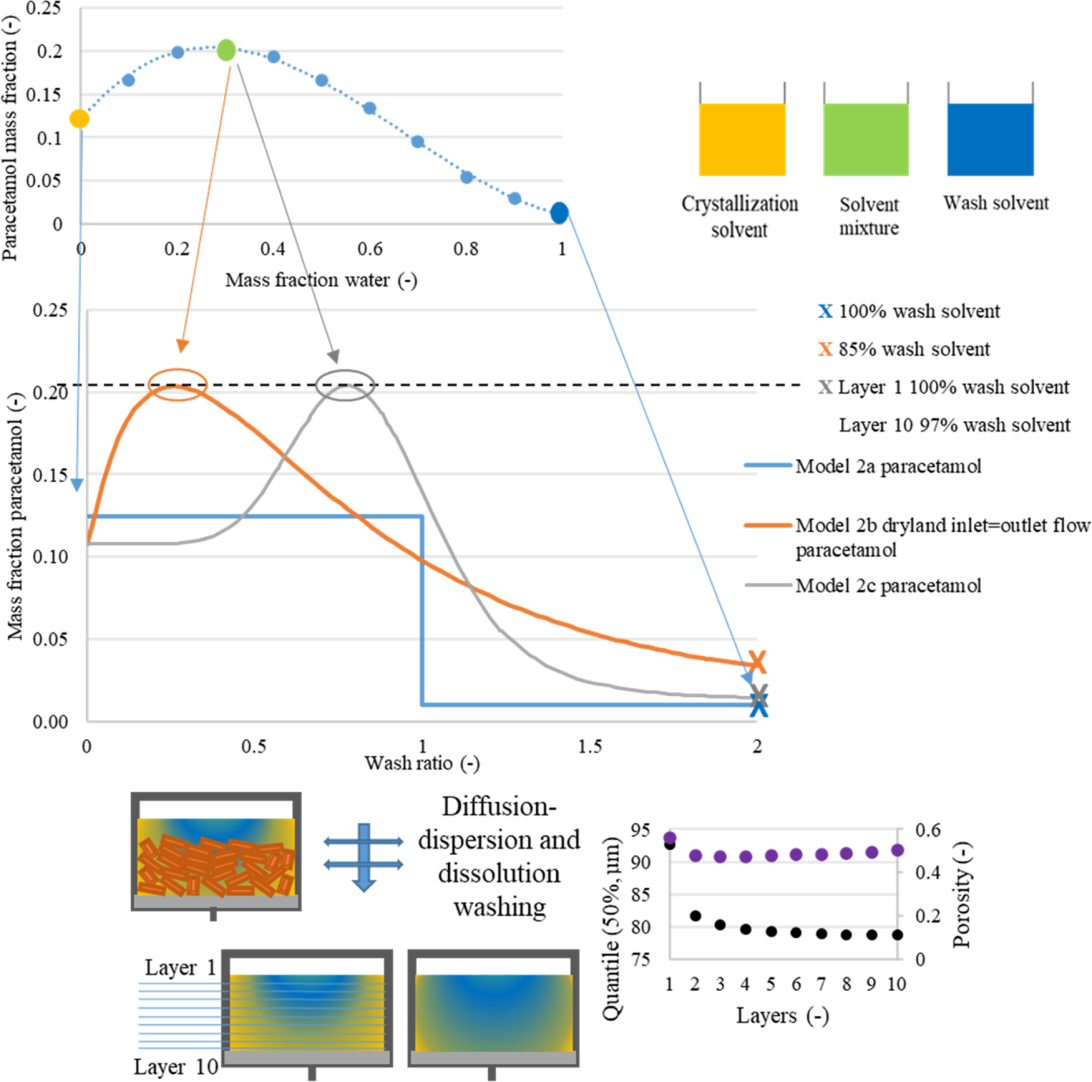
Evolution of the paracetamol solubility profile evolution for the case where isopropanol is used as a crystallization solvent and water is selected as a wash solvent. (Middle) Schematic wash curves for displacement, dilution, and diffusion–dispersion models with instantaneous solid–liquid equilibration. (Top) Schematic representation of the solvent front in the cake during washing and composition of the filtrate solvent. (Bottom) Schematic representation of the front of the solvent in the cake during washing and the composition of the filtrate solvent composition.

A schematic comparison of these different approaches to model washing is shown in Figure 3; the gray line represents the wash solvent, the blue line represents mother liquor, and the orange line represents the solid phase of the suspension.

### 3.3 Solubility Prediction

The solubility of paracetamol and mefenamic acid in their respective crystallization and wash solvents was predicted using COSMOtherm (COSMOlogic GmbH & Co. KG, Germany), and where available, they were taken from the literature.^61−64^ Miscibility data for all solvent combinations were calculated within COSMOtherm and verified/corrected where data from the literature were available.

The solubility binary plots of paracetamol in isopropanol–water/heptane/dodecane/acetonitrile and mefenamic acid in 2-butanol–heptane mixtures are reported in the Supporting Information.

## 4 Results and Discussion

### 4.1 Filtration

Two different filtration-stopping conditions were simulated: filtration was stopped in dryland, and filtration stopped at breakthrough. As reported in Section 3.1, stopping filtration at dryland means that the cake pores are kept fully saturated with the mother liquor, while stopping filtration at breakthrough means that the cake pores are partially free of mother liquor and are partially filled with gas. For this work, it was assumed that for the breakthrough case, the filtration stopped when the filtered cake was deliquored until 90% of mother liquor was removed. Filtration simulations provided information on the filtration process (filtration time, filter flow rate) and information on the filtered cake (cake resistance, volume of the cake, volume of liquid trapped after filtration, and cake height). The results for the paracetamol and mefenamic acid cases are reported in the Supporting Information. The volume of the cake, the volume of liquid trapped after filtration, and the height of the cake were used as input parameters for the washing simulations.

### 4.2 Comparison of the Case 1 Model (No Changes in Solid Phase)

In this section, the concentrations of the solute phase are compared across different models to identify their strengths and weaknesses and to highlight their applications to model washing. Furthermore, these comparisons help to understand how the different washing mechanisms affect the shape of the wash curve. Two different cases were simulated to identify how the shape of the curves changes as the solvent mixture solubility curve shows a maximum or alternately decreases moving from the mother liquor to pure wash solvent.

Model 1a is a simple mass balance consisting of an initial feed made with the mass fraction of the solute and crystallization solvent, with the solute concentration corresponding to the solubility of the compound in the crystallization solvent, under saturation conditions. As model 1a is used to simulate pure displacement with immiscible solvents, where the API is insoluble in the wash solvent, complete wash is reached when the wash ratio is equal to 1, causing the solute and crystallization solvent mass fraction in the filtrate to drop to zero. Model 1a can be used to simulate cases where the solid phase is made of large particles (low cake tortuosity) with minimal risk of mother liquor entrapment, and the mother liquor and wash solvent are completely immiscible.

Model 1b, which assumes no solubility of the test API compounds in the wash solvent, was simulated with three different filtration and washing conditions, which are fully described in the Supporting Information.

Model 1c can simulate with good accuracy the three different stages of washing: the constant rate, the intermediate stage, and the diffusion stage.

**Figure 4:**
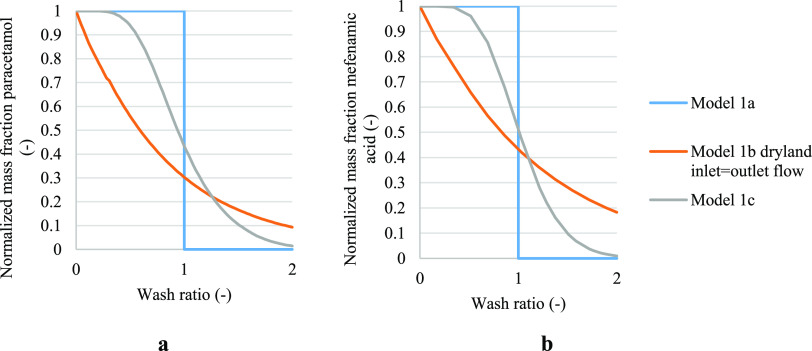
Normalized solute concentration obtained from models 1a and 1b in case of cake formed with the filtration process stopped at dryland, and where inlet and outlet flow during washing are matching, and model 1c. (a) Paracetamol where isopropanol was the crystallization solvent and water the wash solvent. (b) Paracetamol where isopropanol was the mother liquor solvent and heptane was the wash solvent.

In Figure 4, to achieve complete mother liquor and dissolved solute removed, more than 2 equiv cake volumes of wash solvent are required. This inefficient removal of the mother liquor is a consequence of the model’s complete mixing of the two liquid phases in this model. This model is a simplistic tool that simulates the back-mixing process occurring during resuspension washing. During a physical displacement/dilution washing process, only a limited layer of contact between mother liquor and wash solvent is subjected to back-mixing. In contrast, model 1b assumes that the back-mixing phenomenon occurs throughout the entire liquid phase volume and complete mixing of the two liquid phases is considered to be instantaneous. Figure 4 reports the case where filtration was stopped at dryland and the inlet and outlet flows during washing were matched. This case was selected to allow comparison with the other two models since model 1a and model 1c both consider a cake filtered to dryland and where the cakes were drained during washing. Models 1a and 1c show similar final solute concentrations. Since the assumptions selected in model 1b do not represent a real washing process, this model is better suited to simulate dilution processes. To simulate the washing curve of a washing process where the solid phase is made of nonsoluble particles, the combination of all three mechanisms (displacement, dilution–dispersion, and diffusion mechanisms) is required. Therefore, model 1c is applicable when the mother liquor and the washing solvent are miscible and particles are insoluble in the wash liquid phase.

### 4.3 Comparison of the Case 2 Model (Dissolution and/or Growth in Solid Phase)

Model 2a is comparable to model 1a, as reported in the Supporting Information. However, this simple model can identify, in a crude way, the risk of solid phase dissolution, since, for model 2a, the API is soluble in the wash solvent. As for model 1a, this is a simple mass balance tool. At a wash ratio of 1, complete removal of the mother liquor is achieved, and the cake is then fully saturated with wash solvent in which part of the solid API is dissolved. The concentration of the API dissolved in the wash solvent corresponds to the API solubility in the wash solvent at the wash temperature. Model 2a allows calculation of the variation in particle size, as indicated in Table 8, considering the mass of loss from the solid fraction due to API solubility in the wash solvent. Model 2a is appropriate when the solid phase is made of large particles, where the cake shows low tortuosity with minimal risk of mother liquor entrapment, and the mother liquor and wash solvent are completely immiscible, but both show measurable API solubility.

**Table 8:** Mean Particle Size Simulated at the End of Washing for Models 2a and 2b Where the Cake Was Filtered to Dryland and during Washing the Inlet and Outlet Flow Coincided and for Model 2c for the Paracetamol–Water and Paracetamol–Heptane

model	D50 (μm) paracetamol–water	D50 (μm) paracetamol–heptane
raw material	77	77
2a	77	77
2b dryland, inlet flow matches outlet flow	83	76.62
2c	80.82	77.04

**Figure 5:**
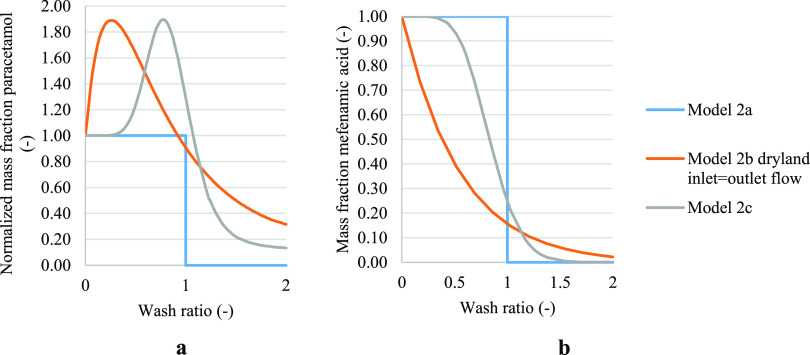
Normalized solute concentration obtained from models 2a and 2b in case of cake formed with filtration process halted at dryland, where inlet and outlet flow during washing are matched, and model 2c. (a) Paracetamol where isopropanol was the crystallization solvent and water was the wash solvent. (b) Paracetamol where isopropanol was the mother liquor solvent and heptane was the wash solvent.

Even if models 2a, 2b, and 2c have the same initial solute concentration, none of the models reach the same solute concentration at the end of the washing process. This is mainly due to models 2b and 2c that predict the extent of solid phase dissolution, which causes an increase in the solute mass fraction in the liquid phase during washing. Model 2b considers the dilution and dissolution process throughout the cake volume. Because the solubility of the test compound changes uniformly throughout the volume of the cake, the final particle size distribution simulated is the average value for all of the particles that make up the cake. Dissolution is observed only in cases where the test compounds’ solubility binary plot shows a maximum (an increase of the solubility in the mixed solvents with respect to the pure crystallization or wash solvent). Therefore, dissolution is observed in the paracetamol isopropanol–water case (Figure 5a) and paracetamol isopropanol–acetonitrile case (Figure S24) cases. For paracetamol isopropanol–heptane case (Figure 5b), the paracetamol isopropanol–dodecane case (Figure S22), and the mefenamic acid case (Figure S25) no dissolution occurs. The dissolution process, if it occurs, is then followed by dilution of the enriched solute phase, as further wash solvent is added. Model 2c considers diffusion–dilution with dissolution washing mechanisms. The first part of the simulated curve corresponds to the constant-rate period, where no diffusion–dilution and dissolution process is occurring, meaning that the dissolution process is observed only when the falling rate period starts. Model 2b can be used to simulate washing processes in which the evolution of the liquid composition is homogeneous throughout the entire cake volume and solid phase dissolution occurs in combination with the dilution washing mechanism.

### 4.4 Comparison of Dilution (b) and Diffusion (c)

**Figure 6:**
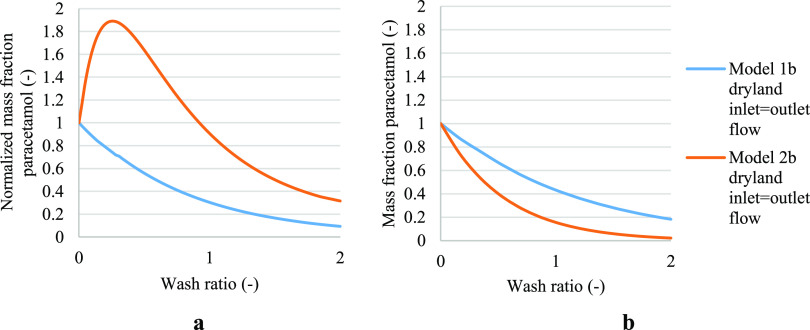
Normalized solute concentration obtained from models 1b and 2b in case of cake formed with the filtration process stopped on reaching dryland, where inlet and outlet flow during washing are identical. (a) Paracetamol was selected as a test compound, where isopropanol was the crystallization solvent and water was the wash solvent. (b) Paracetamol was selected as the test compound, where isopropanol was chosen as the mother liquor solvent and heptane was chosen as the wash solvent.

**Figure 7:**
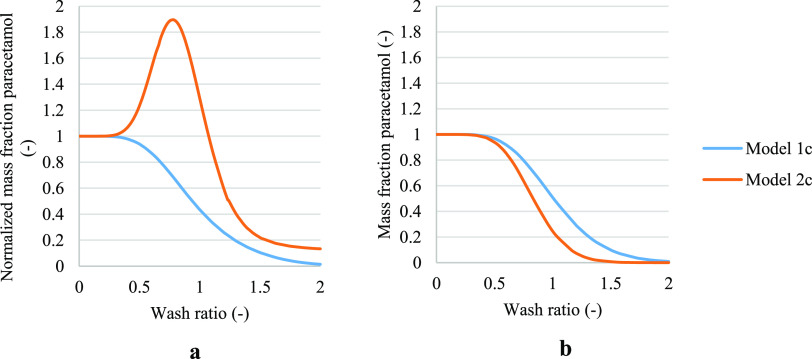
Normalized solute concentration obtained from models 1c and 2c. (a) Paracetamol was selected as a test compound, where isopropanol was the crystallization solvent and water was the wash solvent. (b) Paracetamol was selected as the test compound, where isopropanol was chosen as the mother liquor solvent and heptane was chosen as the wash solvent.

For completeness, cases 1b and 2b are compared in Figure 6 and cases 1c and 2c are compared in Figure 7. These figures clearly show the difference between case 1 and case 2 models, where in case 2 dissolution is also taken into consideration in the washing mechanism.

### 4.5 Particle Size and Porosity Evolution

**Figure 8:**
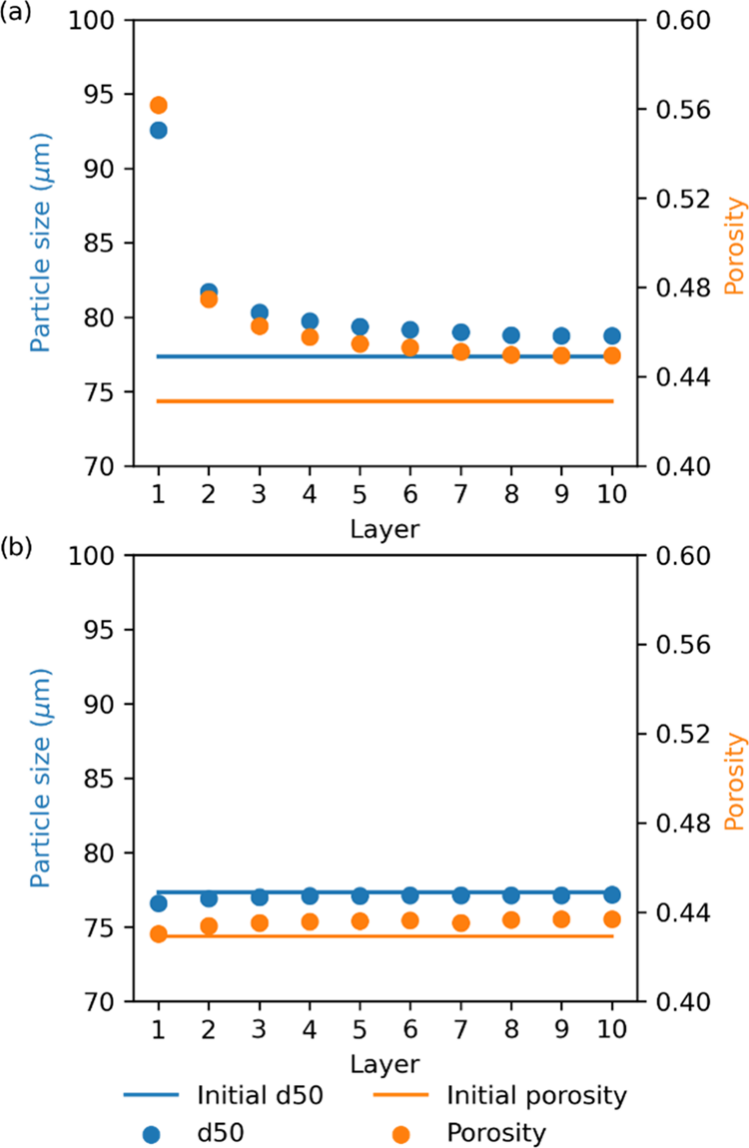
Mean quantile particle size at 50% (blue dots) and variation in cake porosity (orange dots) during washing for model 2c for *W*_r_ = 2.0. (a) Paracetamol was selected as a test compound, where isopropanol was the crystallization solvent and water was the wash solvent. (b) Paracetamol was selected as the test compound, where isopropanol was chosen as the mother liquor solvent and heptane was chosen as the wash solvent.

**Figure 9:**
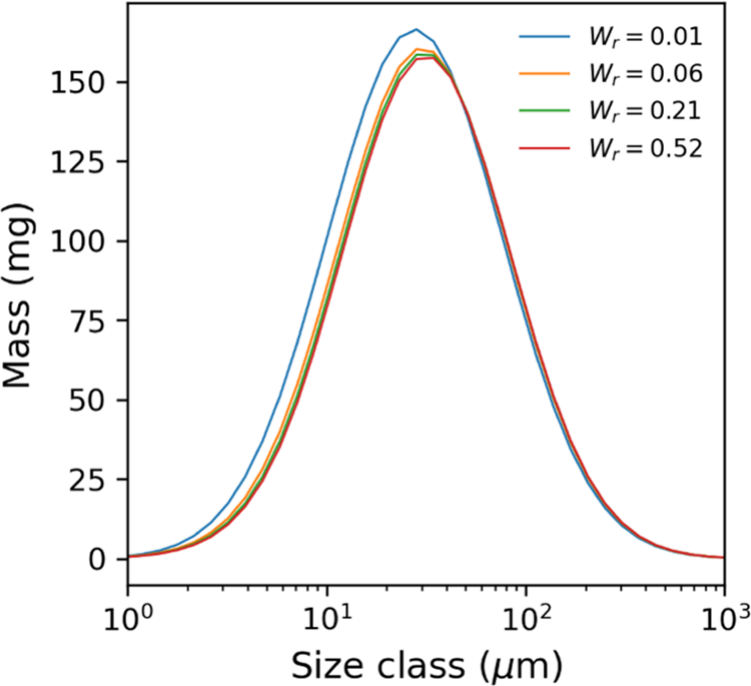
Mass distribution of particles in layer 1 in the first stages of washing for the case of paracetamol isopropanol–water

**Figure 10:**
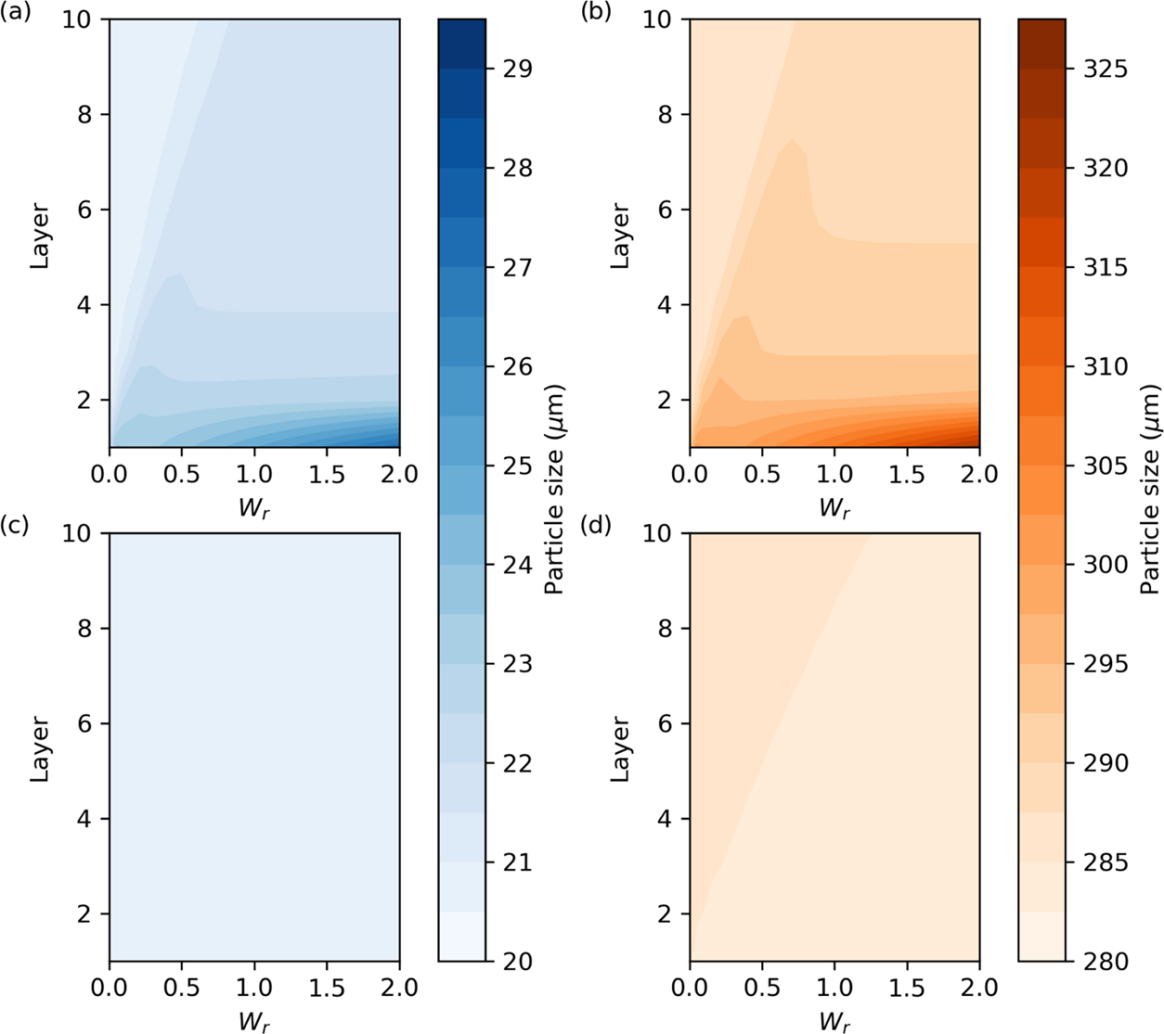
Variation in the particle size percentile (10th percentile and 90th percentile) between the different cake layers during the washing process, at different wash ratios for model 2c. (a) 10th percentile variation in particle size between the different layers of the cake during washing for the paracetamol isopropanol–water. (b) Variation in the particle size of the 90th percentile variation of between the different layers of the cake layers during washing for the case of paracetamol isopropanol–water case. (c) 10th percentile particle size variation across the different layers of the cake layers during washing for the paracetamol isopropanol–heptane case. (d) 90th percentile particle size variation across the different cake during washing for the paracetamol isopropanol–heptane case.

Models 2a, 2b, and 2c allow the particle size variation caused by solid fraction dissolution to be simulated (see Table 8), with models 2b and 2c showing improved accuracy in simulating the mean particle size of the washed cake. Furthermore, model 2c can predict the mean size of particles for the different layers of the cake (see the Supporting Information) (Figure 8), and at different wash ratios (Figure 10). The particle size distribution value reported is the 50th percentile (sometimes referred to as the median particle size) of the entire size distribution. The porosity of the cake was extrapolated from the simulated data based on the liquid mass left in the cake at the end of washing. To calculate the liquid mass trapped in the cake, the void volume of the cake was assumed to be equal to the liquid volume left in the cake, and the liquid density in each layer was calculated based on the predicted local liquid composition. Figure 8a presents the paracetamol isopropanol–water where the binary solubility plot of the API shows a maximum, and in this case variations in particle size and porosity are observed. This contrasts with the case of paracetamol isopropanol–heptane, Figure 8b, where the binary solubility constantly decreases, and the particle size and porosity remain at their initial values in all cake layers. In the case of paracetamol isopropanol–water, at first consideration, one would expect the particle size to decrease with an increase in solubility leading to dissolution. However, Figure 8a shows that after washing, *W*_r_ = 2.0, the particle size is larger across all layers, but most significantly in the uppermost layers (1–5) than before washing. Similarly, there is also an increase in porosity across all layers compared to the initial conditions. To understand this, Figure 9 shows the mass distribution of the particles within each size class for layer 1 in the initial washing stages. Here, the left-hand side of the distribution disappears quickly when moving from *W*_r_ = 0.01 to 0.06. This is due to the smaller particles having a greater specific surface area compared to the larger particles, leading to the preferential dissolution of the small or fine particles. As a result, the mass distribution effectively shifts to the right, resulting in increased median particle size.

To further examine the dissolution of particles, Figure 10 shows how the 10th and 90th percentiles of the particle distribution change with wash ratio and cake layer. Figure 10a,b shows the 10th and 90th percentiles, respectively, for the paracetamol isopropanol–water case. As with Figure 8a, a change in percentile is observed between the layers and with the wash ratio. This contrasts with Figure 10c,d, which shows the 10th and 90th percentiles, respectively, for the paracetamol isopropanol–heptane case, where little to no change in the particle size distribution percentiles was observed. As expected, in Figure 10a,b, the greatest change in particle size percentiles is observed at the upper layers (1–3) and at the highest wash ratios. This is because these upper layers are subject to the most fresh wash solvent and therefore the highest driving force for dissolution. The wash becomes progressively more saturated as it moves through the cake.

### 4.6 Evolution of the Cake Mass

**Figure 11:**
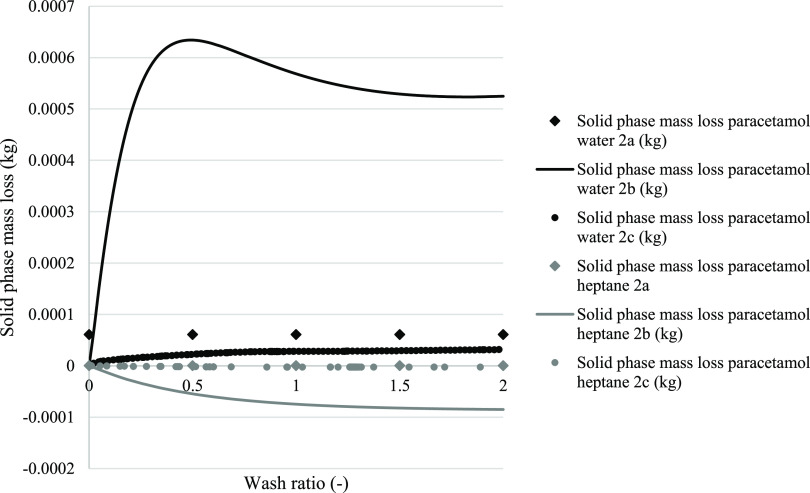
Simulation of solid phase mass loss during washing. In black, paracetamol was selected as the test compound, where isopropanol was the crystallization solvent and water was the wash solvent. In rhomboid dots, the solid loss for model 2a is reported. For model 2b, circle dots were used. Straight-line solid loss for model 2c is reported. For model 2c, this graph shows a gradual dissolution of the solid phase during washing. The total loss of mass of the solid phase corresponds to 31.1 mg. In dark gray, paracetamol was selected as the test compound; isopropanol was chosen as the mother liquor solvent and heptane was chosen as the wash solvent. For model 2c, this graph shows a gradual increase in solid phase mass due to solute phase deposition. The total increase in solid phase mass corresponds to 3.13 mg.

Figure 11 presents the predicted mass loss (or gain) that occurs during the washing procedure using models 2a, 2b, and 2c to predict for two cases, both paracetamol crystallized from isopropanol, one washed with water and the other washed with heptane. This allows the predicted impact of washing on the loss of product (i.e., yield) to be compared for each model. The two wash solvents represent the two opposite phenomena that occur in industrial practice. Dissolution when the product exhibits solubility in the wash solvent, in this case water, and where there is a maximum in solubility in mixtures of crystallization solvent and wash solvent. The deposition that occurs when the product solubility in the wash solvent is negligible and the addition of the wash solvent to the saturated crystallization solvent causes an antisolvent effect, resulting in further product being driven out of solution.

**Figure 12:**
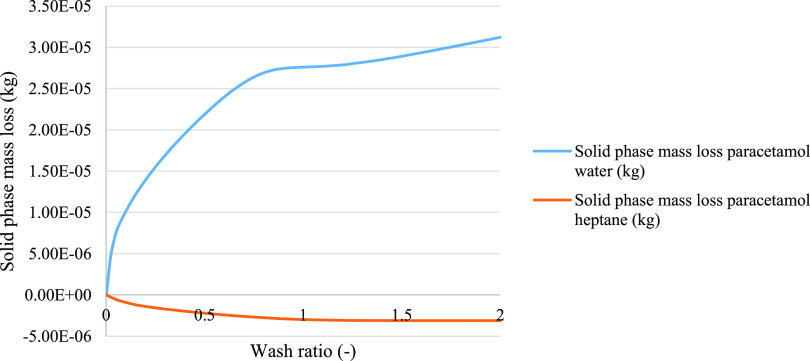
Details of the mass loss of the solid phase simulated with model 2c during washing. In blue, paracetamol was selected as a test compound, where isopropanol was the crystallization solvent and water was the wash solvent. In orange is the solid phase mass loss simulated for model 2c, where paracetamol was selected as the test compound, isopropanol was used as the crystallization solvent, and heptane as used as the wash solvent.

Model 2a predicts an initial modest but “instantaneous” dissolution of 60.8 mg for washing with water, while for washing with heptane a negligible amount of product deposited by the antisolvent effect. Model 2b predicts much larger changes, for the case of washing with water the quantity of product dissolved peaks at 633 mg at a wash ratio of around 0.5 before some of that dissolved material is reprecipitated as washing continues to completion with the final predicted product loss being around 525 mg, almost an order of magnitude greater than for model 2a. Similarly using model 2b for the case of washing with heptane, the predicted antisolvent effect is substantially greater than predicted by model 2a, 85 mg being deposited compared to just 3.1 mg. The product deposition profile was initially more rapid at the start of washing and then slower later, consistent with that anticipated in an antisolvent deposition process. Model 2c predicts a gradual increase in product loss when washing with water. From Figure 12, model 2c for the paracetamol isopropanol–water case exhibits the characteristic dissolution peak seen in model 2b, but rather the quantity lost is modest and shifts to a higher wash ratio, showing that the loss rate is initially much slower than from model 2b. The rate decreases as the washing continues to completion, the maximum predicted product loss is 31.1 mg, approximately half that predicted by model 2a. For washing with heptane, model 2c predicts a similar minimal increase in product mass of 3.13 mg to that predicted by model 2a.

Model 2c was designed to have the cake separated into 10 layers, where the evolution of the dissolution of the cake during washing is progressive through the passage of wash ratio 2 through the cake. When this is expressed as the amount of API lost (dissolved) in Figure 11, the calculated quantity lost is determined across all 10 layers even though at the beginning of washing only the first of the 10 layers has been affected by the washing process. Progressively, as the wash ratio increases, the changes in the quantity dissolved are summed across an increasing number of layers, and, for the earliest layers, the combined impact of material dissolution across multiple time increments is consolidated. A consequence of this is that there is a delay in the quantity dissolved response relative to the wash ratio. As observed in Figure 11, the solid phase mass lost simulated with model 2c during washing, for the paracetamol isopropanol–water (black line) and for the isopropanol–heptane case (gray line) are plotted with respect to the wash ratio. The total mass loss of the solid phase corresponds to 31.1 mg for the isopropanol–water case, showing a consistent solid phase loss due to the dissolution mechanism. Figure 11 shows an initial loss of solid phase during the first part of the wash before reaching a wash ratio of 1. This is aligned with the evidence reported in Figure 7a (orange plot), where a consistent increase in solute concentration is observed between wash ratios 0.5 and 0.8. A second process resulting in a reduction in the mass of the solid phase lost is observed after a wash ratio of 1. This change is modest in relation to the initial dissolution and is a consequence of the predicted reduction in API solubility in a wash solvent-rich liquid mixture (as reported in Figure S1). For the paracetamol isopropanol–heptane case, instead, the total gain in mass of the solid phase corresponds to 3.13 mg, showing potential antisolvent effect, with deposition of part of the solute present in the liquid phase. The deposition trend observed in Figure 11 (gray line) is similar to the dissolution trend observed for the case of paracetamol isoporopanol–water. An initial deposition, with a considerable mass gain (approximately 2.95 mg) is observed before reaching a wash ratio of 1, followed by a second deposition phase with a mass gain of 0.16 mg of paracetamol. This is correlated with the initial solubility drop observed in the first part of the solubility binary plot of the paracetamol isopropoanol–heptane plot (Figure S2), dropping from 0.10 g g^–1^ to approximately 0.01 g g^–1^ (10 times). The second solute deposition is less pronunced and corresponds to a minimal drop in API solubility (Figure S2).

Overall, the three models predict this different magnitude of effects because of the different methods used to simulate the solvent mixing effect throughout the cake thickness during washing. In model 2a, there is no mixing between crystallization and wash solvent and the two species are clearly separated across the cake thickness. In model 2a, solid phase dissolution occurs only in the cake portion of the cake where the wash solvent is present. Furthermore, the dissolution in model 2a is considered an instantaneous process, bringing the amount of lost solid phase constant and equal to the API solubility in the wash solvent during the entire washing process. In model 2b instead, the liquid phase composition is considered uniform across the entire cake and changes during washing, passing from pure crystallization solvent to pure wash solvent. The mass loss of the solid phase changes during the washing process, the trend of dissolved solid phase during washing is therefore correlated to the API solubility profile in case of mixed solvents. In model 2b, the amount of solid phase lost before a wash ratio of 1 is dictated by the shape of the API solubility curve: in case of maxima, the solid phase mass lost increases reaching a maximum, which is characteristic of a dissolution effect; in case of an API solubility curve with a drastic solubility drop from pure crystallization to wash solvent, risk of solute deposition is observed with a gain of solid phase mass. After a wash ratio of 1 is reached, for model 2b, the case of solid mass lost/gain reduces/stabilizes because of the dilution effect: after the solid phase variation, the extra amount of liquid added just produces a dilution of the liquid phase with no extra dissolution/deposition effect. In model 2c, the liquid phase composition is not uniform across the cake thickness, changing from the cake top to bottom layer and during washing time. This gradual phase composition variation, which better resembles the physical scenario observed during a washing, mitigates the dissolution/antisolvent effect during time, shifting these effects to longer washing times. Moreover, the discrepancy of solid phase mass lost/gain observed from model 2b and model 2c is directly related to the approach used to simulate the liquid phase composition variation during washing. In model 2b the entire cake is subjected to a homogeneous variation of liquid composition, therefore the amount of solid phase subjected to dissolution effect is higher with respect to model 2c. On the other hand, in model 2c only a few portions of the cake (layer 1 and few above) are subjected to the liquid phase composition variations that enable dissolution/deposition effects. Therefore, in model 2c, the portions of cake exposed to the dissolution/antisolvent effect are limited to the layers with an initial crystallization solvent-rich composition. Overall, model 2c can be used to simulate washing processes where the solid is soluble in the combined liquid phase because of its capability to better represent the liquid and solid phase composition variation occurring during a physical washing process. No agglomeration or deposition is explicitly modeled in any of the approaches reported, though extending the models to include these phenomena could be considered using the mass removal/deposition values generated in the models, though other aspects of these phenomena would need to be incorporated for the models to be likely to match experimental observations.

## 5 Filtration and Washing Model Selection Guideline

**Figure 13:**
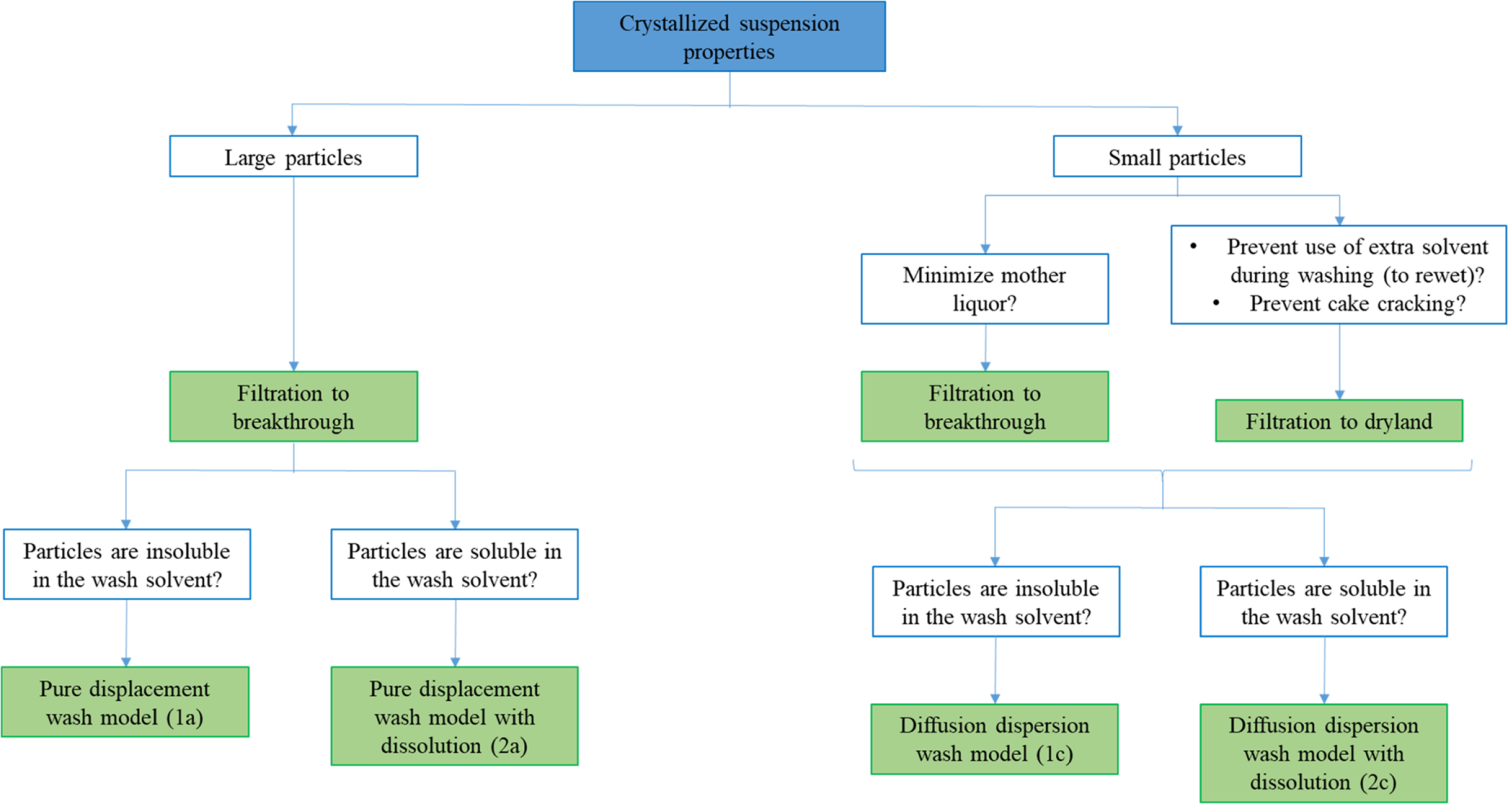
Guideline for filtration and washing models based on the crystallized suspension.

This work developed a series of filtration and washing models with different assumptions and varying levels of complexity to allow the selection of the filtration and washing model that best fits the crystallized suspension characteristics. Figure 13 proposes a guideline for smart model selection based on the properties of solid and liquid phases and their interactions in the slurries to be isolated.

On the basis of the particle size of the solid phase, a filtration model can be selected with filtration end point either to dryland or breakthrough. In case the solid phase consists of large particles, with the cake formed showing large porosity, fast filtration, and chance of partial or total cake deliquoring is generally achieved. Therefore, for this type of suspension, it is suggested that the filtration to breakthrough modeling tool is used. However, in the case of a suspension with a solid phase formed of small particles, both filtration models to breakthrough or to dryland can be appropriate. In this case, the selection of the most suitable model is dictated by the objective of isolation. If the target is to minimize the amount of mother liquor and impurities trapped in the cake, to facilitate the washing process, it is recommended that the filtration to breakthrough model is used. Otherwise, where cake cracking/shrinking or changes to the cake occurring during the deliquoring of the cake need to be avoided, modeling filtration halted at dryland is recommended. Another reason to halt filtration at dryland is the minimization of solvent use during the washing process, eliminating the solvent required for the cake rewetting of deliquored cakes.^38^

The criteria used to select the appropriate washing model are based on the nature of the solid–liquid phase interaction. If the crystallization phase is immiscible with the wash solvent, and therefore the solubility binary plot of the solid phase does not show a maximum, washing models that do not consider dissolution as one of the washing mechanisms can be selected. Indeed, in the case of large product particles, the pure displacement model (1a) is recommended for its simplicity and ability to simulate with good accuracy the solute and liquid species composition evolution during washing. In the case of small particles, the diffusion–dispersion model (1c) is applicable when mother liquor and wash solvent are miscible, and particles are insoluble in the liquid phase. This model is suggested in the case of large tortuosity and the risk of mother liquor entrapment and is designed to simulate the evolution of the solute and liquid phase species in the case where the wash solvent diffuses and dilutes the mother liquor trapped in small cake cavities and then displaces it.

A similar selection criterion is suggested in cases where the solid phase can dissolve in the crystallization-wash solvent mixture during washing. In the case of large particles, pure displacement with a dissolution model can be used to simulate solute and liquid species evolution during washing. Furthermore, this model is capable of predicting the risk of particle size reduction caused by the solid phase dissolution. In the case of small particles, instead, the diffusion/dispersion with dissolution model (2c) is suggested to simulate the evolution of liquid phase composition, and the solid phase evolution (particle size distribution and porosity variations) during washing.

## 6 Conclusions

An integrated approach was developed to simulate filtration and isolation processes to facilitate integrated end-to-end pharmaceutical manufacturing using digital design. The aim of this work was to predict the combined filtration and washing operations using a semiempirical approach to simulate filtration and washing performance. Two limiting cases were developed in this work; case 1 where changes in solid phase are not considered, and case 2 where the solid phase changes are considered. Three different modeling approaches are used for each case to describe different washing mechanisms: displacement (a), dilution (b), and diffusion–dispersion (c).

In this paper, we proposed two dead-end filtration models, addressing filtration halted at dryland and continued to breakthrough along with six washing models subdivided into two cases of increasing system complexity. The Carman–Kozeny equation is used to model cake resistance and the Darcy equation to model filtration time and filtrate flow rate. The simulated responses of the filtration models (filtration time, filtrate flow rate, and the composition of the filter cake and filtrate generated during filtration) were then used as input parameters for the washing models.

No agglomeration or deposition was modeled. The responses simulated with the different washing models were related to washing efficiency, where the models were used to generate washing curves, cake, and filtrate composition, to indicate the probability of particle size and cake porosity variation caused by cake dissolution, and to predict residual cake moisture content and composition.

Representative model compounds, mefenamic acid and paracetamol, were used to demonstrate the prediction capability of the filtration and washing models developed. For mefenamic acid, 2-butanol was selected as the crystallization solvent and heptane was used as the wash solvent. For paracetamol, isopropanol was used as the crystallization solvent, whereas a series of wash solvents were used to show the different washing phenomena. Water and acetonitrile were selected because the APIs exhibit some modest solubility to exemplify API dissolution during washing. Heptane and dodecane were selected because APIs exhibit negligible solubility.

Comparing the simulations for representative model compounds, mefenamic acid and paracetamol, allowed the strengths and limitations of the different models to be explored. A filtration and washing model selection guideline has been developed to indicate where each of the different washing models might be applicable for different processing conditions and using the predictions to explore how the different washing mechanisms affect the shape of the wash curve and the extent of particle dissolution.

## Supporting Information Available

Additional modeling input conditions; materials and methods; and additional results, including graphs and results description (PDF)

## Notes

All data underpinning this publication are openly available from the University of Strathclyde KnowledgeBase at 10.15129/2d75a4a8-83f1-4640-810d-47d963662a33

## Abbreviations

API: Active Pharmaceutical Ingredient
CMAC: Continuous Manufacturing and Advanced Crystallization Future Manufacturing Research Hub
PSD: particle size distribution
*D*: filtration halted to dryland
*B*: filtration halted to breakthrough
PF: plug flow
CSTR: continuous stirred-tank reactor
D50: cumulative 50% point of diameter

## Nomenclature

*A*: filter area (m^2^)
*c*: dry cake mass per unit volume of filtrate (kg m^–3^)
*c*_0_: solute concentration of the filtrate at the beginning in the cake
(*c*_w_)_I_: wash solvent concentration
(*c*_w_)_e_: effluent concentration
*c*_s_: solute concentration at saturation condition (kg kg^–1^)
*c*_*t*_: solute concentration at specific time (kg kg^–1^)
*D*_f_: molecular diffusivity of the solute (m^2^ s^–1^)
*D*_n_: dispersion number
*D*_L_: axial dispersion coefficient (m^2^ s^–1^)
*D*: particle diameter (m)
*k*: cake permeability (m^2^)
*L*: cake height (m)
*M*: mass of particle (kg)
*Q*: volumetric flow rate (m^3^ s^–1^)
*u*_s_: superficial velocity of wash (m s^–1^)
*V*_cake_: total volume of cake (m^3^)
*V*_s_: volume of solids (m^3^)
*V*_v_: volume of voids (m^3^)
*V*_w_: volume of wash (m^3^)
wash ratio (m^3^ m^–3^)
*x*_sv_: particle volume equivalent diameter (m)
*Sc*: Schmidt number
*S*_0_: specific surface area (m^2^)
Δ*P*: pressure drop along the filter axis (kg m^–1^ s^–2^)
μ: filtrate viscosity (kg m^–1^ s^–1^)
*Re*: Reynold number
*R*_m_: filter medium resistance (m^–1^)
*t*: time (s)
*u*: superficial velocity of fluid (m s^–1^)
*u*_w_: superficial velocity of the wash liquid (m s^–1^)
*v*_w_ = *L*ε_av_: cumulative wash liquid passing through the cake (m^3^ m^–2^)
*V*: filtrate volume removed (m^3^)

## Greek Letters

α_av_: average specific cake resistance (m kg^–1^)
ε: cake porosity
ε_av_: cake porosity average
ρ: density (kg m^–3^)
ρ_s_: density of solids (kg m^–3^)
ρ_l_: density filtrate (kg m^–3^)
δ: thickness of the dissolution layer (m)

## Subscripts and Superscripts

e: exit of filter cake
*i*: initial
*j*: species *j*
w: inlet wash stream
