# Supplementary material for: Digital Design of Filtration and Washing of Active Pharmaceutical Ingredients via Mechanistic Modeling
Source: Org Process Res Dev. 2022 Dec 6;26(12):3236–53. doi: 10.1021/acs.oprd.2c00165 (PMC9764418; doi:10.1021/acs.oprd.2c00165)
Supplement: Supplementary file 1 — op2c00165_si_001.pdf [file op2c00165_si_001.pdf]

Supplementary information

Digital design of filtration and washing of active pharmaceutical ingredients via mechanistic modelling

Sara Ottoboni[1,2], Cameron J. Brown[1,3], Bhavik Mehta[4], Guillermo Jimeno[4], Niall A. Mitchell[4], Jan Sefcik[1,2], Chris J. Price[1,2]*

1 EPSRC Future Manufacturing Hub in Continuous Manufacturing and Advanced Crystallisation, University of Strathclyde, Glasgow, G1 1RD, UK

2 Department of Chemical and Process Engineering, University of Strathclyde, Glasgow, G1 1XJ, UK

3 Strathclyde Institute of Pharmacy & Biomedical Science (SIPBS), University of Strathclyde, Glasgow, G4 0RE, UK

4 Siemens Process Systems Engineering Ltd., London, W6 7HA, UK

Table S1 Initial conditions and material properties of the paracetamol case simulations.

Initial conditions	Paracetamol-water	Paracetamol-heptane	Paracetamol-dodecane	Paracetamol-acetonitrile
System information				
Component 1	Isopropanol	Isopropanol	Isopropanol	Isopropanol

Component 2	Paracetamol	Paracetamol	Paracetamol	Paracetamol
Component 3	Water	Heptane	Dodecane	Acetonitrile
Crystal phase	Paracetamol	Paracetamol	Paracetamol	Paracetamol
Main solute	Paracetamol	Paracetamol	Paracetamol	Paracetamol
Liquid properties				
Molecular weight compound 1 (kg/mol)	6E-02	6E-02	6E-02	6E-02
Molecular weight compound 2 (kg/mol)	1.51E-01	1.51E-01	1.51E-01	1.51E-01
Molecular weight compound 3 (kg/mol)	1.8E-02	1.00E-01	1.70E-01	4.10E-02
Mass density coefficient component 1 (kg/m^3)	786	786	786	786
Mass density coefficient component 2 (kg/m^3)	1260	1260	1260	1260
Mass density coefficient component 3 (kg/m^3)	997	684	750	786
Mass specific heat coefficient component 1 (J/molK)	1000	1000	1000	1000
Mass specific heat coefficient component 2 (J/molK)	1000	1000	1000	1000
Mass specific heat coefficient component 3 (J/molK)	1000	210	376	92
Dynamic viscosity coefficient component 1 (Pas)	1.93E-03	1.93E-03	1.93E-03	1.93E-03
Crystal properties				
Crystal stoichiometry	Isopropanol=0 Paracetamol=1 Wash solvent=0	Isopropanol=0 Paracetamol=1 Wash solvent=0	Isopropanol=0 Paracetamol=1 Wash solvent=0	Isopropanol=0 Paracetamol=1 Wash solvent=0

Mass density coefficient (kg/m^3)	1260	1260	1260	1260
Mass specific heat coefficient (J/molK)	1000	1000	1000	1000
Enthalpy of crystallisation (J/kg)	0	0	0	0
Volumetric shape factor	0.5236	0.5236	0.5236	0.5236
Solubility				
Key component	Isopropanol	Isopropanol	Isopropanol	Isopropanol
Solubility coefficient	0.1168 for case 1 Solubility binary plot polynomial coefficient for case 2	0.1168 for case 1 Solubility binary plot polynomial coefficient for case 2	0.1168 for case 1 Solubility binary plot polynomial coefficient for case 2	0.1168 for case 1 Solubility binary plot polynomial coefficient for case 2
Grid parameters				
Number of grid	50	50	50	50
Grid type	Logarithmic	Logarithmic	Logarithmic	Logarithmic
Min particle size distribution (µm)	1	1	1	1
Max particle size distribution (µm)	2000	2000	2000	2000

Table S2 Initial conditions and material properties of the mefenamic acid case simulations.

**Initial conditions**	**Mefenamic acid-heptane**
System information	
Component 1	2-butanol
Component 2	Mefenamic acid
Component 3	Heptane
Crystal phase	Mefenamic acid
Main solute	Mefenamic acid
Liquid properties	
Molecular weight compound 1 (kg/mol)	7.41E-02
Molecular weight compound 2 (kg/mol)	2.41E-01
Molecular weight compound 3 (kg/mol)	1.00E-01
Mass density coefficient component 1 (kg/m^3)	806
Mass density coefficient component 2 (kg/m^3)	1203
Mass density coefficient component 3 (kg/m^3)	684
Mass specific heat coefficient component 1 (J/molK)	199

Mass specific heat coefficient component 2 (J/molK)	2000
Mass specific heat coefficient component 3 (J/molK)	210
Dynamic viscosity coefficient component 1 (Pas)	3E-03
Crystal properties	
Crystal stoichiometry	2-butanol=0<br>Mefenamic acid=1<br>Heptane=0
Mass density coefficient (kg/m^3)	1203
Mass specific heat coefficient (J/molK)	1000
Enthalpy of crystallisation (J/kg)	0
Volumetric shape factor	0.5925
Solubility	
Key component	2-butanol
Solubility coefficient	0.00948 for case 1<br>Solubility binary plot polynomial coefficient for case 2
Grid parameters	
Number of grid	100
Grid type	Logarithmic
Min particle size distribution (µm)	0.1
Max particle size distribution (µm)	2000

Table S3 Parameters used as initial condition for the washing process done after filtration stopped to dryland.

**Parameters (initial condition)**	**Unit mass**	**Paracetamol case**	**Mefenamic acid case**
Void fraction	-	0.44	0.3916
Filter media diameter	m	0.027	0.027
Solid mass	-	5.895	4.43
Solid density	kg/m^3	1260	1203
Liquid composition, crystallisation solvent mass fraction	-	0.88	0.93
Liquid composition, API mass fraction	-	0.12	0.07
Liquid composition, wash solvent mass fraction	-	0	0
Crystallisation solvent density	kg/m^3	786 (isopropanol)	806 (2-butanol)
Wash solvent density	kg/m^3	Depend on the solvent selected	684 (heptane)

		(see supporting information)	

Filtration model

The liquid source is used to feed the washing solvent into the reactor at 25°C with a flow rate fixed at 50g/min. The liquid sink is used to collect the filtrate removed during washing and the liquid sensor is used to monitor the filtrate mass fraction composition during the washing process. The crystalliser model conditions used are the following:

- No nucleation, reaction, crystal growth, dissolution, or agglomeration are considered during the washing process.
- The initial liquid volume estimated in the cake is equivalent to the cake void volume and it was calculated during the filtration simulation. Two cases are considered: cake saturated with mother liquor at the end of the filtration (dryland) and partial deliquored cake (breakthrough).
- Washing process temperature is set to 25°C.
- Initial liquid phase composition is the same used for filtration process, as well for the initial solid properties.

This model considers three different scenarios:

- Cake filtered with partial deliquoring (breakthrough) where the wash solvent is fed into the washing chamber but no outflow is considered.
- Cake filtered maintaining the packed bed saturated with mother liquor (dryland) where the wash solvent feed flow rate and the filtrate removal flow rate are matching.
- Cake filtered keeping the packed bed saturated with mother liquor (dryland) where the wash solvent is fed into the washing chamber but no outflow is considered.

For the cases where the system does not consider outflow, the liquid sink considers an outflow stream flow rate of 0g/min. For the case where the inlet and outlet stream are matching the outlet flow stream was set to a flow rate matching the liquid source stream.

Model 1c: diffusion-dispersion washing
Main channels are defined as the channel created by pressure difference across the filter cake or created by excess liquid in the filter cake that is pushed out by the first passage of wash solvent. A general assumption is that in laminar flow conditions, wash solvent follows broadly straight channels along the main direction of wash flow without axial mixing, therefore no side channel mixing happens.

Model 1c-PF was first designed as a plug flow crystalliser with length equivalent to the cake height simulated from the filtration model, reactor diameter equivalent to the filtration diameter (same as the Biotage filtration unit). The crystalliser was considered as a system with an inaccessible volume fraction equal to the cake solid fraction (solidosity). The plug flow crystalliser is connected to a liquid source, a liquid sink, and a liquid composition sensor. The liquid source is used to feed into the crystalliser the wash solvent at 25°C with a flow rate fixed at 50g/min. The liquid sink is used to collect the filtrate removed during washing and the liquid sensor is used to monitor the filtrate mass fraction composition during the washing process. The reactor model conditions used are the followings:

- The plug flow reactor is simulated as a single tube with 10 grids.
- No nucleation, reaction, crystal growth or dissolution, and agglomeration are considered during the washing process.
- The axial dispersion coefficient is calculated as reported by Huhtanen et al. (2012), and Tien (2012) [30, 52], considering the filtrate flow velocity calculated from Darcy's equation, the molecular Peclet number, the Reynolds' number, and the Schmidt number. The axial

dispersion coefficient also considers the tortuosity of the cake calculated assuming a cake formed by 2D random overlapping mono-sized squared particles, with cake porosity in the range of 0.4 and 0.9 [66-68]. Solute molecular diffusivity coefficient for the wash estimated in the same order as solute-liquid diffusivity coefficient (1E-09m2/s, as reported by [57]). Cake porosity and media resistance were empirically estimated from similar experiments [68-69]. Cake resistance and cake height were simulated from the filtration model considering a filtration driving force of 500mbar (35.5bar/m). The axial dispersion coefficient for the different simulated cases were calculated using the equation related to molecular Peclet number higher than 4 and the Reynolds number less than 10. For further information about the equation used to calculate this coefficient, please refer to Tien (2012) [30].

- The initial liquid volume estimated in the cake is equivalent to the cake void volume and it was calculated during the filtration simulation.
- Initial liquid phase composition is the same used for filtration process, as well for the initial solid properties.

Model 1c-CSTR was implemented as a cascade of 10 well-mixed crystallisers with dimension equivalent to the cake volume predicted in the filtration model (mL) where a liquid source is connected to the first crystalliser, and for each crystalliser a liquid composition sensor is connected. A liquid sink is connected to the 10th reactor. The liquid source is used to feed into the first crystalliser the wash solvent at 25°C with a flow rate fixed at 50g/min. The liquid sink is used to collect the filtrate removed during washing and the liquid sensor is used to monitor the filtrate mass fraction composition during the washing process. The crystalliser model conditions used are the followings:

- Each crystalliser is simulating a liquid and solid outflow where the crystallisers' relative fill is set to 10% of the total volume and the time constant of flow response is set to 1E+04g/min. The time constant represents how quickly the flow out changes with regards to changes in hold up volume. The value selected allows to simulate a quasi-instantaneous liquid tank filling and to be able to transfer part of the liquid in the following tank without having a sharp change in the differential equation.
- No nucleation, reaction, crystal growth or dissolution, and agglomeration are considered during the washing process.
- The initial condition of the liquid phase considers a relative fill of the crystalliser of the 10% where the fraction hold up is pure liquid phase. The liquid phase composition is equivalent to the mother liquor composition.

Comparing the two approaches used to simulate diffusion-dispersion washing it is observable that the two approaches are giving the same initial and end point of solute concentration, while a slightly different profile of the intermediate regime is obtained. To get comparable intermediate regime, that is mainly governed by dispersion mechanism, more reactors can be used in series to better simulate the solute dispersion effect in the liquid phase.

Model 2c: diffusion-dispersion washing with dissolution

The first reactor is connected to a liquid source that provides the injection of wash solvent, while the last one is connected to a liquid sink to remove the filtrate. The liquid source is used to feed into the reactor the wash solvent at 25°C with a flow rate fixed at 50g/min. A liquid composition sensor and a particle size distribution sensor is connected for each reactor to monitor the evolution of the liquid phase and the evolution of the solid phase dissolution during washing. As for model

2b a recycle loop was connected to the outlet flow of each reactor to force the system to maintain in the reactor the solid phase of the initial suspension.

The reactor model conditions used are the followings:

- As for model 1b, each reactor is simulating a liquid and solid outflow where the reactors' relative fill is set to 10% of the total volume and the time constant of flow response is set to 1E+04g/min. The time constant represents how quickly the flow out changes with regards to changes in hold up volume. The value selected allows to simulate a quasi-instantaneous liquid tank filling and to be able to transfer part of the liquid in the following tank without having a sharp change in the differential equation.
- No nucleation, reaction, and agglomeration are considered during the process.
- An empirical power law growth/dissolution expression is used to model solid phase dissolution [70]; this expression assumes that the supersaturation of the system is described by an absolute value of 1. Considering a supersaturation of 1 means that the system is in thermodynamically in equilibrium. The solubility used is assumed non-size dependent. Activation energy of the growth/dissolution process is set to zero. As for model 2b, the growth/dissolution rate constant is chosen to allow the system state of equilibrium throughout the entire washing process. For each solvents combination simulated in this work a selected value of growth/dissolution rate constant was used to get the evolution of the solute concentration matching the concentration of the solute in equilibrium condition.
- The initial liquid volume estimated in the cake is equivalent to the cake void volume and it was calculated during the filtration simulation. The liquid and solid outflow conditions set are 0.1 of relative refill with time constant of flow response of 500g/min.

- The initial condition of the liquid phase considers a relative fill of the reactor of the 10% where the fraction hold up is pure liquid phase. The liquid phase composition is equivalent to the mother liquor composition.
- The initial solid phase conditions consider a cake formed of solid particles with a single peak lognormal density distribution function PSD with a specified mean size and standard deviation parameter. These two values derive from the raw material particle size analysis measurement.

Solubility

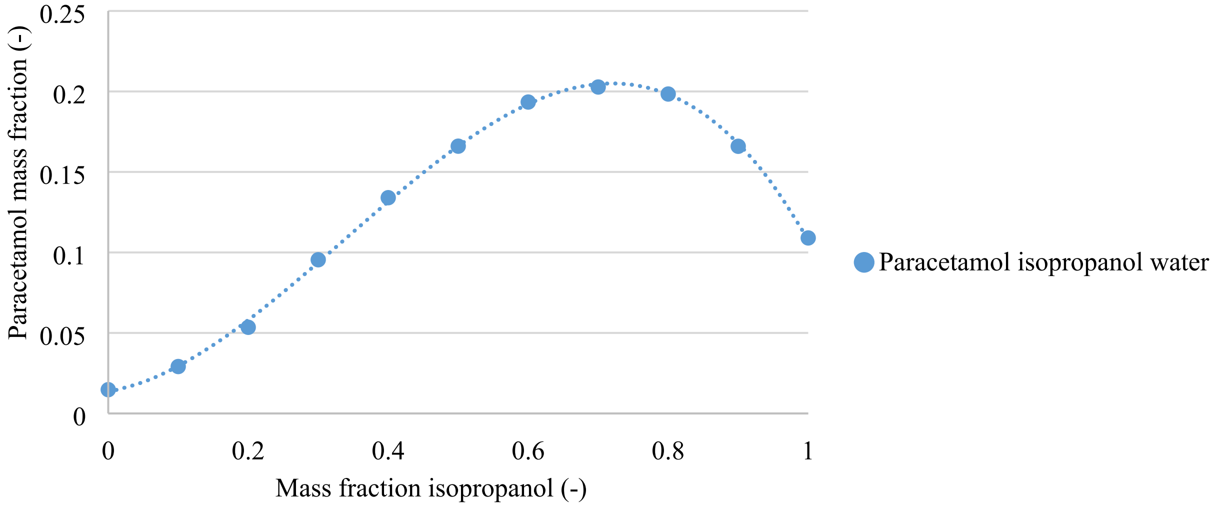

Figure S1 Binary plot of solubility of paracetamol in the binary mixture isopropanol-water. On the y-axis the mass fraction of the paracetamol is plotted, while on the x-axis the mass fraction of isopropanol is reported. The polynomial fitting curve with isopropanol as key component is used for the gPROMS case 2 models.

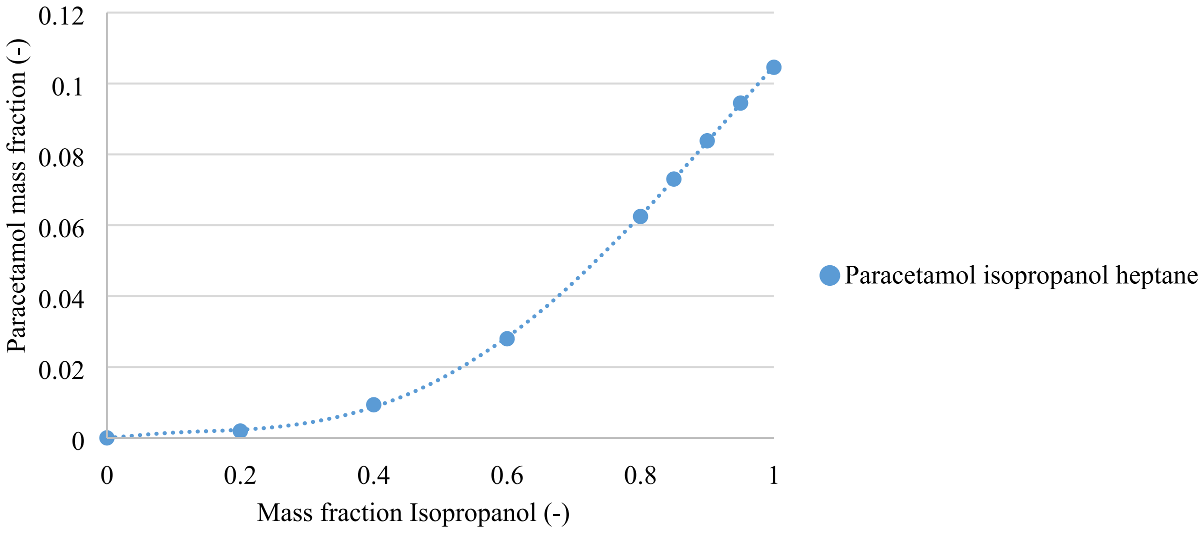

Figure S2 Binary plot of solubility of paracetamol in the binary mixture isopropanol-heptane. On the y-axis the mass fraction of the paracetamol is plotted, while on the x-axis the mass fraction of isopropanol is reported. The polynomial fitting curve with isopropanol as key component is used for the gPROMS case 2 models.

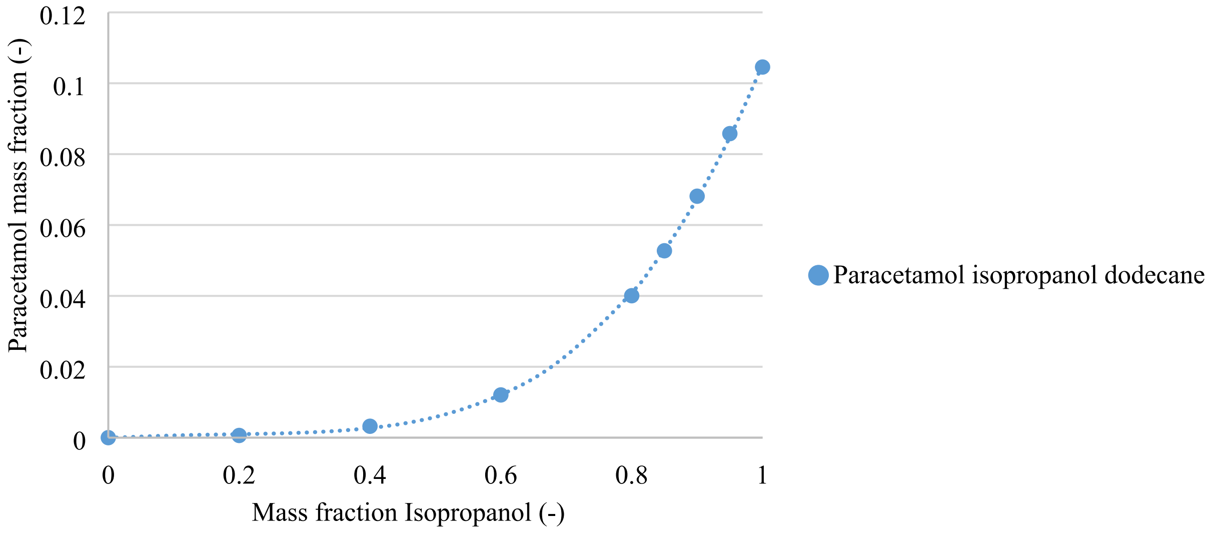

Figure S3 Binary plot of solubility of paracetamol in the binary mixture isopropanol-dodecane. On the y-axis the mass fraction of the paracetamol is plotted, while on the x-axis the mass fraction of isopropanol is reported. The polynomial fitting curve with isopropanol as key component is used for the gPROMS case 2 models.

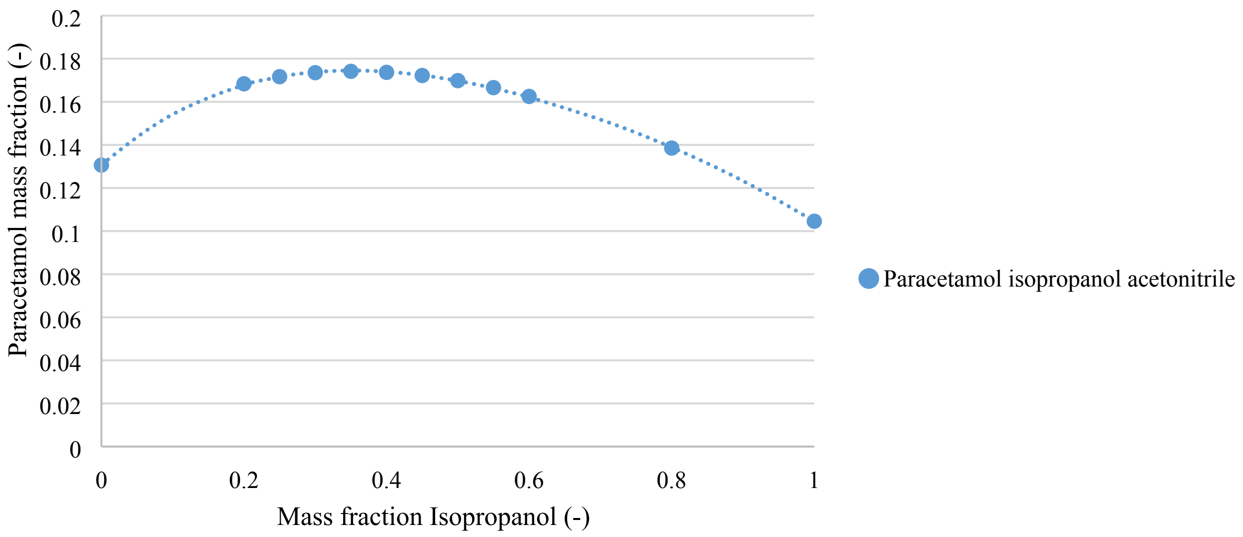

Figure S4 Binary plot of solubility of paracetamol in the binary mixture isopropanol-acetonitrile. On the y-axis the mass fraction of the paracetamol is plotted, while on the x-axis the mass fraction of isopropanol is reported. The polynomial fitting curve with isopropanol as key component is used for the gPROMS case 2 models.

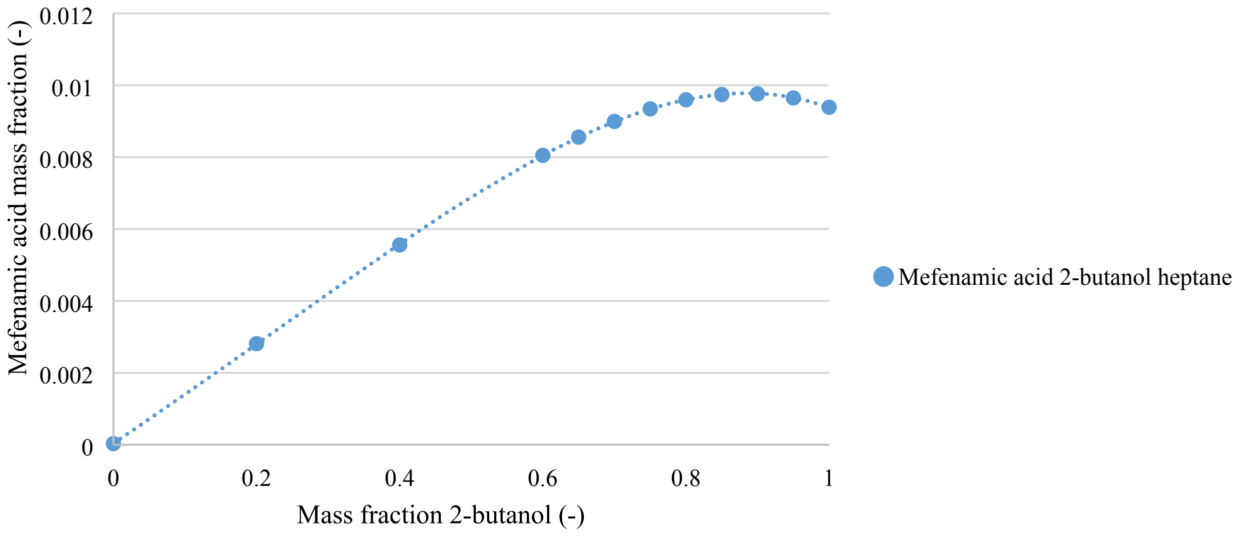

Figure S5 Binary plot of solubility of mefenamic acid in the binary mixture 2-butanol-heptane. On the y-axis the mass fraction of the mefenamic acid is plotted, while on the x-axis the mass fraction of 2-butanol is reported. The polynomial fitting curve with 2-butanol as key component is used for the gPROMS case 2 models.

Isolation performance modelling tools

System information conditions describe the components of the slurry/cake used, reporting in detail the composition of the solid and liquid phase. Liquid properties conditions consider the relevant

properties of the solvent and solute species constituting the solution part of the slurry, while crystal properties consider the key properties and composition of the solid phase. Since during filtration and washing no crystallisation process is considered, the enthalpy of crystallisation is fixed to zero. With the term volumetric shape factor the aspect ratio of the solid particle is defined. The test compound (paracetamol or mefenamic acid) solubility used for model case 1 is the solubility in the mother liquor at 25°C. For model case 2, instead, the cubic polynomial fitting parameters of the binary solubility plot are considered in terms of mass fraction of the mother liquor removed. With the grid parameters the size and size of the particles of the solid fraction of the slurry is defined. The particle size distribution is described as a discretized system of 50 grid (logarithmic scale) for paracetamol and a 100 grid (logarithmic scale) for mefenamic acid where the minimum and maximum particle size of the solid fraction are reported as 0.1-2000μm. A detailed list of the initial conditions used for the different system simulated. In general liquid and solid phase properties were gathered from literature [62], while the volumetric shape factor was empirically determined from particle size analysis (G3, Malvern), and the solubility were simulated using COSMO*therm* [63-64] or gathered from literature (paracetamol isopropanol-water case, [65]).

Filtration results

Table S4 Parameters used as initial condition for the filtration and washing processes simulated with gPROMS.

**Initial conditions**	**Unit measure**
System information	
Component	-
Crystal phase	-
Main solute	-
Liquid properties	
Molecular weight	kg/mol
Mass density coefficient	kg/m^3
Mass specific heat coefficient	J/molK
Dynamic viscosity coefficient	Pas

Crystal properties	
Crystal stoichiometry	-
Mass density coefficient	kg/m^3
Mass specific heat coefficient	J/molK
Enthalpy of crystallisation	J/kg
Volumetric shape factor	-
Solubility	
Key component	-
Solubility coefficient	-
Grid parameters	
Number of grid	-
Grid type	
Min particle size distribution	µm
Max particle size distribution	µm

Table S5 Filtration results for the paracetamol and mefenamic acid simulations.

	Paracetamol-Water		Paracetamol-Heptane	
	Dryland	Breakthrough	Dryland	Breakthrough
Filtration time (s)	15.274	16.921	15.639	17.554
Filtrate flow rate (m^3/s)	1.5960E-06	1.5413E-06	1.5940E-06	1.5416E-06
Cake resistance (m/kg)	9.2305E08	9.2305E08	9.2305E08	9.2305E08
Cake volume (m^3)	8.0362E-06	8.3216E-06	8.0463E-06	8.3201E-06
Volume liquid trapped (m^3)	3.5359E-06	3.6615E-06	3.5404E-6	3.6608E-06
Cake height (m)	0.0140	0.0145	0.0140	0.0145
	Paracetamol-Dodecane		Paracetamol-Acetonitrile	
	Dryland	Breakthrough	Dryland	Breakthrough
Filtration time (s)	15.376	17.292	15.39	17.306
Filtrate flow rate (m^3/s)	1.595E-06	1.5415E-06	1.595E-06	1.5415E-06
Cake resistance (m/kg)	9.2305E08	9.2305E08	9.2305E08	9.2305E08
Cake volume (m^3)	8.0411E-06	8.3209E-06	8.0415E-06	8.3209E-06
Volume liquid trapped (m^3)	3.5381E-06	3.6612E-06	3.5383E-06	3.6612E-06
Cake height (m)	0.01404	0.01453	0.01404	0.01453
	Mefenamic acid-Heptane			
	Dryland		Breakthrough	

Filtration time (s)	34.666	37.323
Filtrate flow rate (m^3/s)	7.4064E-07	7.2743E-07
Cake resistance (m/kg)	1.2762E09	1.2762E09
Cake volume (m^3)	7.2945E-06	7.4271E-06
Volume liquid trapped (m^3)	2.4933E-06	2.5386E-06
Cake height (m)	0.01274	0.01297

Pure displacement results

Displacement is defined as the replacement of the fluid, which fill the porous media by another fluid. To achieve perfect displacement, wash liquid has to penetrate in the entire cake pore without mixing with the original mother liquor of the initial suspension and plug flow must occur.

As represented by the wash curve simulated in Table S4 and Table S5, model 1a is a merely crude mass balance, where the amount of wash solvent entering produce the removal of the same quantity of mother liquor. During pure displacement washing first the mother liquor is removed completely from all the pores of the cake and replaced with clean wash solvent. As seen in Table S4 and Table S5 pure displacement washing model assume that the full removal of mother liquor is occurring by adding in the system only an equivalent cake volume of wash solvent, corresponding to 1 wash ratio. The quantity of solute dissolved in the mother liquor was assumed equal to the test compound solubility in the crystallization solvent, with a mother liquor supersaturation of 1. This assumption implied that the solute simulations using predicted solubility values with different wash solvents gave the same wash curve trend, in case the crystallization solvent used is the same (as for the paracetamol case). Another assumption of pure displacement washing is the non-miscibility between mother liquor and wash solvent. In model 1a it was also assumed that the solid phase is insoluble in the wash solvent, giving no residual paracetamol/mefenamic acid dissolved in wash solvent.

Table S6 Mass fraction concentration of the different species at different wash ratio simulated with model 1a for the paracetamol cases.

	Concentration at exit		
Wash ratio (Wr)	Isopropanol	Paracetamol	Water/Heptane/Acetonitrile/Dodecane
0	0.89	0.11	0
0.1	0.89	0.11	0
0.2	0.89	0.11	0
0.3	0.89	0.11	0
0.4	0.89	0.11	0
0.5	0.89	0.11	0
0.6	0.89	0.11	0
0.7	0.89	0.11	0
0.8	0.89	0.11	0
0.9	0.89	0.11	0
1	0.89	0.11	0
1	0	0	1
1.1	0	0	1
1.2	0	0	1
1.3	0	0	1
1.4	0	0	1
1.5	0	0	1
1.6	0	0	1
1.7	0	0	1
1.8	0	0	1
1.9	0	0	1
2	0	0	1

Table S7 Mass fraction concentration of the different species at different wash ratio simulated with model 1a for the mefenamic acid case.

	Concentration at exit		
Wash ratio (Wr)	2-butanol	Mefenamic acid	Heptane
0	0.98	0.02	0
0.1	0.98	0.02	0
0.2	0.98	0.02	0
0.3	0.98	0.02	0
0.4	0.98	0.02	0
0.5	0.98	0.02	0
0.6	0.98	0.02	0
0.7	0.98	0.02	0

0.8	0.98	0.02	0
0.9	0.98	0.02	0
1	0.98	0.02	0
1	0	0	1
1.1	0	0	1
1.2	0	0	1
1.3	0	0	1
1.4	0	0	1
1.5	0	0	1
1.6	0	0	1
1.7	0	0	1
1.8	0	0	1
1.9	0	0	1
2	0	0	1

Pure dilution results

Pure dilution model considers a process where the original cake liquid phase is diluted adding the wash solvent during the removal of the filtrate phase at a fixed injection flow rate of 50g/min. For the case of no outflow the outlet flow rate is set to 0g/min, while in case of inlet equal to the outlet flow rate the outlet flow is matching the feed flow rate. Since pure dilution model assumed that instant mixing occurs between the wash solvent and mother liquor, the driving force governing this process is the difference in density across the injected and ejected phases, in case outlet flow rate is different from 0g/min. For the two cases where no outlet flow is simulated, the governing mechanism reported is the simple dilution of the mother liquor. This model also assumed no solubility of the test compounds in the wash solvent, implying no dissolution of the solid phase. The simulated process provides the evolution of species concentration during dilution with wash solvent.

Three different cases where simulated (Figure S6a, Figure S7a, and Figure S8a). In the first case, in green, the cake filtered to breakthrough where the wash solvent is feed in the washing chamber but no outflow is considered. In the second case, in blue, the cake filtered keeping the packed bed

saturated with mother liquor (dryland) where the wash solvent feed flow rate and the filtrate removal flow rate are matching. The third case, in orange, the cake filtered keeping the packed bed saturated with mother liquor (dryland) where the wash solvent is feed in the washing chamber but no outflow is considered.

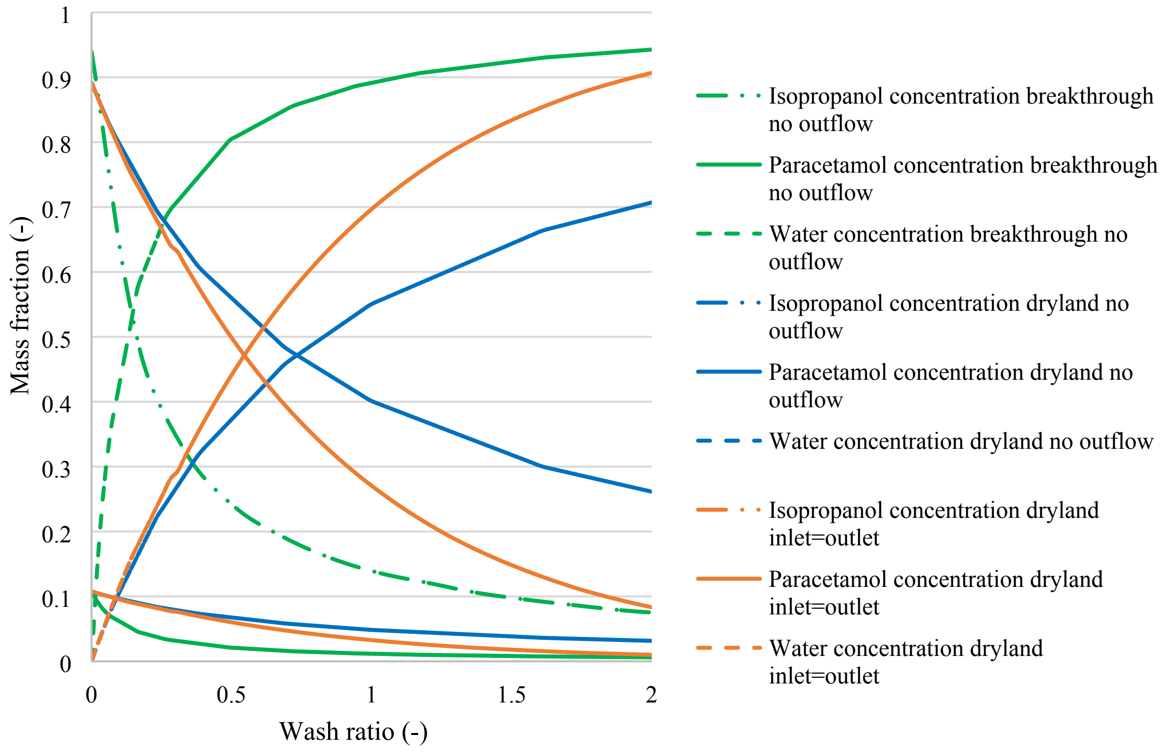

a

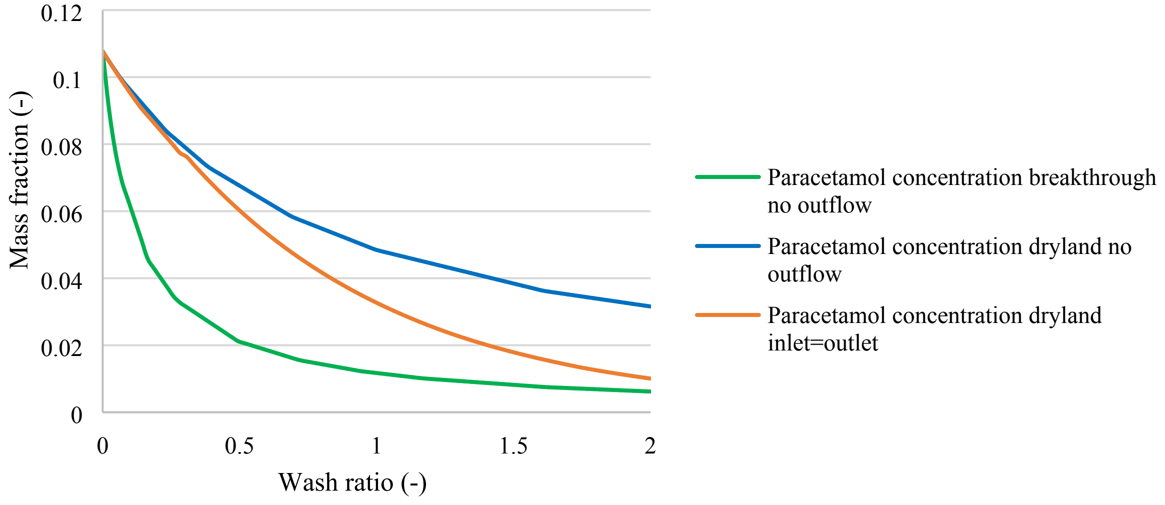

b

Figure S6 a) *Wash curve obtained from pure dilution model. Paracetamol was selected as test compound. Isopropanol was chosen as the mother liquor solvent and water was selected as wash solvent. b) Paracetamol solute phase mass evolution during washing for the three washing cases simulated.*

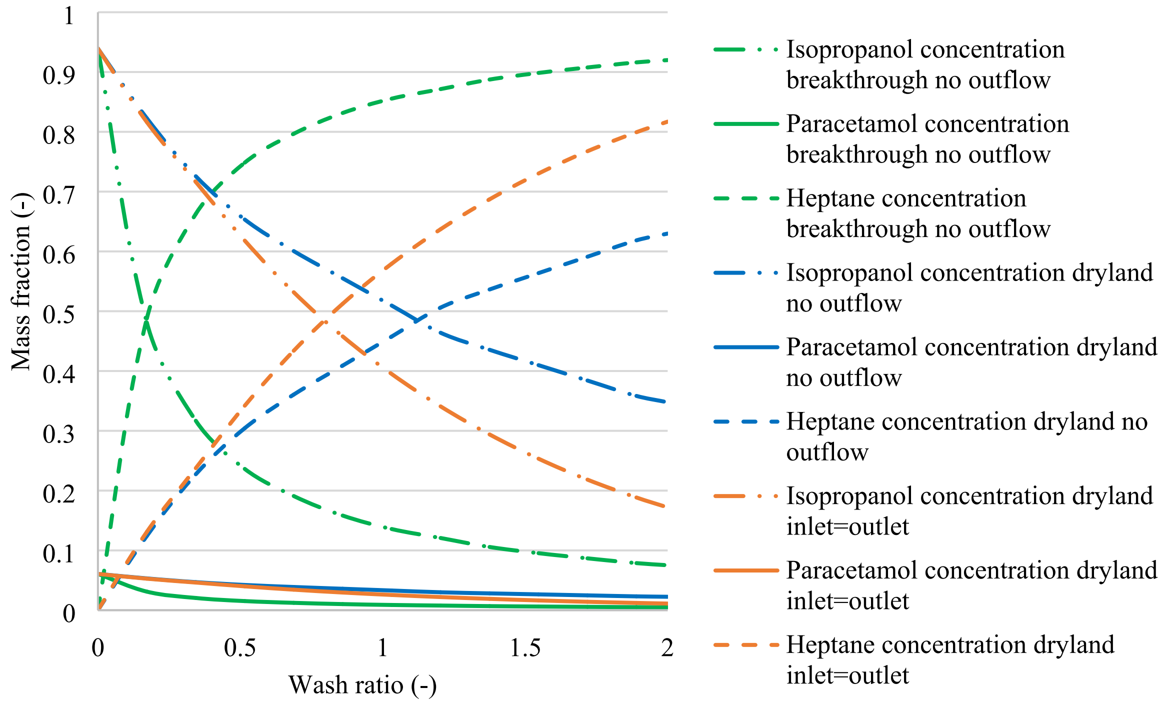

a

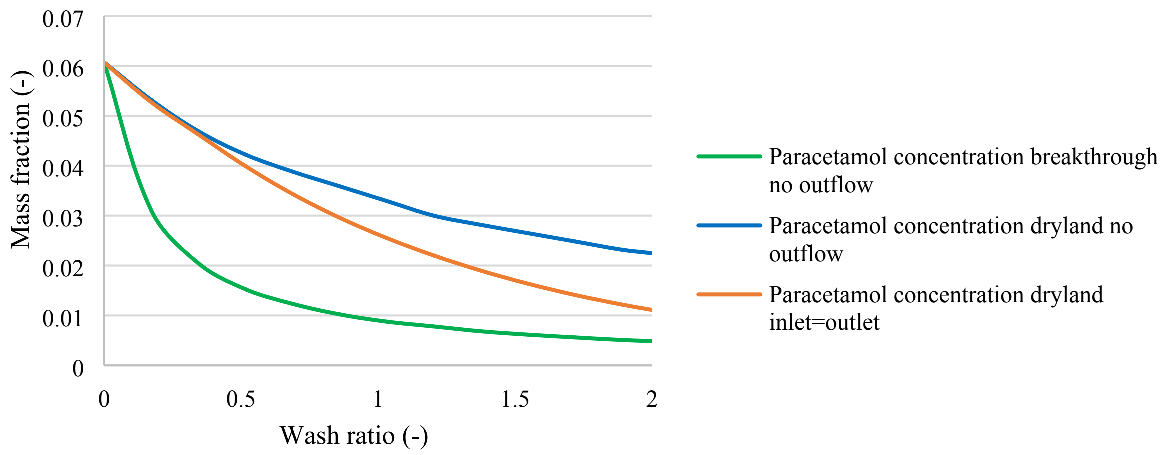

b

Figure S7 a) *Wash curve obtained from pure dilution model. Paracetamol was selected as test compound. Isopropanol was chosen as the mother liquor solvent and heptane was selected as wash solvent. b) Paracetamol solute phase mass evolution during washing for the three washing cases simulated.*

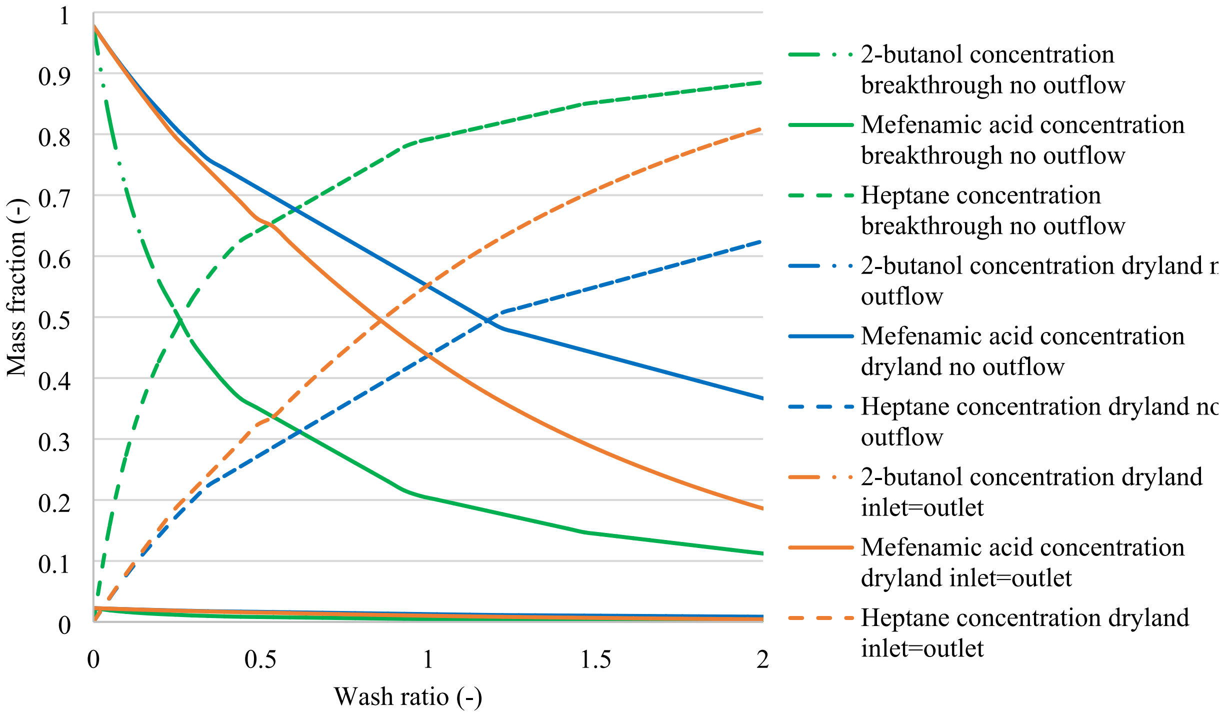

a

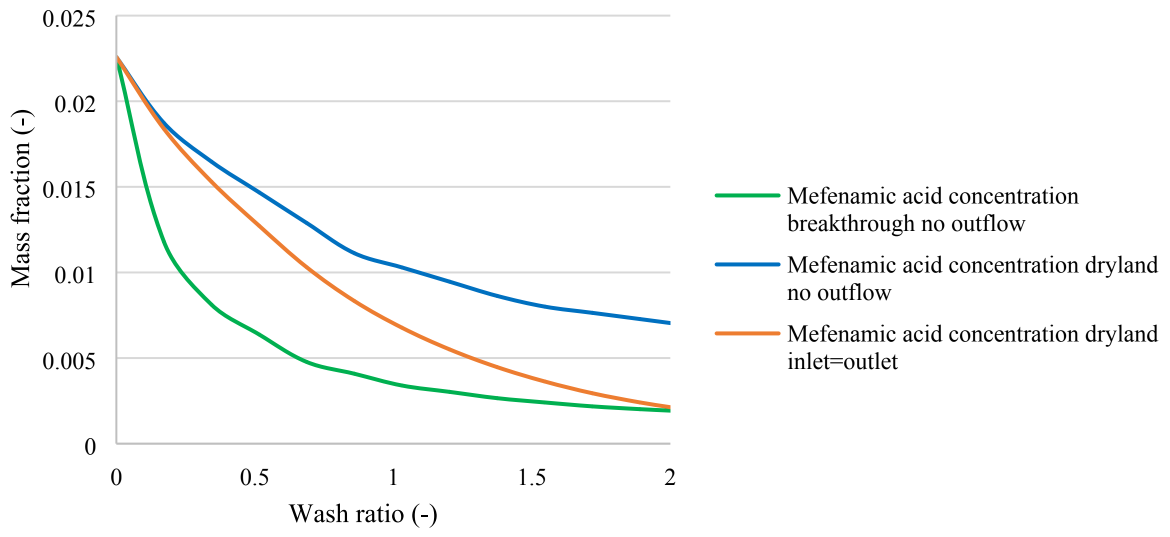

b

Figure S8 a) *Wash curve obtained from pure dilution model. Mefenamic acid was selected as test compound. 2-butanol was chosen as the mother liquor solvent and heptane was selected as wash solvent. b) Mefenamic acid solute phase mass evolution during washing for the three washing cases simulated.*

Analysing Figure S6 to Figure S8 the effect of filtration halting mechanism is clearly visible. In case filtration was stopped to breakthrough (green lines), a drastic drop in crystallisation solvent and solute is observed in the first part of the wash curves (Wr less than 0.5). The feed suspension in case of filtration stopped to breakthrough show a much smaller content of liquid phase (90% less) respect suspensions produced with a filtration process stopped to dryland (blue and orange curves), causing a much less amount (mass) of mother liquor to be removed during washing.

From Figure S6 to Figure S8 it is also observed that in case no outflow (green and blue lines), simple dilution is simulated, while in case the inlet and the outlet flow are matching further crystallisation solvent and solute concentration reduction is seen due to the removal of filtrate. From the solute phase concentration evolution graphs (Figure S6b, Figure S7b and Figure S8b) can be inferred that washing is more effective if filtration is stopped to breakthrough. However, from experiments reported from Ottoboni et *al.* (2018) [57], it was always recommended to stop

filtration to dryland to minimise wash solvent consumption (re-wetting of cake pores). Furthermore, the lack of diffusion mechanism in pure dilution model does not take in account back-mixing mechanism that reduce washing efficiency.

The simulated wash curve for paracetamol isopropanol-dodecane, and isopropanol-acetonitrile cases are below reported.

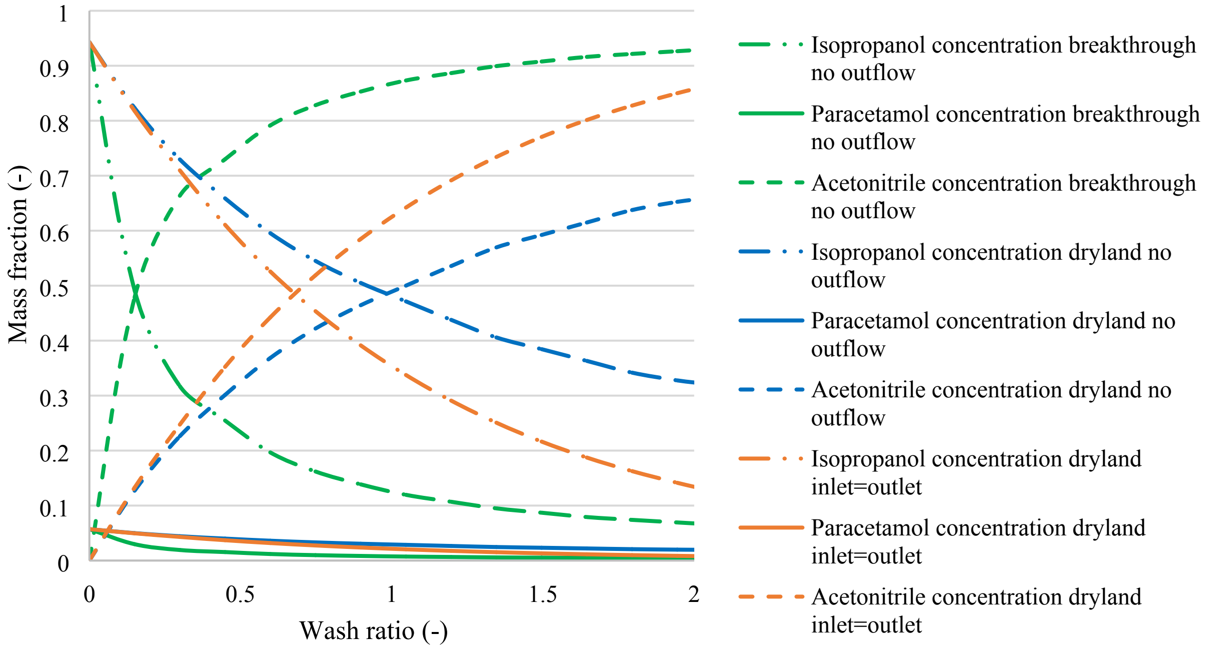

a

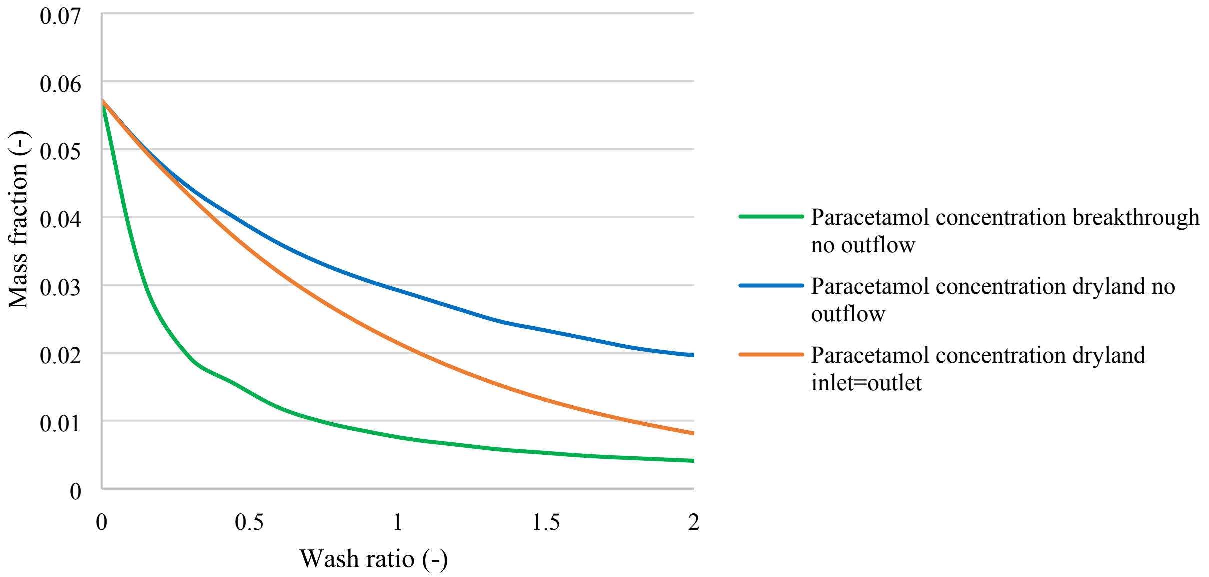

b

Figure S9 a) Mass fraction concentration of the different species at different wash ratio simulated with pure dilution model for the paracetamol case, where acetonitrile was selected as wash solvent. b) Paracetamol solute phase mass evolution during washing for the paracetamol case where acetonitrile was used as wash solvent.

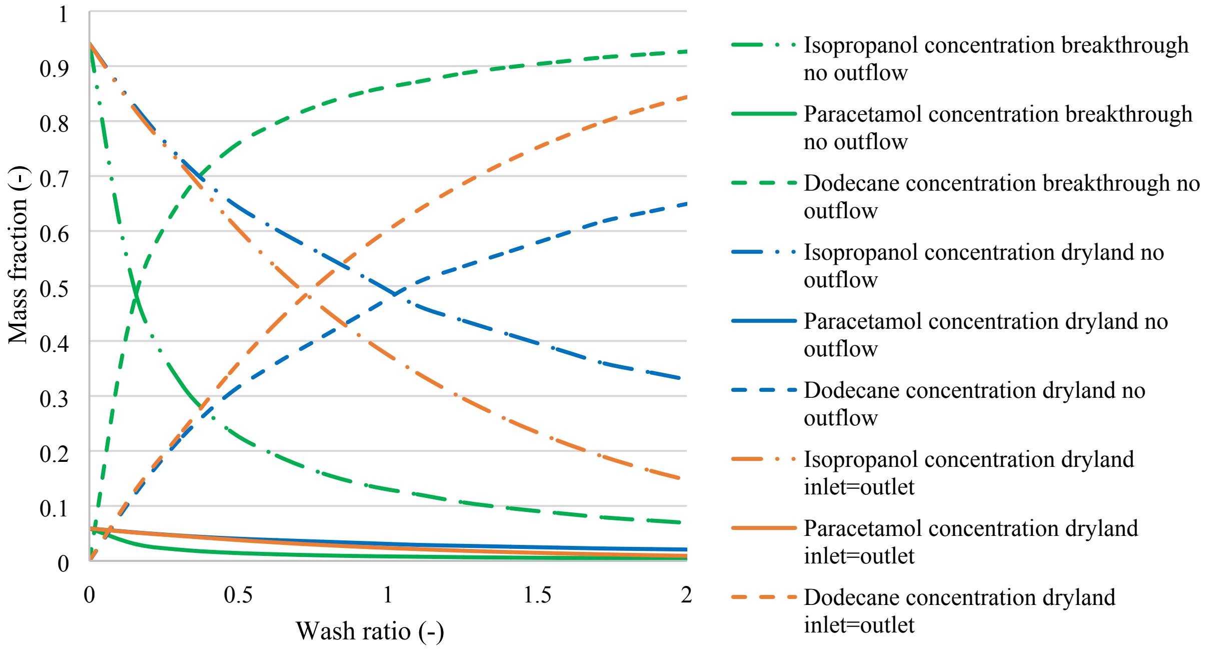

a

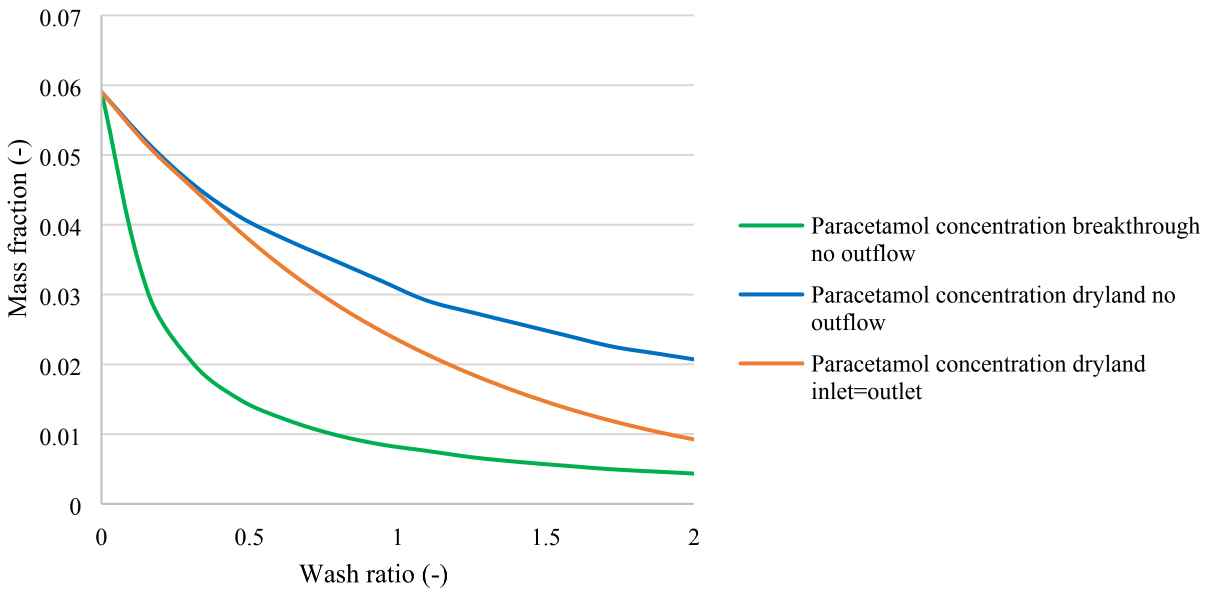

b

Figure S10 a) Mass fraction concentration of the different species at different wash ratio simulated with pure dilution model for the paracetamol case, where dodecane was selected as wash solvent. b) Paracetamol solute phase mass evolution during washing for the paracetamol case where dodecane was used as wash solvent.

Diffusion-dispersion results

Model 1c is combining the pure dilution with the axial dispersion washing mechanism in case the liquid phases mix instantaneously and in case the solubility of the test compound (paracetamol/mefenamic acid) is assumed zero in the wash solvent. Axial dispersion mechanism implementation in model 1c is described in section 3.2.3. As described for model 1a and model 1b, the initial cake, produced after simulated filtration process, is made by a sedimented packed bed of solid particles of the test compound. The cake the void volume, for model 1a, 1b dryland filtration, and 1c, is filled with mother liquor with solute concentration equal to the test compound solubility in the crystallisation solvent at 25°C, with supersaturation equal to 1.

As reported by Tien (2012) [30] the initial phase (flat part of the curve) of a washing curve is a result of direct hydrodynamic displacement of the residual mother liquor from the larger pores due to the wash liquid entering the cake. The second, intermediate stage occurs when there the mother liquor removal from the smaller pores in the cake; during this stage wash, solvent starts to disperse and dilute the filtrate from the larger pores in which a mass transfer process has started. In the third regime, the mass transfer stage, solute diffuses into the wash solvent this takes place over the entirety of the cake.

Two different washing models are used to simulate diffusion-dispersion washing (Figure S11 to Figure S13): using a plug flow setup (in blue), and using a CSTR approach (in green).

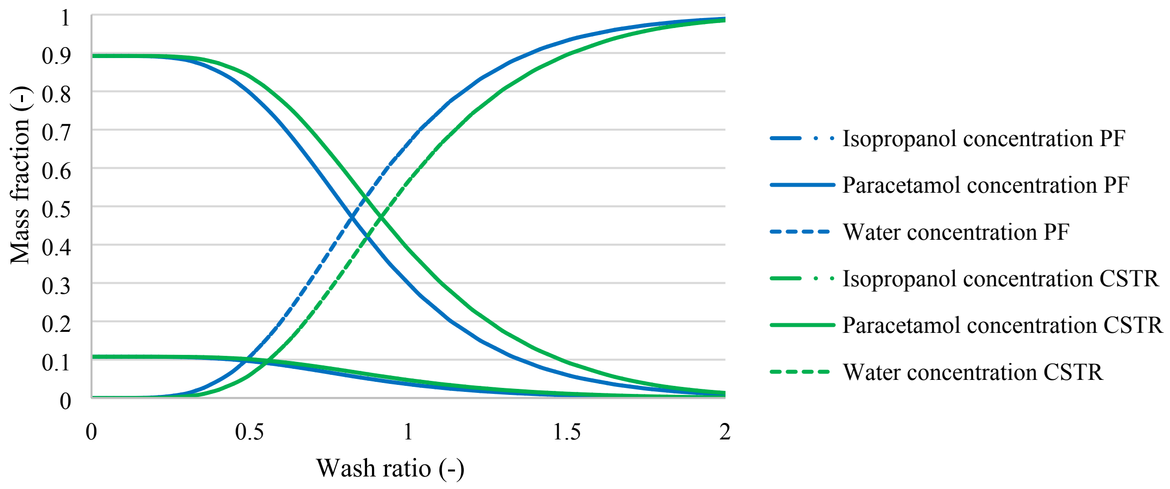

a

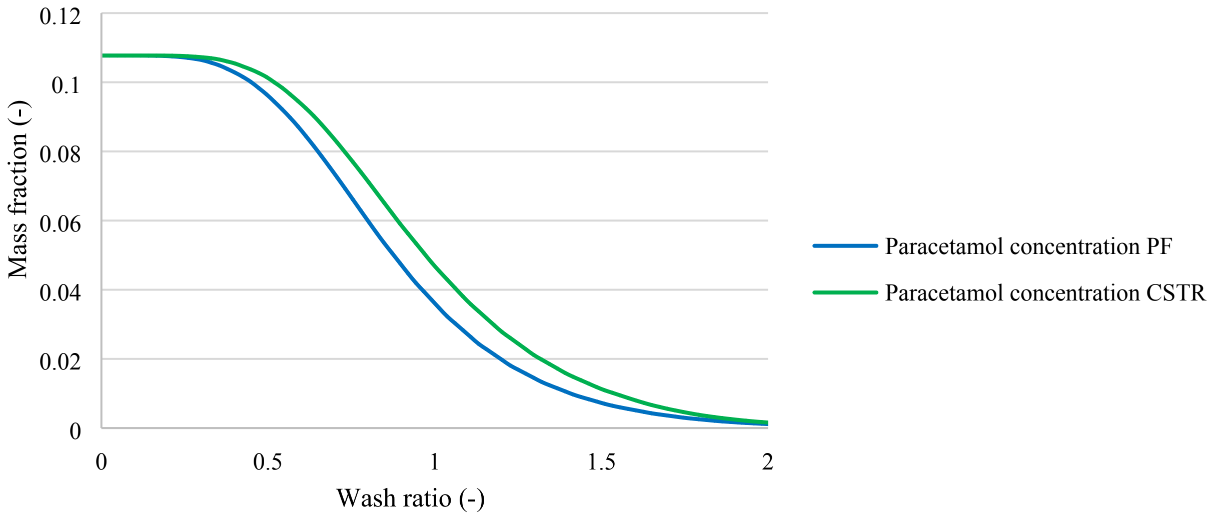

b

Figure S11 a) Wash curve obtained from diffusion-dispersion model. Paracetamol was selected as test compound. isopropanol was chosen as the mother liquor solvent. The wash solvent used was water. b) Paracetamol solute concentration at different wash ratio for the paracetamol case simulation with water as wash solvent.

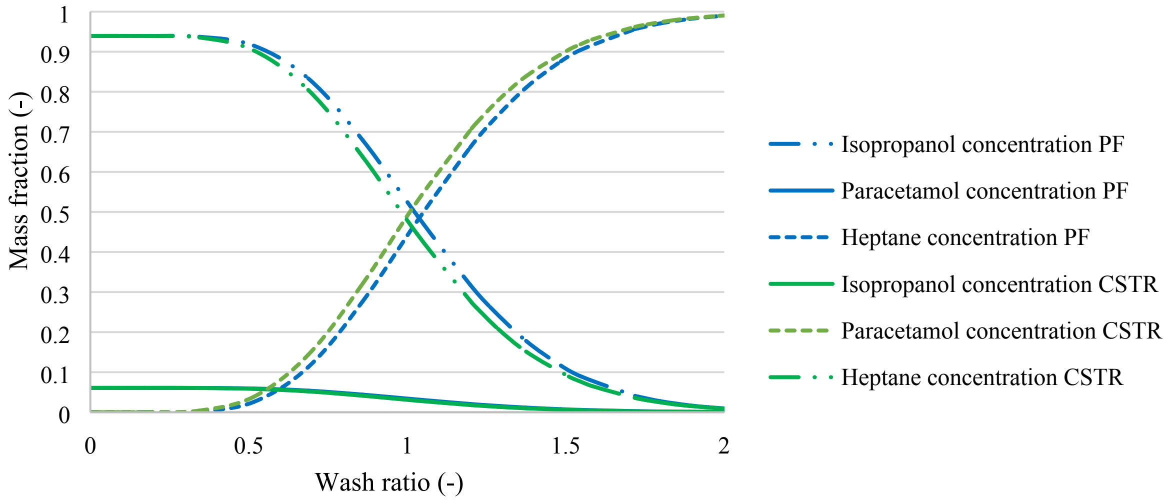

a

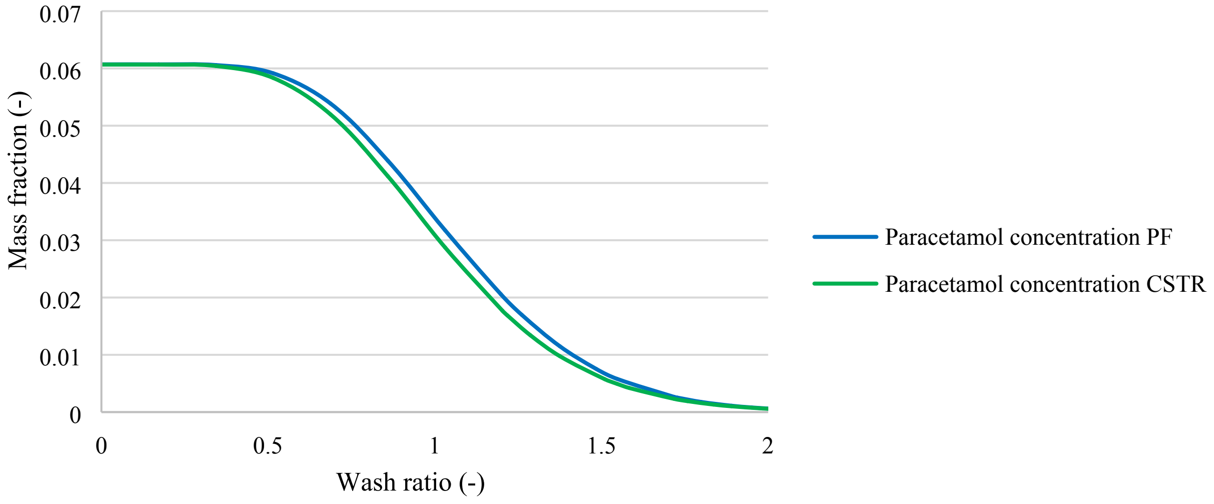

b

Figure S12 a) Wash curve obtained from diffusion-dispersion model. Paracetamol was selected as test compound. isopropanol was chosen as the mother liquor solvent. The wash solvent used was heptane. b) Paracetamol solute concentration at different wash ratio for the paracetamol case simulation with heptane as wash solvent.

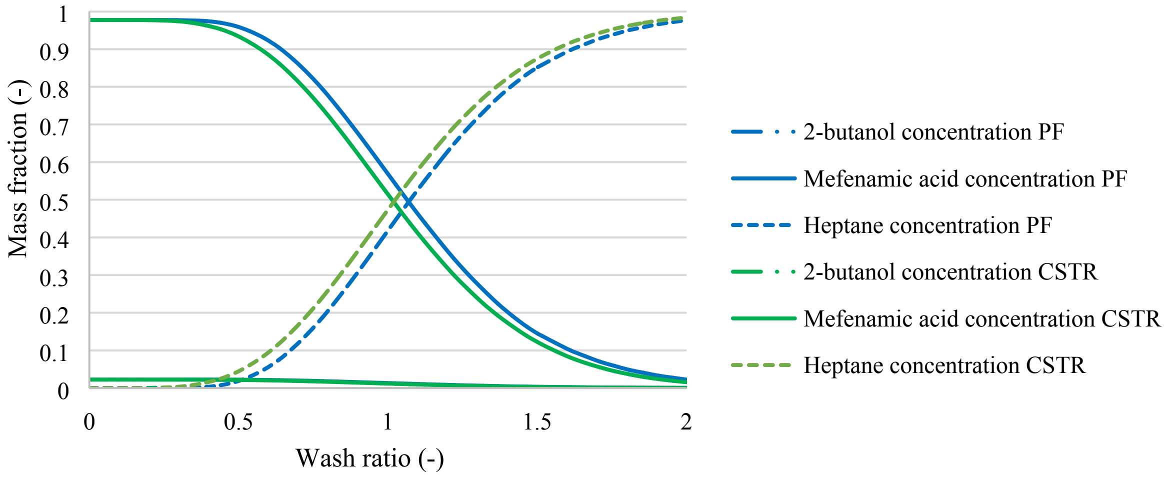

a

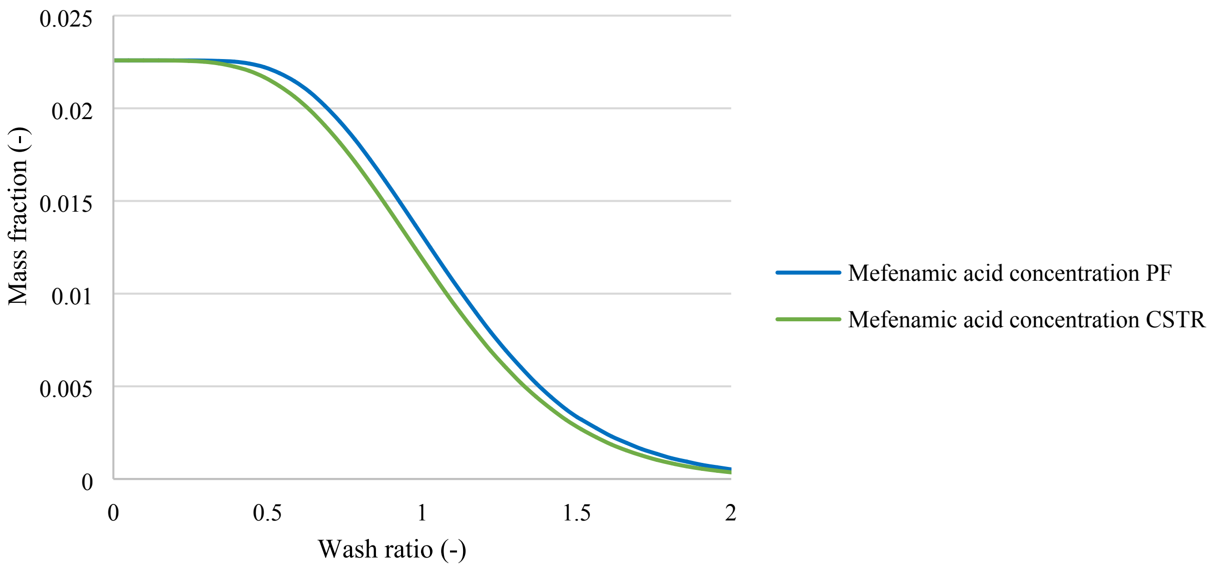

b

Figure S13 a) Mefenamic acid was selected as test compound; 2-butanol was chosen as the mother liquor solvent. The wash solvent used was heptane. b) Mefenamic acid solute concentration at different wash ratio for the mefenamic acid case simulation.

As reported from Figure S11 to Figure S13 the model provides detailed evolution of species concentration during washing. Paracetamol/mefenamic acid solute phase is reducing during washing due to dilution mechanism.

To simulate the washing curve of a washing process where the solid phase is made of non-soluble particles, the combination of 3 mechanisms (displacement, dilution-dispersion, and diffusion mechanisms) is required to get the characteristic shape of the wash curve with the three wash curve stages: constant rate, intermediate stage, and diffusion stage. A great improvement in wash curve shape simulation is seen in model 1c respect the previous two models (1a, and 1b). The simulated mother liquor wash curves (crystallisation solvent and solute phase) clearly show the intermediate and diffusion stage parts of the curves. Since this model is also considering the displacement mechanism of washing, the typical constant rate portion of the wash curve can be simulated with model 1c (initial flat part of the curve).

As reported in section 3.2.3, the two models used to simulate diffusion-dispersion washing produce the same initial and end point of solute concentration, while a slightly different profile of the intermediate regime is obtained. A future implementation of the CSTR model 1c is recommended by using a series of reactors to better simulate the solute dispersion effect in the liquid phase. In Figure S14, a comparison of the concentration of the different species for the PF and the CSTR models for the different simulation can be done.

The simulated wash curve for paracetamol isopropanol-dodecane, and isopropanol-acetonitrile cases are reported below.

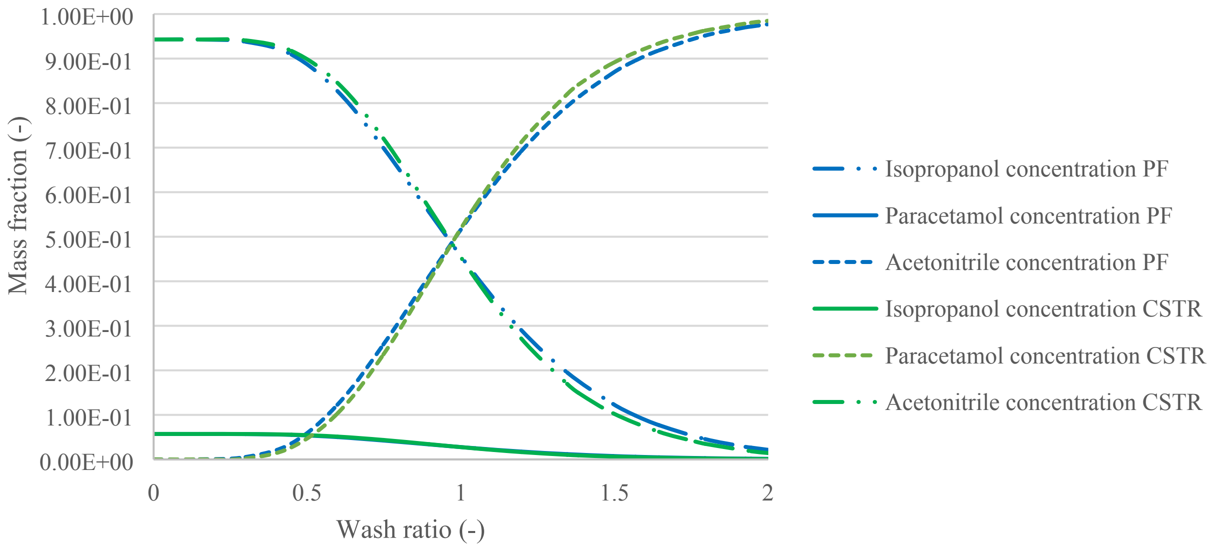

a

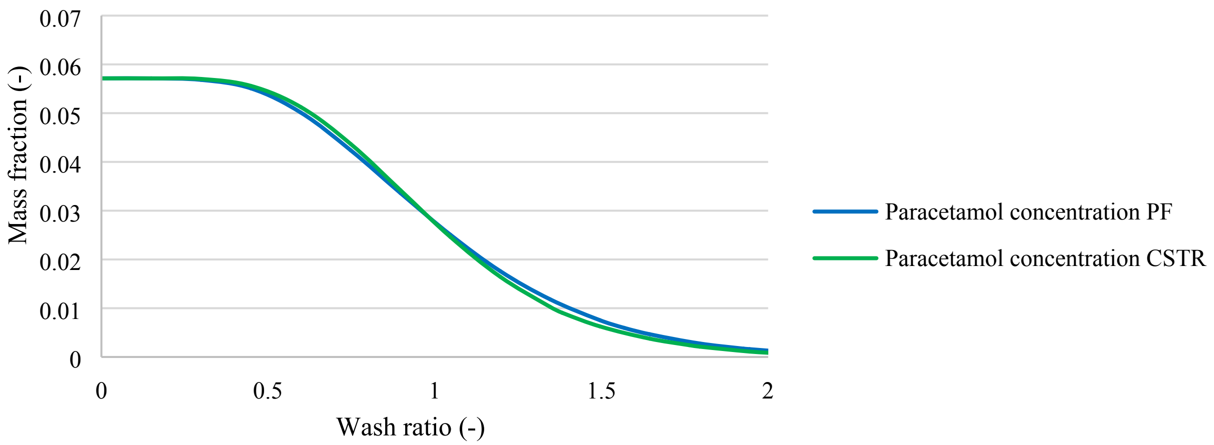

b

Figure S14 a) Mass fraction concentration of the different species at different wash ratio simulated with model 1c for the paracetamol case, where acetonitrile was selected as wash solvent. b) Paracetamol solute concentration at different wash ratio for the paracetamol case simulation with acetonitrile as wash solvent.

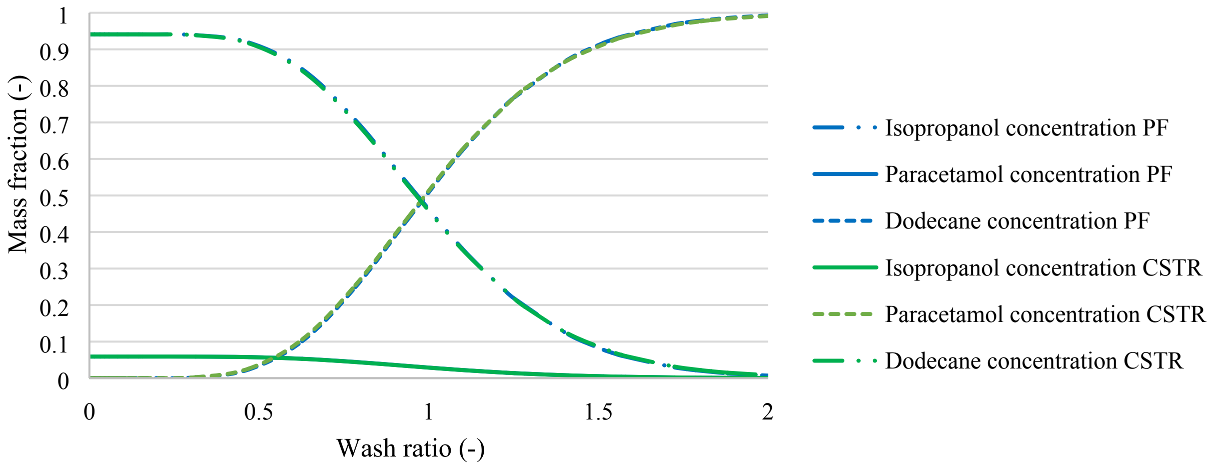

a

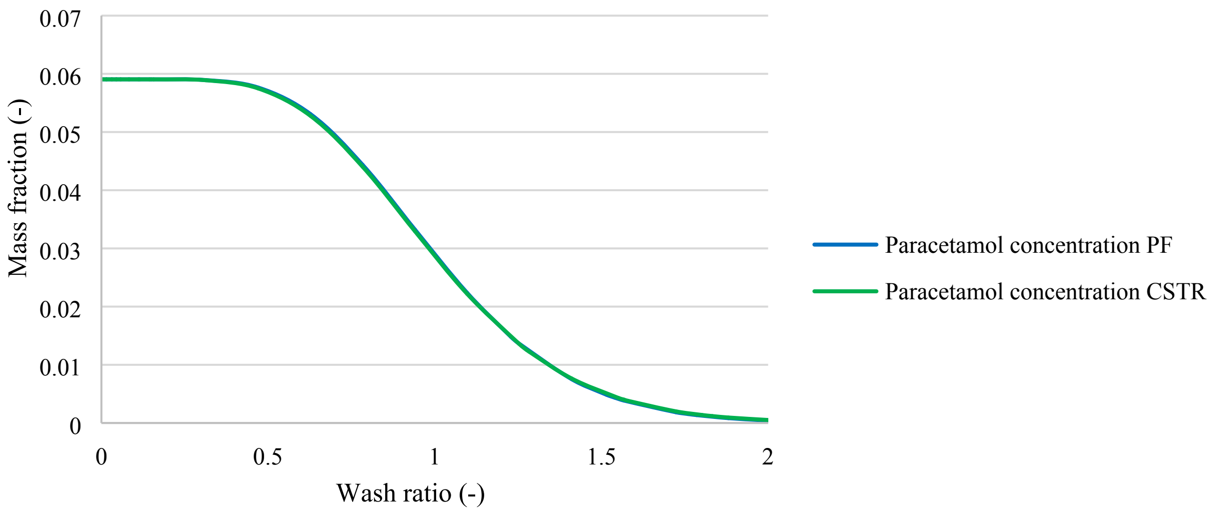

b

Figure S15 a) Mass fraction concentration of the different species at different wash ratio simulated with model 1c for the paracetamol case, where dodecane was selected as wash solvent. b) Paracetamol solute concentration at different wash ratio for the paracetamol case simulation with acetonitrile as wash solvent.

Model 2

Case 2 was design to simulate washing processes where the API is soluble in mother liquor and wash solvent, causing the risk of product loss by dissolution [70].

It is crucial for soluble cake to consider material dissolution during washing and to select accurately the dissolution thermodynamic and kinetic aspects. Wash solvent selection can also lead to dissolution of small particles and consequently narrowing of PSD and/or reducing isolation yield.

During washing the interaction between solid particles and solvent happens at the interface and solid/solvent interaction is determined as the sum of bonding forces between molecules, atoms and ions. Dissolution kinetics are also important: in the case of fast dissolution the wash liquor will be saturated by the product almost immediately after contact. On the other hand, when a system has

slow dissolution kinetics, the product loss due to dissolution of the solids can be reduced by having a short contact time between the solids and wash liquid.

In reality, when the solute is highly soluble in the wash solvent/mother liquor, a high case of product loss can be expected; in case of slow dissolution kinetics, reducing contact time between solvent and solute by modifying the pressure, can help to minimize product loss. However, sometimes particle surface dissolution can be useful for impurity removal [70].

If the surface during the washing step dissolves, the impurities remaining in stagnant interstitial areas, which are not accessible for the wash liquor can be set free as the areas are opened, so these impurities that usually are trapped in the particles structure can in this way easily be removed from the fine pores between crystals and the crystal surfaces.

Displacement with soluble wash solvent results

As represented by the wash curve simulated in Table S8 to Table S12, model 2a is similar to model 1a generating a merely crude mass balance, where the amount of wash solvent entering produce the removal of the same quantity of mother liquor. The only variation respect model 1a, is that in model 2a paracetamol/mefenamic acid are soluble in the wash solvent, causing partial dissolution of the particles. The quantity of solute dissolved in the mother liquor and in the wash solvent was assumed equal to the test compound solubility in the crystallization and wash solvent. As in model 1a, mother liquor and wash solvents are immiscible. The extent of particle dissolution was calculated uniformly across the entire cake volume. Particle size reduction for all the simulated cases is reported in Table S14.

Table S8 Mass fraction concentration of the different species at different wash ratio simulated with model 1a for the paracetamol case, where water was the wash solvent. Mean particle size after washing corresponded to 76.6μm, corresponding to a reduction of 0.4μm.

	Concentration at exit		
Wash ratio (Wr)	Isopropanol	Paracetamol	Water
0	0.89	0.11	0.00
0.1	0.89	0.11	0.00
0.2	0.89	0.11	0.00
0.3	0.89	0.11	0.00
0.4	0.89	0.11	0.00
0.5	0.89	0.11	0.00
0.6	0.89	0.11	0.00
0.7	0.89	0.11	0.00
0.8	0.89	0.11	0.00
0.9	0.89	0.11	0.00
1	0.89	0.11	0.00
1	0.00	0.01	0.99
1.1	0.00	0.01	0.99
1.2	0.00	0.01	0.99
1.3	0.00	0.01	0.99
1.4	0.00	0.01	0.99
1.5	0.00	0.01	0.99
1.6	0.00	0.01	0.99
1.7	0.00	0.01	0.99
1.8	0.00	0.01	0.99
1.9	0.00	0.01	0.99
2	0.00	0.01	0.99

Table S9 Mass fraction concentration of the different species at different wash ratio simulated with model 1a for the paracetamol case, where heptane was the wash solvent. Mean particle size after washing corresponded to 77µm.

	Concentration at exit		
Wash ratio (Wr)	Isopropanol	Paracetamol	Heptane
0	0.94	0.06	0.00
0.1	0.94	0.06	0.00
0.2	0.94	0.06	0.00
0.3	0.94	0.06	0.00
0.4	0.94	0.06	0.00
0.5	0.94	0.06	0.00
0.6	0.94	0.06	0.00
0.7	0.94	0.06	0.00
0.8	0.94	0.06	0.00
0.9	0.94	0.06	0.00
1	0.94	0.06	0.00
1	0.00	7.69E-07	1.00
1.1	0.00	7.69E-07	1.00
1.2	0.00	7.69E-07	1.00
1.3	0.00	7.69E-07	1.00
1.4	0.00	7.69E-07	1.00
1.5	0.00	7.69E-07	1.00
1.6	0.00	7.69E-07	1.00
1.7	0.00	7.69E-07	1.00
1.8	0.00	7.69E-07	1.00
1.9	0.00	7.69E-07	1.00
2	0.00	7.69E-07	1.00

Table S10 Mass fraction concentration of the different species at different wash ratio simulated with model 1a for the paracetamol case, where acetonitrile was the wash solvent. Mean particle size after washing corresponded to 75µm, corresponding to a reduction of 5µm.

	Concentration at exit		
Wash ratio (Wr)	Isopropanol	Paracetamol	Acetonitrile
0	0.94	0.06	0.00
0.1	0.94	0.06	0.00
0.2	0.94	0.06	0.00
0.3	0.94	0.06	0.00
0.4	0.94	0.06	0.00
0.5	0.94	0.06	0.00
0.6	0.94	0.06	0.00
0.7	0.94	0.06	0.00
0.8	0.94	0.06	0.00
0.9	0.94	0.06	0.00
1	0.94	0.06	0.00
1	0.00	0.06	0.94
1.1	0.00	0.06	0.94
1.2	0.00	0.06	0.94
1.3	0.00	0.06	0.94
1.4	0.00	0.06	0.94
1.5	0.00	0.06	0.94
1.6	0.00	0.06	0.94
1.7	0.00	0.06	0.94
1.8	0.00	0.06	0.94
1.9	0.00	0.06	0.94
2	0.00	0.06	0.94

Table S11 Mass fraction concentration of the different species at different wash ratio simulated with model 1a for the paracetamol case, where dodecane was the wash solvent. Mean particle size after washing corresponded to 77μm.

	Concentration at exit		
Wash ratio (Wr)	Isopropanol	Paracetamol	Dodecane
0	0.94	0.06	0.00
0.1	0.94	0.06	0.00
0.2	0.94	0.06	0.00
0.3	0.94	0.06	0.00
0.4	0.94	0.06	0.00
0.5	0.94	0.06	0.00
0.6	0.94	0.06	0.00
0.7	0.94	0.06	0.00
0.8	0.94	0.06	0.00
0.9	0.94	0.06	0.00
1	0.94	0.06	0.00
1	0.00	4.78E-07	1.00
1.1	0.00	4.78E-07	1.00
1.2	0.00	4.78E-07	1.00
1.3	0.00	4.78E-07	1.00
1.4	0.00	4.78E-07	1.00
1.5	0.00	4.78E-07	1.00
1.6	0.00	4.78E-07	1.00
1.7	0.00	4.78E-07	1.00
1.8	0.00	4.78E-07	1.00
1.9	0.00	4.78E-07	1.00
2	0.00	4.78E-07	1.00

Table S12 Mass fraction concentration of the different species at different wash ratio simulated with model 1a for the mefenamic acid case, where heptane was the wash solvent. Mean particle size after washing corresponded to 93µm.

	Concentration at exit		
Wash ratio (Wr)	2-butanol	Mefenamic acid	Heptane
0.0	0.98	0.02	0.00
0.1	0.98	0.02	0.00
0.2	0.98	0.02	0.00
0.3	0.98	0.02	0.00
0.4	0.98	0.02	0.00
0.5	0.98	0.02	0.00
0.6	0.98	0.02	0.00
0.7	0.98	0.02	0.00
0.8	0.98	0.02	0.00
0.9	0.98	0.02	0.00
1.0	0.98	0.02	0.00
1.0	0.00	1.47E-04	1.00
1.1	0.00	1.47E-04	1.00
1.2	0.00	1.47E-04	1.00
1.3	0.00	1.47E-04	1.00
1.4	0.00	1.47E-04	1.00
1.5	0.00	1.47E-04	1.00
1.6	0.00	1.47E-04	1.00
1.7	0.00	1.47E-04	1.00
1.8	0.00	1.47E-04	1.00
1.9	0.00	1.47E-04	1.00
2.0	0.00	1.47E-04	1.00

Pure dissolution model

Dissolution may occur either because the API has appreciable solubility in the wash solvent or in mixtures of the wash solvent and the crystallization solvent. Such phenomenon of a maximum solubility being reached in mixtures of solvents is relatively common. For example, in this work two different cases where simulated which showing binary solubility plot of the test compound with a maximum: paracetamol in isopropanol-water mixture and paracetamol in isopropanol-acetonitrile mixture. These simulations were compared with simulations of case studies where the binary solubility plot did not show maximum, with the objective of clearly highlight the solid fraction dissolution in the wash curve. Dissolution process can be seen in the wash curve plot observing the solute concentration in liquid phase curve (orange curve). In case of solid phase dissolution, the solute concentration is increasing during the first wash ratio (Figure S16). This solute concentration increment is directly correlated to the raise in solubility due to the crystallisation and wash solvent mixing with a certain solvents ratio range. For instance, the dissolution of paracetamol in isopropanol-water mixture (Figure S16) is happening when the water ratio is between 0 and 0.5. At higher water ratio, the dissolution mechanism stops, since the solubility of paracetamol starts to decrease, and the dilution of the solute due to the addiction of wash solvent can be observed with a gentle and gradual solute concentration reduction.

The data used to plot these graphs are reported in the ESI.

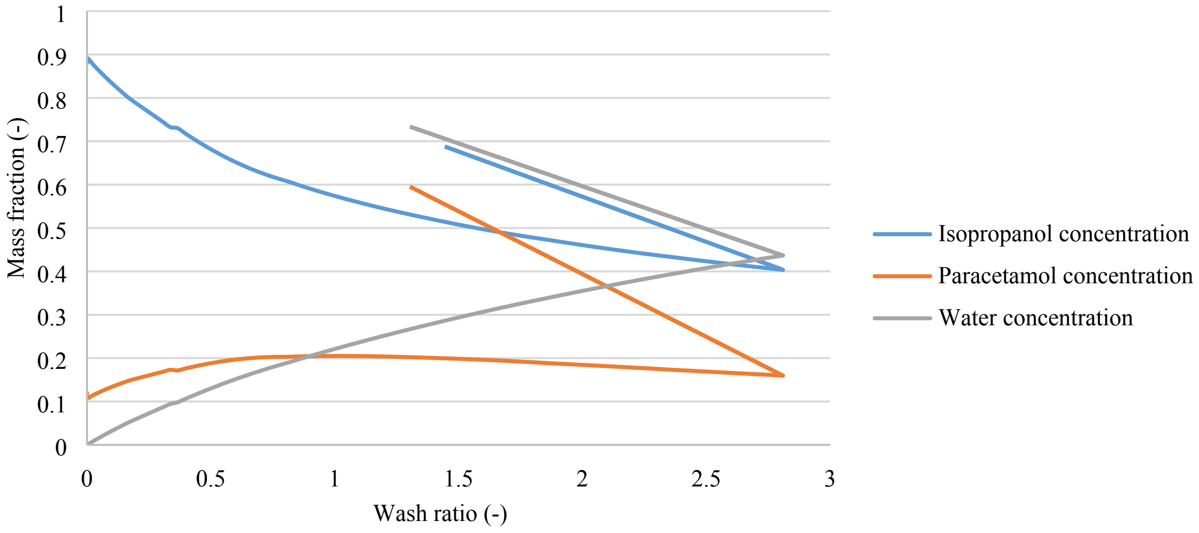

Figure S16 Wash curve obtained from pure dissolution model. Paracetamol was selected as test compound. Isopropanol was chosen as the mother liquor solvent. The wash solvent used was water. The wash curve is showing the evolution of isopropanol, wash solvent and solute paracetamol at different wash ratio.

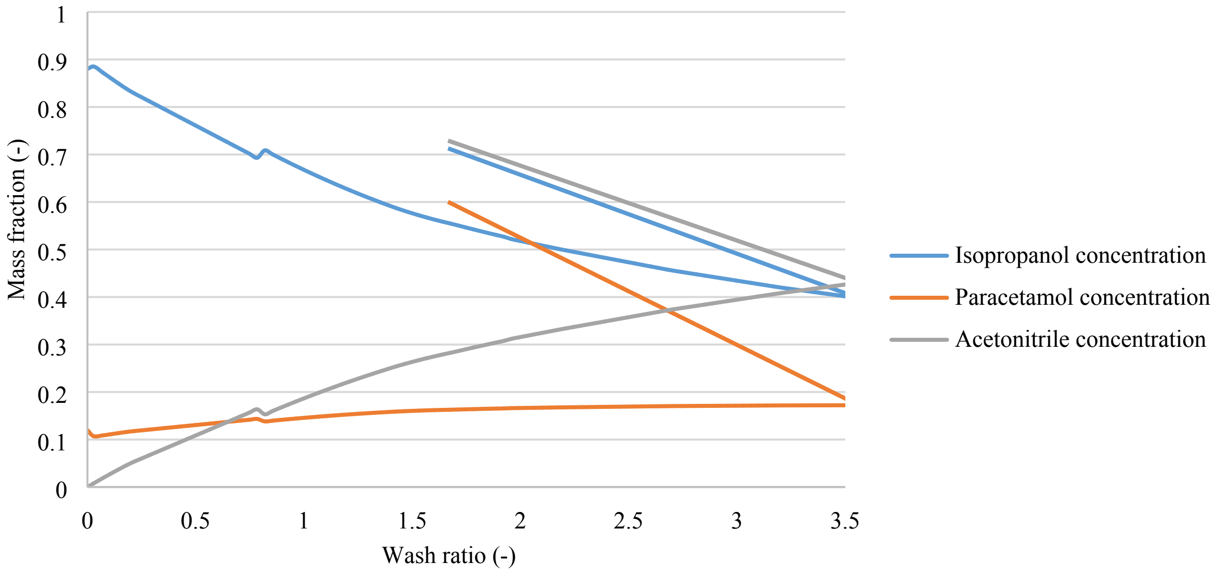

Figure S17 Wash curve obtained from pure dissolution model. Paracetamol was selected as test compound. Isopropanol was chosen as the mother liquor solvent. The wash solvent used was acetonitrile. The wash curve is showing the evolution of isopropanol, wash solvent and solute paracetamol at different wash ratio.

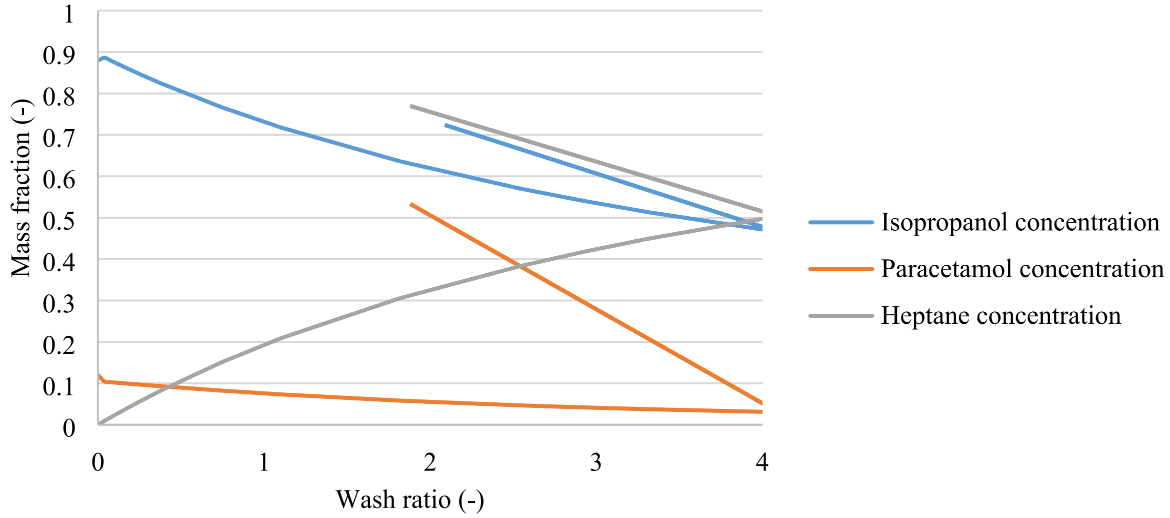

Figure S18 Wash curve obtained from pure dissolution model. Paracetamol was selected as test compound. Isopropanol was chosen as the mother liquor solvent. The wash solvent used was heptane. The wash curve is showing the evolution of isopropanol, wash solvent and solute paracetamol at different wash ratio.

Comparing paracetamol isopropanol-water (Figure S16) with isopropanol-acetonitrile (Figure S17) wash curves and the binary solubility plots it is evident how the shape of the maximum can influence the dissolution process. For Isopropanol-water case the order of solubility increment is from water content 0 to the maximum is 1.86 times, showing a steeper increment respect isopropanol-acetonitrile case (1.66 times). Even more drastic is the solubility drop from the maximum to pure wash solvent for isopropanol-water case (13.8 times) respect the isopropanol-acetonitrile case (1.33 times). The solubility drop different is clearly observed in the trend of the solute curve at the end of the dissolution process: for isopropanol-water (Figure S16) case an evident drop of solute concentration is seen, while for isopropanol-acetonitrile case (Figure S17) a quasi-stable solute concentration was simulated. The rate of variation of solute from the end of the dissolution event for the isopropanol-water and isopropanol-acetonitrile cases is reported in Table S13, showing 1-order of solute concentration change in isopropanol-water case.

Table S13 Rate of solute concentration variation after the dissolution process calculated for the paracetamol isopropanol-water and paracetamol isopropanol-acetonitrile case to show the rate of concentration change.

	**Isopropanol-water case[1]**		**Isopropanol-acetonitrile case**
Rate of solute concentration from 1 and 1.5 wash ratio (-)	0.01379	Rate of solute concentration from 2 and 2.5 wash ratio (-)	0.00585
Rate of solute concentration from 1.5 and 2 wash ratio (-)	0.02864	Rate of solute concentration from 2.5 and 3 wash ratio (-)	0.00360
Rate of solute concentration from 2 and 2.5 wash ratio (-)	0.03122	Rate of solute concentration from 3 and 3.5 wash ratio (-)	0.00174
Rate of solute concentration from 1 to 2.5 (-)	0.02455	Rate of solute concentration from 2 to 3.5 (-)	0.00373

Since the isopropanol-heptane binary solubility plot does not show a maximum, the wash curve just shows dilution of the solute phase. Since the rate of solubility drop from pure isopropanol to pure heptane is quite steep, the rate of solute concentration observed is approximately of 0.01309 in the range of 2 and 3.5 wash ratio (comparable with the isopropanol-water case).

This model can be used to simulate dissolution processes, also named as solvent swap, but the simulation needs to be extended for longer process time.

The simulated wash curve for paracetamol isopropanol-dodecane, and mefenamic acid 2-butanol-heptane cases are reported below.

[1] The rate of solute concentration for isopropanol-water case are absolute values, since the solute concentration is decreasing during time, while in isopropanol-acetonitrile case a slight solute concentration increment is observed. The rate of solute concentration was calculated as the difference of solute concentration higher wash ratio - solute concentration at lower wash ratio, everything divided by the difference in wash ratio.

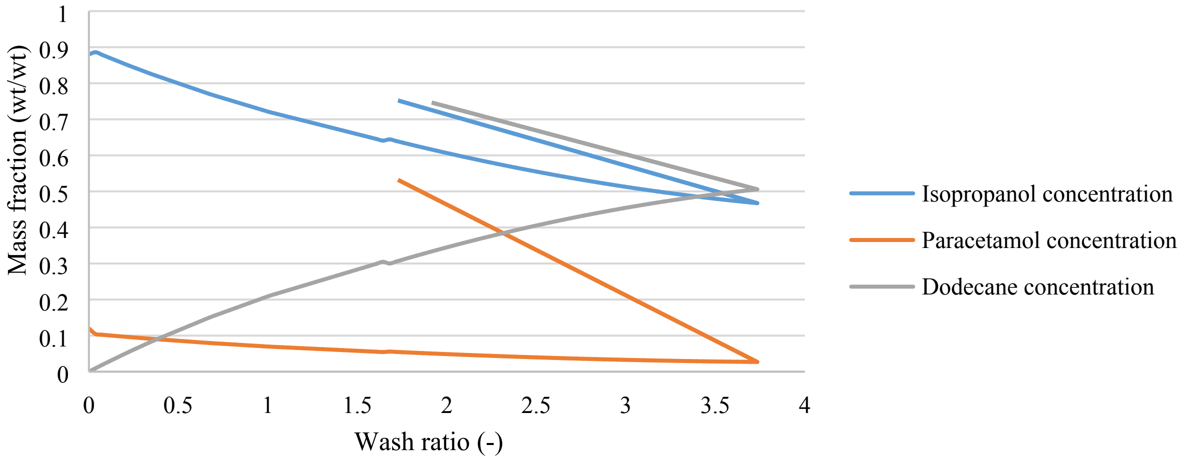

Figure S19 Mass fraction concentration of the different species at different wash ratio simulated with pure dissolution model for the paracetamol case, where dodecane was selected as wash solvent.

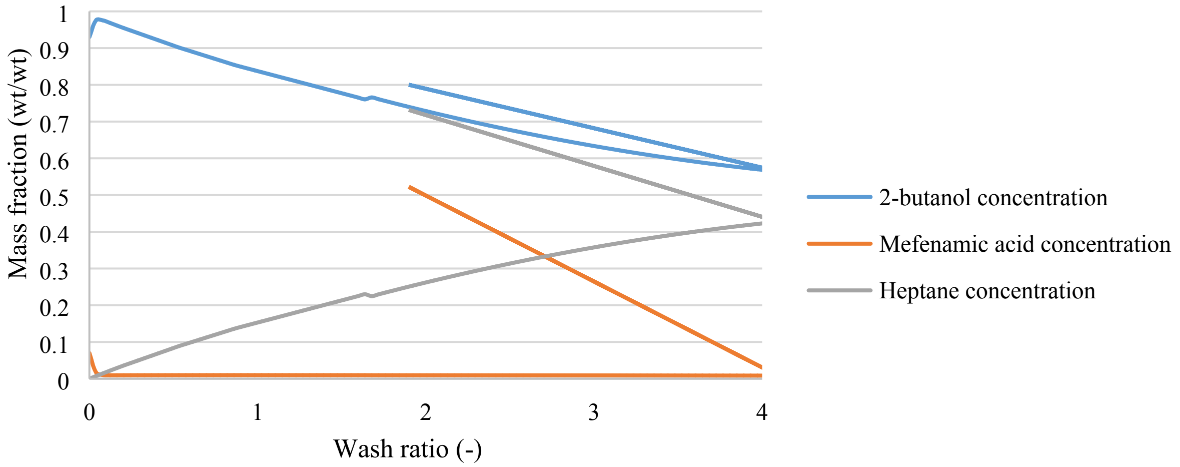

Figure S20 Mass fraction concentration of the different species at different wash ratio simulated with pure dissolution model for the mefenamic acid case.

Dilution with dissolution results

In model 2b dilution and dissolution mechanism are uniform throughout the liquid phase volume across the cake. Since the solubility of the test compound is changing uniformly throughout the entire cake volume, the final particle size distribution simulated is the average value for all the particles forming the cake. Dissolution mechanism is observed only in case the test compound solubility binary plot shows a maximum (increase of the solubility in the mixed solvents respect the pure crystallisation solvent). Therefore, dissolution mechanism is observed in paracetamol

isopropanol-water (Figure S21) and isopropanol-acetonitrile cases. For paracetamol isopropanol-heptane, isopropanol-dodecane, and for mefenamic acid case (Figure S22) no dissolution is occurring. The dissolution process, if occurring, is then followed by dilution of the solute phase, since the solid phase is diluted for the addition of the wash solvent.

In Figure S21a and Figure S22a, the wash curve obtained from dilution with dissolution model for paracetamol cases where water and dodecane as wash solvents are reported, while in Figure S21c and Figure S22c the paracetamol solid phase mass evolution during washing is reported.

These Figures shows the effect of filtration stopped to breakthrough respect dryland cases. In case filtration was stopped to breakthrough (green lines), a drastic drop in crystallisation solvent and solute is observed in the first part of the wash curves, due to the lower content of liquid phase in the washing feed stream. For cases where the solvent mixture shows a solubility maximum (Figure S21) the solute concentration drop is slightly delayed (before 1 Wr) due to the initial mass fraction raise (solid phase dissolution), respect cases where the solubility binary mixture does not show maxima (before 0.5 Wr), Figure S22. To highlight the capability of model 2b to simulate risk and extent of solid fraction dissolution, the crystal mass evolution during washing was simulated, clearly proofing and quantify the extent of cake dissolution (Figure S21c and Figure S22c).

As also observed in model 1b, in case no outflow is observe (green and blue lines), simple dilution is simulated, while in case the inlet and the outlet flow are matching further crystallisation and solute concentration reduction is seen due to the removal of filtrate.

From the solute phase concentration evolution graphs (Figure S21b and Figure S22b), the simulations show that in case of simple dilution with dissolution washing, without considering diffusion mechanism, washing is more effective if filtration is stopped to breakthrough, especially

in case solid fraction dissolution occurs. For these cases (Figure S21c green line), the presence of reduce amount of mother liquor reduce the initial solid dissolution, respect cases where the washed cake was filtered to dryland.

Hypothetically, since model 2b is capable to simulate dissolution and deposition, from the graphs reporting the crystal mass evolution during washing, risk of deposition can be also observed. However, from Figure S21c and Figure S22c, the overall solid mass gain is negligible, and therefore particle deposition cannot be observed.

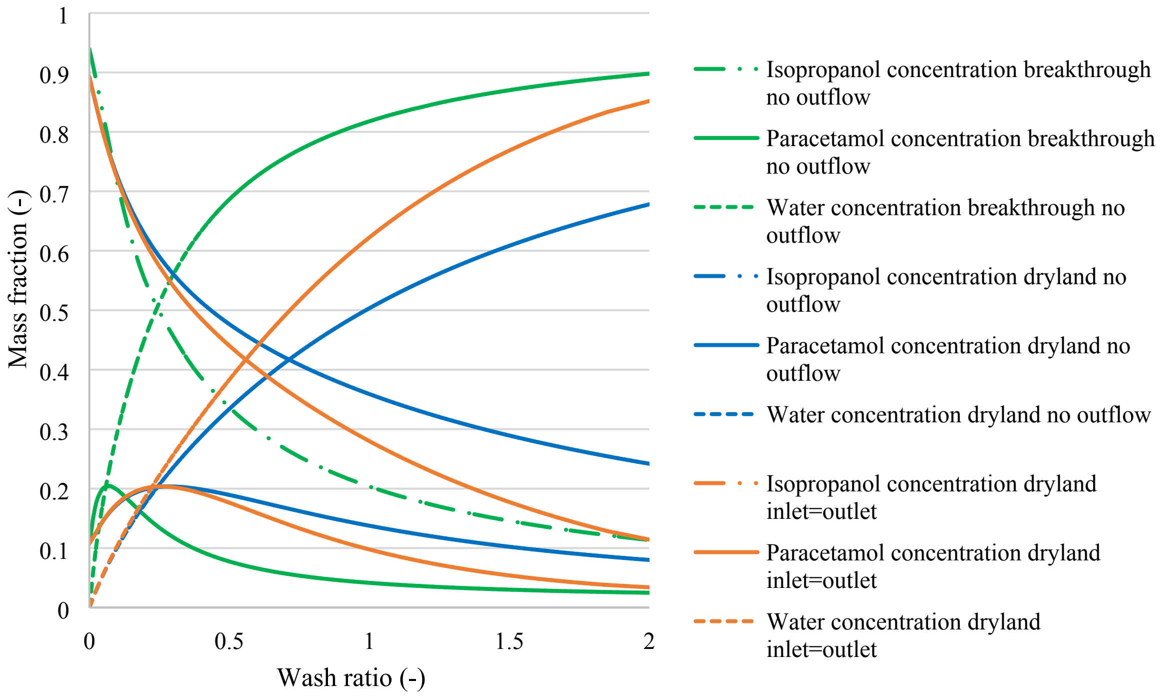

a

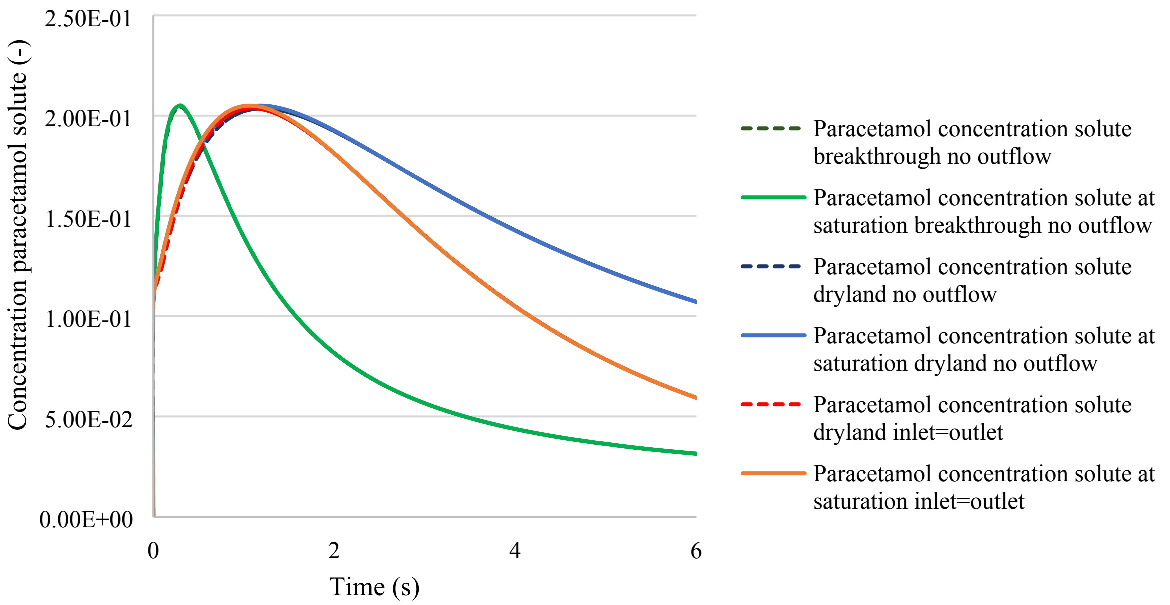

b

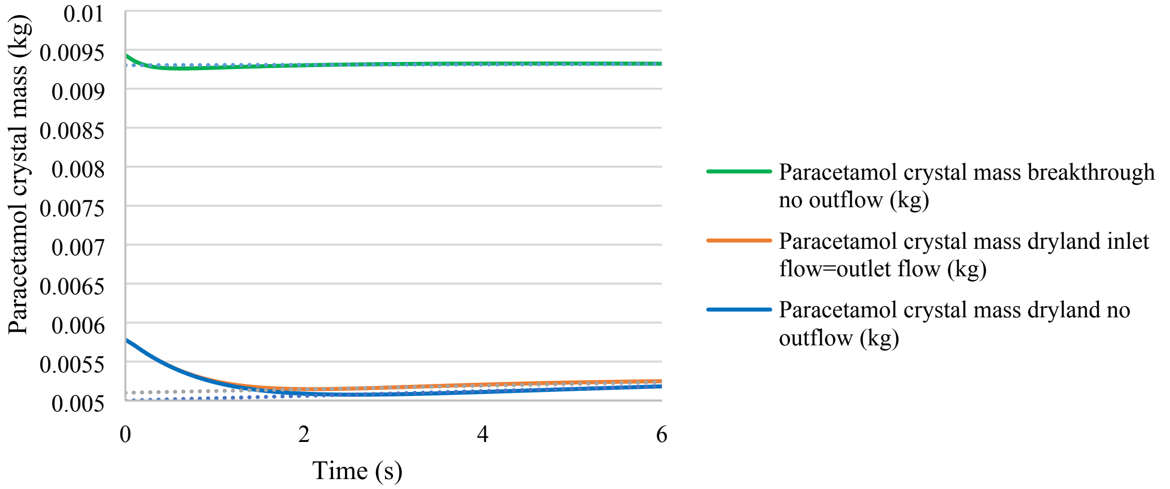

c

Figure S21 a) Wash curve obtained from dilution with dissolution model. Paracetamol was selected as test compound; isopropanol was chosen as the mother liquor solvent. The wash solvent used was water. The wash curve is showing the evolution of isopropanol, wash solvent and solute paracetamol at different wash ratio. b) Solute concentration during the simulated washing process and respectively simulated solute concentration at saturation. c) Paracetamol solid phase mass evolution during washing.

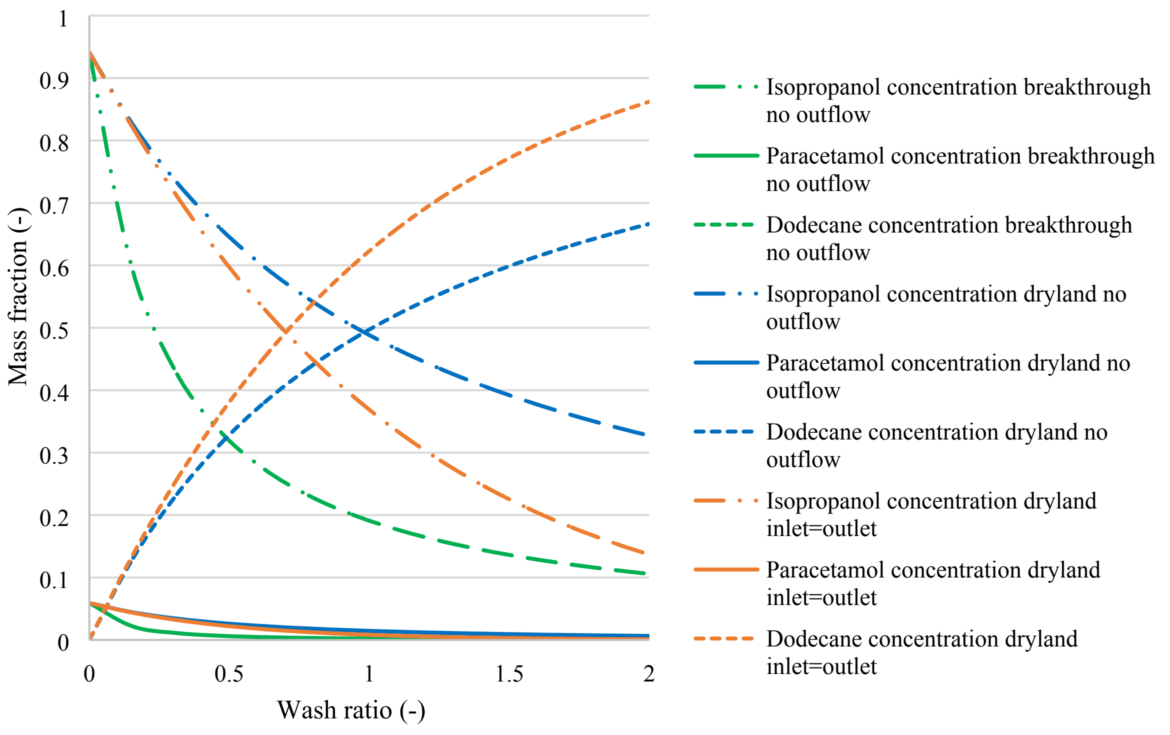
Mass fraction (-)
Wash ratio (-)
1
0.9
0.8
0.7
0.6
0.5
0.4
0.3
0.2
0.1
0
0
0.5
1
1.5
2
Isopropanol concentration breakthrough no outflow
Paracetamol concentration breakthrough no outflow
Dodecane concentration breakthrough no outflow
Isopropanol concentration dryland no outflow
Paracetamol concentration dryland no outflow
Dodecane concentration dryland no outflow
Isopropanol concentration dryland inlet=outlet
Paracetamol concentration dryland inlet=outlet
Dodecane concentration dryland inlet=outlet

a

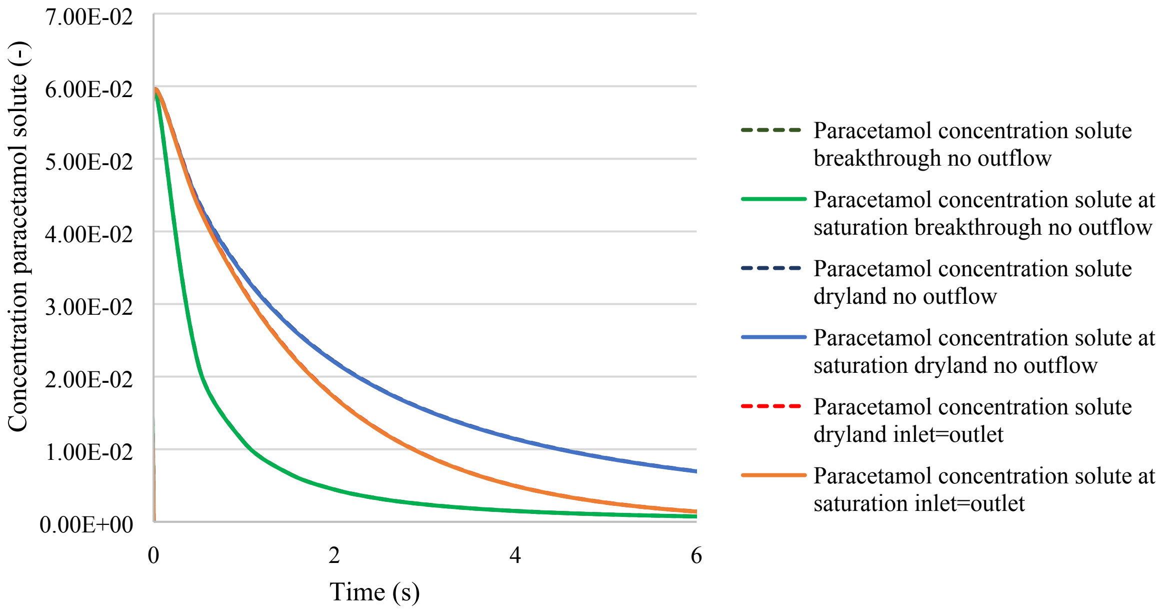
Concentration paracetamol solute (-)
Time (s)
7.00E-02
6.00E-02
5.00E-02
4.00E-02
3.00E-02
2.00E-02
1.00E-02
0.00E+00
0
2
4
6
Paracetamol concentration solute breakthrough no outflow
Paracetamol concentration solute at saturation breakthrough no outflow
Paracetamol concentration solute dryland no outflow
Paracetamol concentration solute at saturation dryland no outflow
Paracetamol concentration solute dryland inlet=outlet
Paracetamol concentration solute at saturation inlet=outlet

b

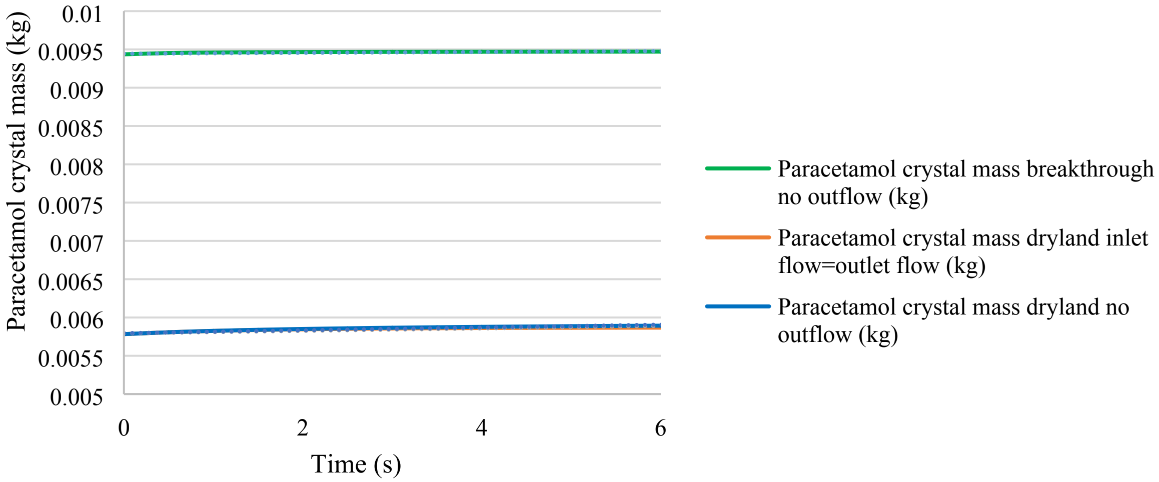

c

Figure S22 a) Wash curve obtained from dilution with dissolution model. Paracetamol was selected as test compound; isopropanol was chosen as the mother liquor solvent. The wash solvent used was dodecane. The wash curve is showing the evolution of isopropanol, wash solvent and solute paracetamol at different wash ratio. b) Solute concentration during the simulated washing process and respectively simulated solute concentration at saturation. c) Paracetamol solid phase mass evolution during washing.

To validate the assumption of model 2b, where it is assumed that the system is in equilibrium through the washing process, in Figure S21b and Figure S22b the evolution of the solute concentration during washing is compared with the solubility evolution. The solute concentration during washing (dotted line) and the solute concentration at saturation condition (continuous line) are perfectly overlapping proofing model 2b assumption for all the cases simulated.

The simulated wash curve for paracetamol isopropanol-heptane, isopropanol-acetonitrile, and mefenamic acid 2-butanol-heptane cases are reported below.

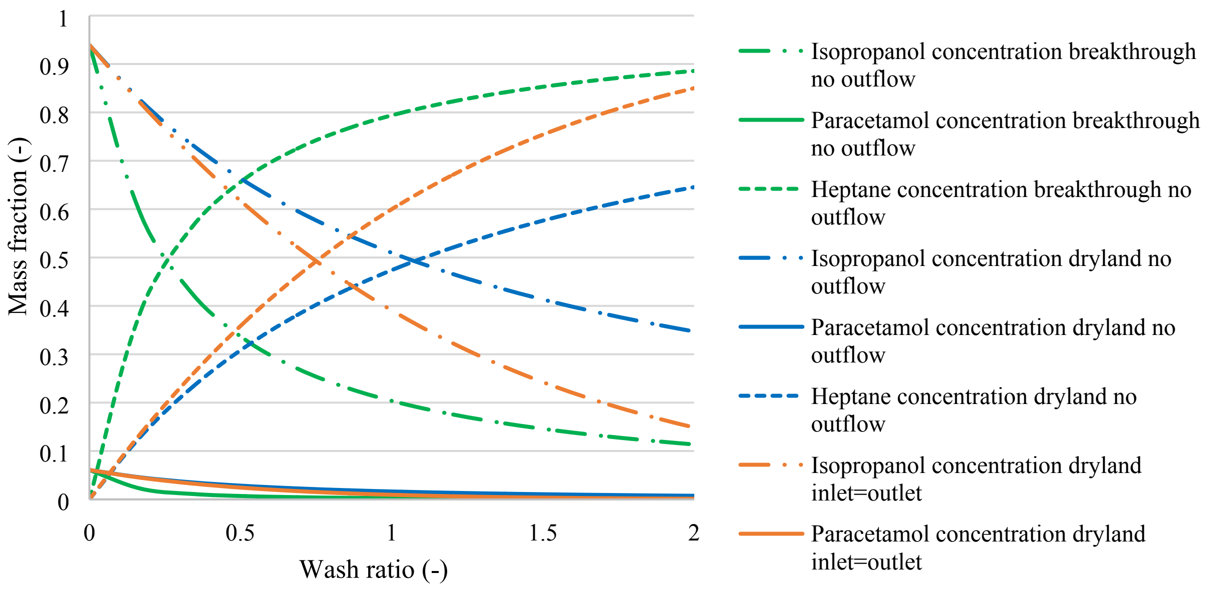
1
0.9
0.8
0.7
0.6
0.5
0.4
0.3
0.2
0.1
0
Mass fraction (-)
0
0.5
1
1.5
2
Wash ratio (-)
Isopropanol concentration breakthrough no outflow
Paracetamol concentration breakthrough no outflow
Heptane concentration breakthrough no outflow
Isopropanol concentration dryland no outflow
Paracetamol concentration dryland no outflow
Heptane concentration dryland no outflow
Isopropanol concentration dryland inlet=outlet
Paracetamol concentration dryland inlet=outlet

a

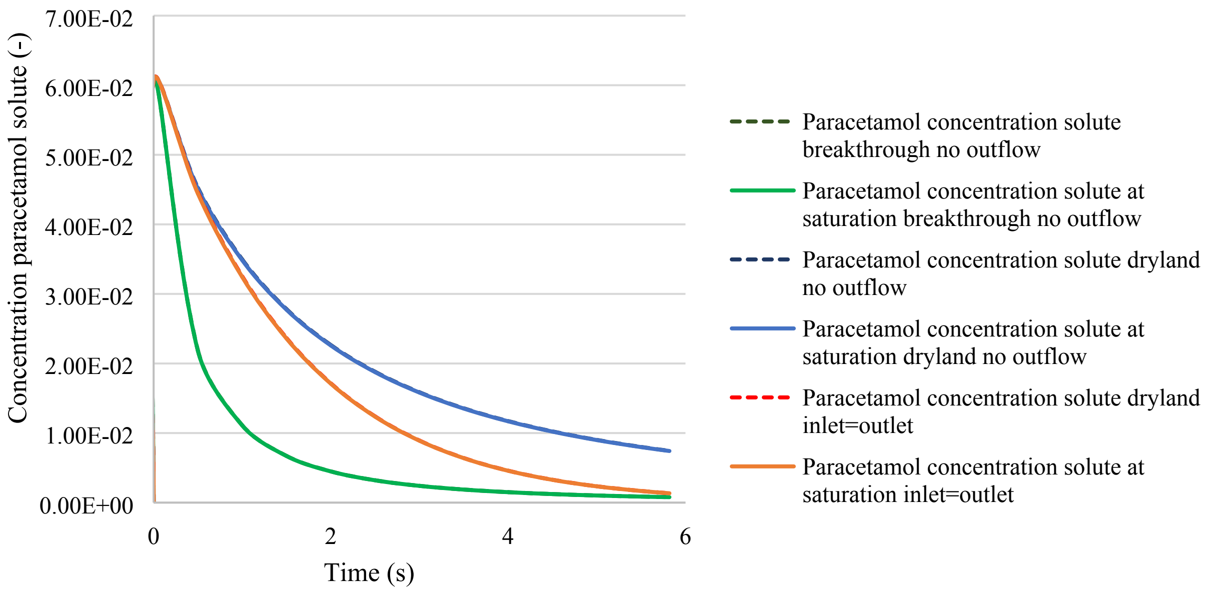
7.00E-02
6.00E-02
5.00E-02
4.00E-02
3.00E-02
2.00E-02
1.00E-02
0.00E+00
Concentration paracetamol solute (-)
0
2
4
6
Time (s)
Paracetamol concentration solute breakthrough no outflow
Paracetamol concentration solute at saturation breakthrough no outflow
Paracetamol concentration solute dryland no outflow
Paracetamol concentration solute at saturation dryland no outflow
Paracetamol concentration solute dryland inlet=outlet
Paracetamol concentration solute at saturation inlet=outlet

b

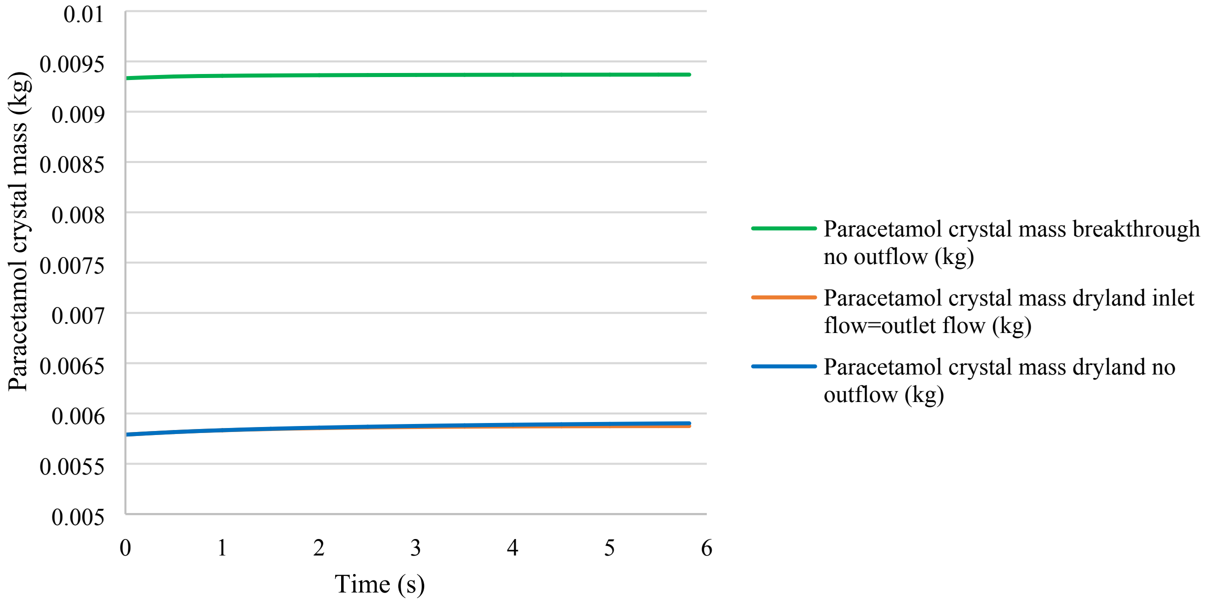

c

Figure S23 a) Mass fraction concentration of the different species at different wash ratio simulated with model 2b for the paracetamol case, where heptane was selected as wash solvent. b) Paracetamol solute concentration during washing for simulated paracetamol case with heptane as wash solvent. c) Paracetamol solid phase mass evolution during washing for the simulated paracetamol experiment with heptane as wash solvent.

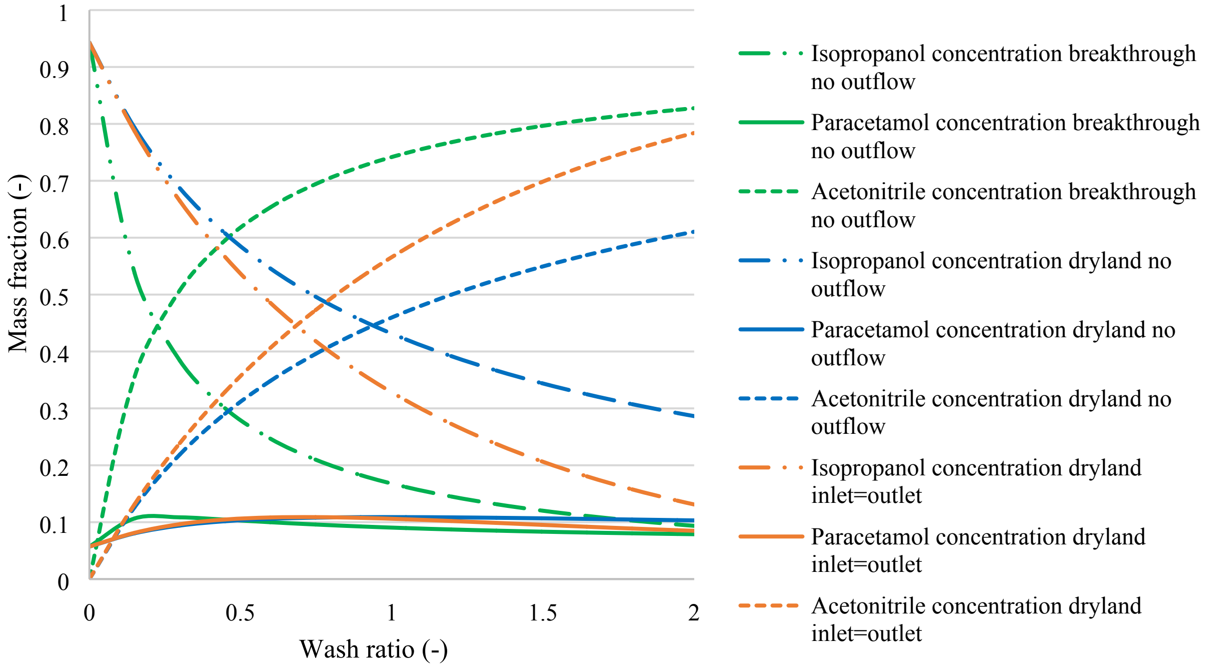

a

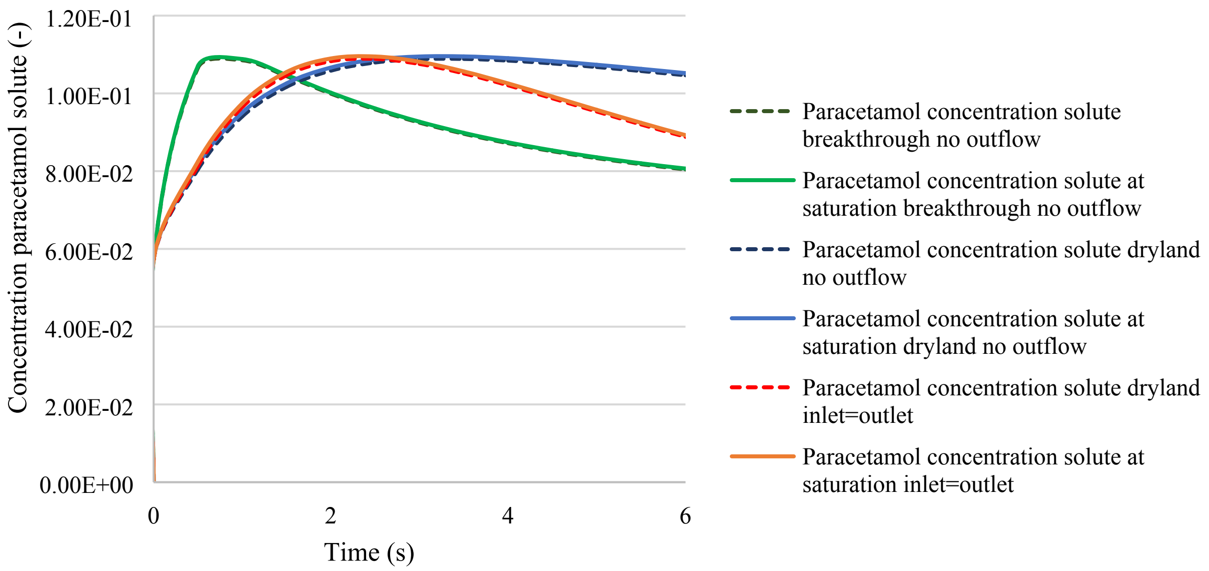

b

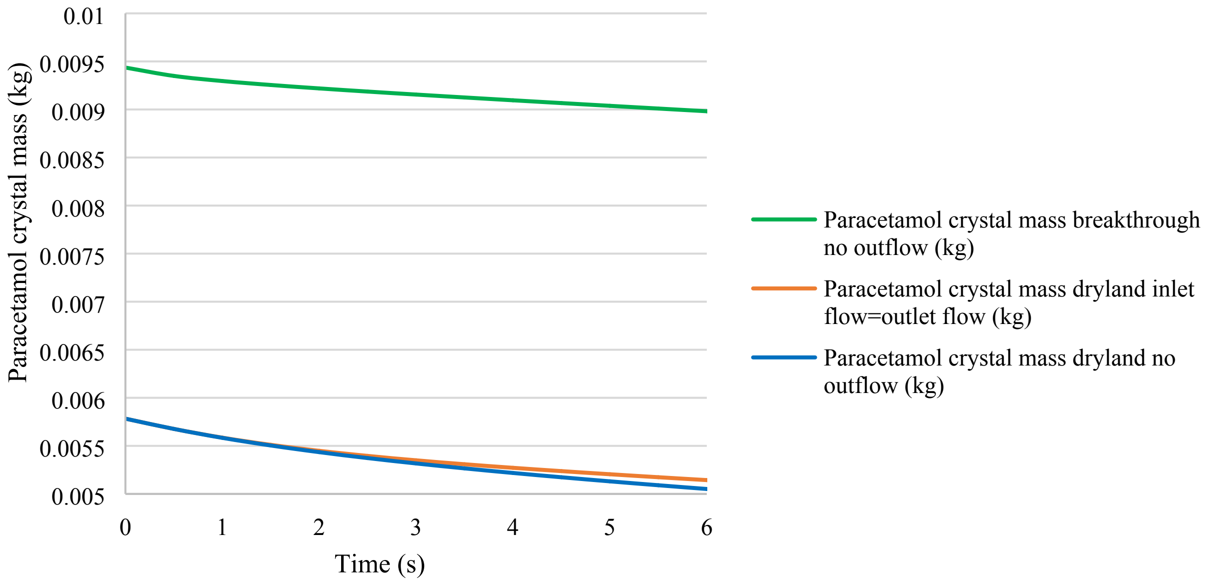

c

Figure S24 a) Mass fraction concentration of the different species at different wash ratio simulated with model 2b for the paracetamol case, where acetonitrile was selected as wash solvent. b) Paracetamol solute concentration during washing for simulated paracetamol case with acetonitrile as wash solvent. c) Paracetamol solid phase mass evolution during washing for the simulated paracetamol experiment with acetonitrile as wash solvent.

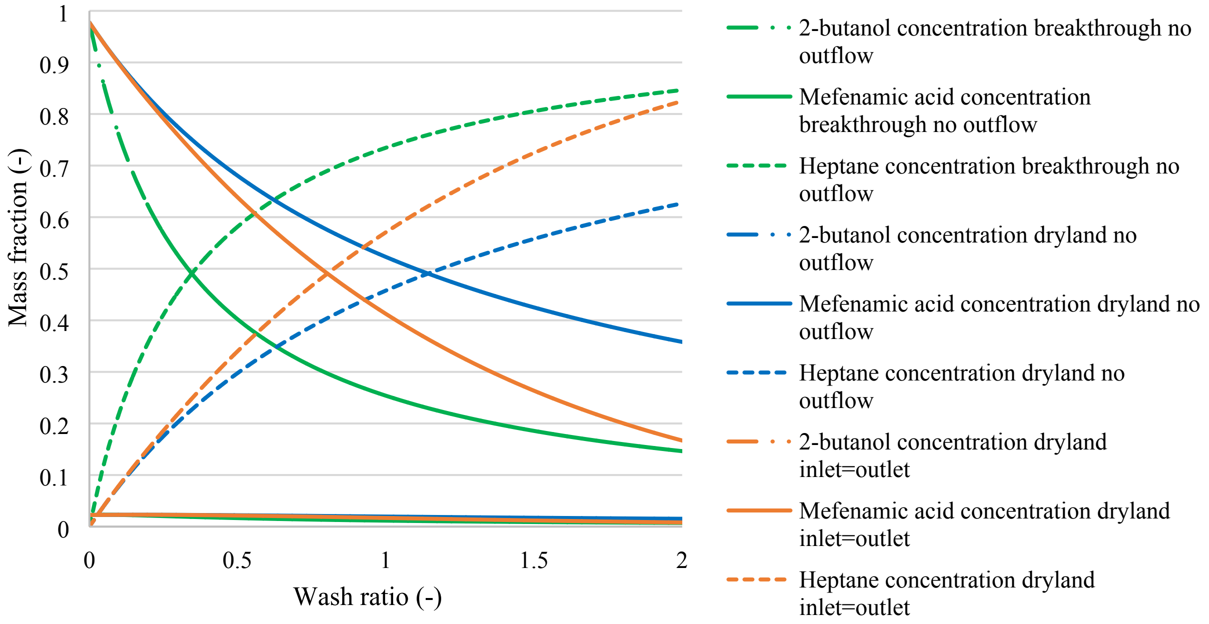
Mass fraction (-)
Wash ratio (-)
1
0.9
0.8
0.7
0.6
0.5
0.4
0.3
0.2
0.1
0
0
0.5
1
1.5
2
2-butanol concentration breakthrough no outflow
Mefenamic acid concentration breakthrough no outflow
Heptane concentration breakthrough no outflow
2-butanol concentration dryland no outflow
Mefenamic acid concentration dryland no outflow
Heptane concentration dryland no outflow
2-butanol concentration dryland inlet=outlet
Mefenamic acid concentration dryland inlet=outlet
Heptane concentration dryland inlet=outlet

a

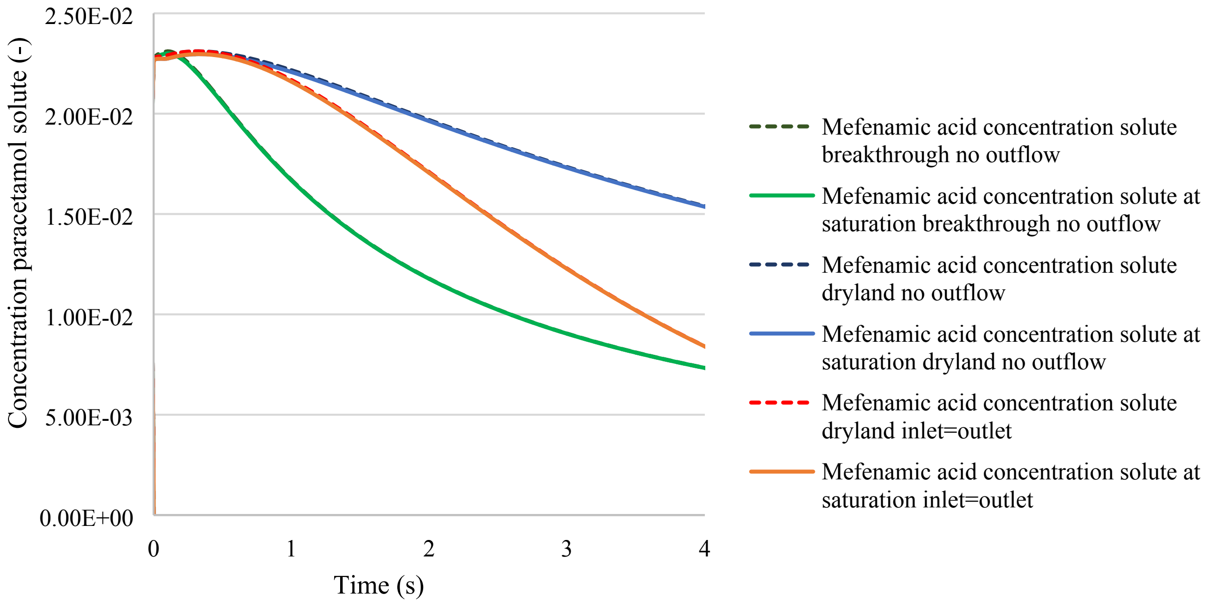
Concentration paracetamol solute (-)
Time (s)
2.50E-02
2.00E-02
1.50E-02
1.00E-02
5.00E-03
0.00E+00
0
1
2
3
4
Mefenamic acid concentration solute breakthrough no outflow
Mefenamic acid concentration solute at saturation breakthrough no outflow
Mefenamic acid concentration solute dryland no outflow
Mefenamic acid concentration solute at saturation dryland no outflow
Mefenamic acid concentration solute dryland inlet=outlet
Mefenamic acid concentration solute at saturation inlet=outlet

b

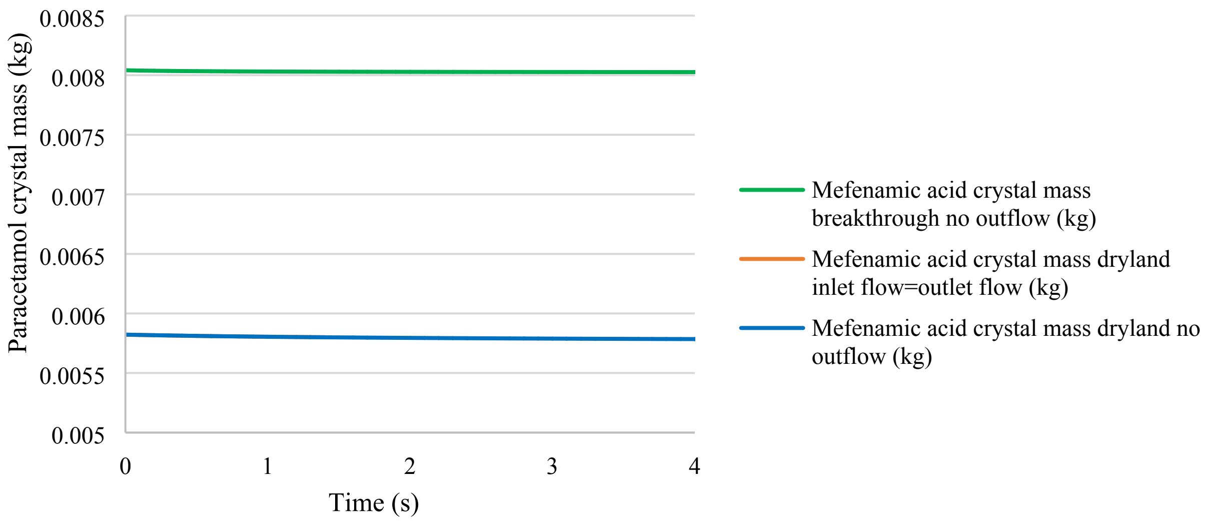

c

Figure S25 a) Mass fraction concentration of the different species at different wash ratio simulated with model 2b for the mefenamic acid case. b) Mefenamic acid solute concentration during washing for simulated paracetamol case with heptane as wash solvent. c) Mefenamic acid solid phase mass evolution during washing for the simulated paracetamol experiment with heptane as wash solvent.

Gradient diffusion and dispersion with dissolution results

Model 2c describes diffusion-dilution with dissolution washing, where solvent composition is not uniformly distributed in the entire cake, but the liquid inside the cake is divided into 10 layers of solvent varying composition where in each layer with instant mixing of the two liquid phases. Layer 1 is the layer of liquid phase near to the surface of the cake, while layer 10 is the layer near the filter media. As reported in section 3.2.6., at the beginning of the washing layer 1 is made of pure wash solvent, while layer 10 is made of pure mother liquor, then with washing progression the liquid composition of each layer is changing following the binary plot solubility curve. In case the liquid mixture shows a binary plot characterised with solute concentration maximum, solid phase dissolution will take place. As reported in Figure S26, paracetamol-acetonitrile is a typical case where solid phase dissolution is observed in the first layers of the cake (near to the surface of the cake). In Figure S26 we can observed the simulated wash curve (Figure S26a). From this graph,

it is observable the two different regimes of washing, constant and falling rate, as also observed in model 1c. The simulated wash curve also shows that during constant rate period no dissolution is occurring, since during this constant rate mainly displacement washing mechanism is taking place. During displacement washing plug flow mother liquor ejection is done only in the main channels of the cake. Instead, during the falling rate period the slow washing mechanism are taking place, dilution, diffusion and dissolution. Indeed, it can be observed from Figure S26a that dissolution is occurring only during the first part of the falling rate period, where the dilution and diffusion of the wash solvent in the trapped mother liquor left in the side channels are occurring. During this part of the washing the evolution of liquid composition is moving from pure mother liquor to pure wash solvent, passing from the solubility maxima that cause dissolution of extra material from the surface of the particles forming the cake (increase of mass fraction of solute, orange line). Figure S26b clearly shows the impact of particle dissolution by plotting the variation of the mean quantile particle size at 50% and variation of cake porosity of the 10 different layers which are forming the cake. For the paracetamol cases here reported the raw material mean size corresponded to 77μm, while the initial cake porosity corresponded to 0.44. From this graph it can be infer that the dissolution effect is mainly occurring in the first layers of the cake, as seen from the increase in cake porosity. Since the quantity of paracetamol dissolved in the liquid phase is now increased due to solid phase dissolution, a higher quantity of this material can deposit on the surface of the crystals promoting agglomeration by forming solid bridges between particles. This is in agreement with the data reported in Figure S26b, where in the first layers of the cake the mean particle size is doubling from 77μm to 137μm. The extent of solute dissolved in the first layers of the cake (1 and 2) gradually decrease, expecting that the excess of solute is used in the first layers of the cake to form particles solid bridges. Moving beyond the first two superficial layers of the cake, the

amount of solute to use for generate solid bridges is limited, causing that in the last layer of the cake, near the filter media, the size of particles is comparable with the size of the raw material. However, since model 2c does not consider any agglomeration or deposition model, the aforementioned hypothesis related to the particle size enlargement are just dictated by past experimental evidences. The particle size trend simulated with model 2c is in good agreement with experimental evidences reported by Ottoboni et al. (2018) [58], where it was experimentally observed that during isolation process agglomeration is occurring on the surface and in the bulk of the cake and in proximity of the filter media cake is behaving as free flowing particle system.

In Figure S26c the solute concentration during the simulated washing process and the solute concentration at saturation are reported. This graph shows that the solute in the liquid phase is kept overall in saturated condition during the entire process; a slightly under-saturation is seen during the last stages of the washing, causing risk of dilution of solid phase.

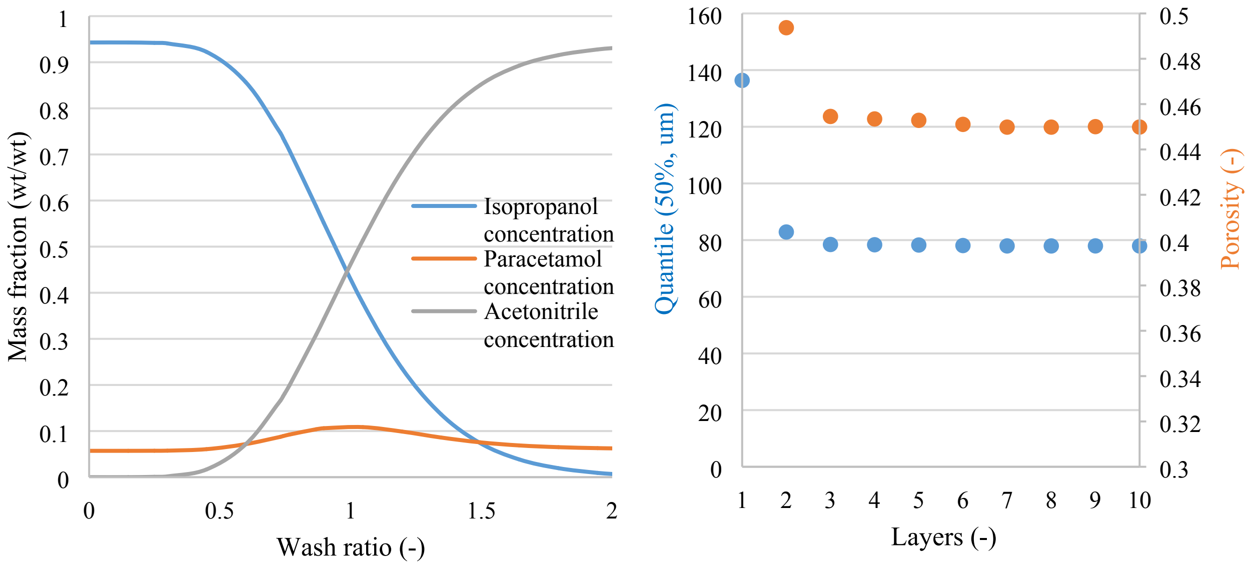

a

b

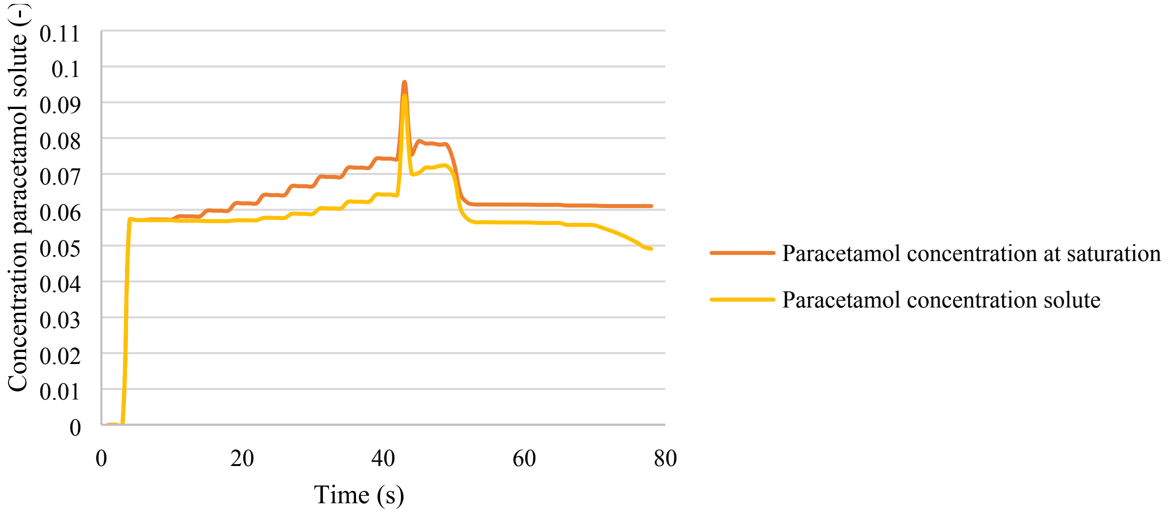

c

Figure S26 a) Wash curve obtained from gradient diffusion and dispersion with dissolution model. Paracetamol was selected as test compound; isopropanol was chosen as the mother liquor solvent. The wash solvent used was acetonitrile. The wash curve is showing the evolution of isopropanol, wash solvent and solute paracetamol at different wash ratio. b) Mean quantile particle size at 50% and variation of cake porosity of the 10 different layers which are forming the cake. c) Solute concentration during the simulated washing process and respectively simulated solute concentration at saturation for the simulation of paracetamol case where acetonitrile was chosen as wash solvent.

To validate model 2c also with a case study where the mixture solvent solubility does not present a maximum, mefenamic acid case was simulated. For the mefenamic acid case, the crystallization solvent selected was 2-butanol and the wash solvent was heptane. As seen in Figure S27a, the wash curve of the solute does not present any dissolution during the first part of the falling rate curve, proofing the capability of this model to discriminate the risk of dissolution during washing. This is confirmed also in Figure S27b, where the mean particle size and cake porosity is overall comparable with the raw material particle size and cake porosity (respectively 93µm and 0.3916). Figure S27c reports the solute concentration during the simulated washing process and solute concentration. This graph shows that the solute in the liquid phase is kept overall in saturated condition during the entire process with a slightly super-saturation seen during the last stages of the washing.

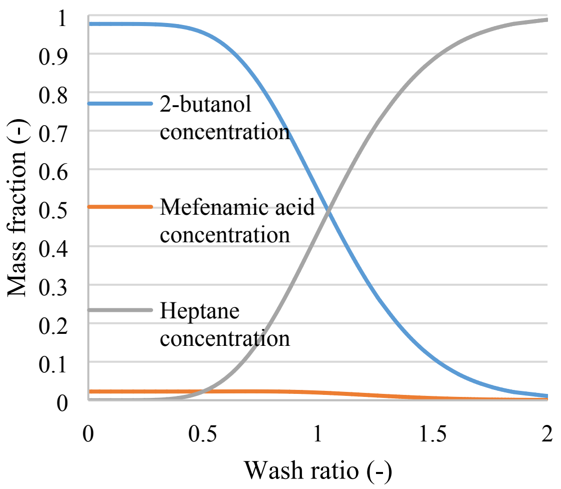

a

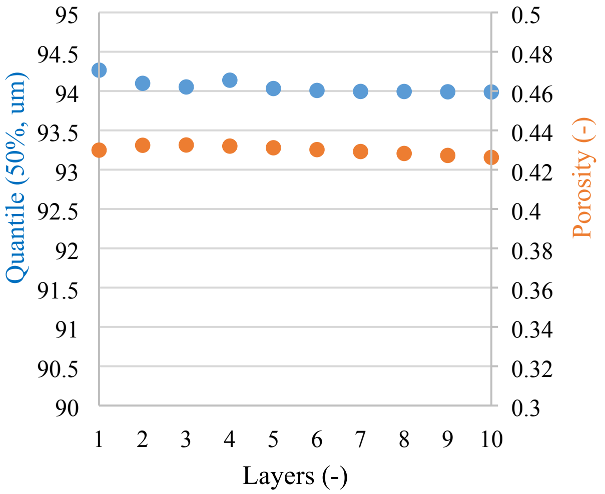

b

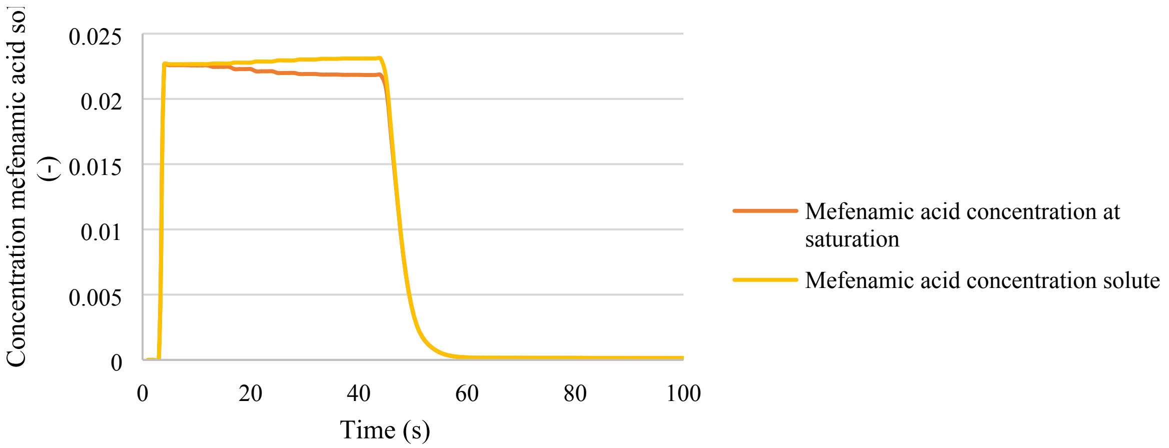

c

Figure S27 a) Wash curve obtained from gradient diffusion and dispersion with dissolution model. Mefenamic acid was selected as test compound; 2-butanol was chosen as the mother liquor solvent. The wash solvent used was heptane. The wash curve is showing the evolution of 2-butanol, wash solvent and solute mefenamic acid at different wash ratio. b) mean quantile particle size at 50% and variation of cake porosity of the 10 different layers which are forming the cake. c) Solute concentration during the simulated washing process and respectively simulated solute concentration at saturation for the simulation of mefenamic acid case where heptane was chosen as wash solvent.

The simulated wash curve for paracetamol isopropanol-water, isopropanol-heptane, and isopropanol-dodecane cases are reported below.

The data used to plot these graphs are reported in the ESI.

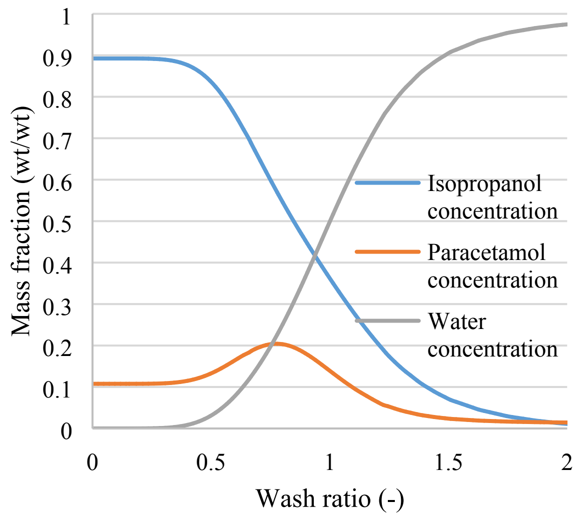

Mass fraction (wt/wt)
1
0.9
0.8
0.7
0.6
0.5
0.4
0.3
0.2
0.1
0
0
0.5
1
1.5
2
Wash ratio (-)
Isopropanol concentration
Paracetamol concentration
Water concentration

a

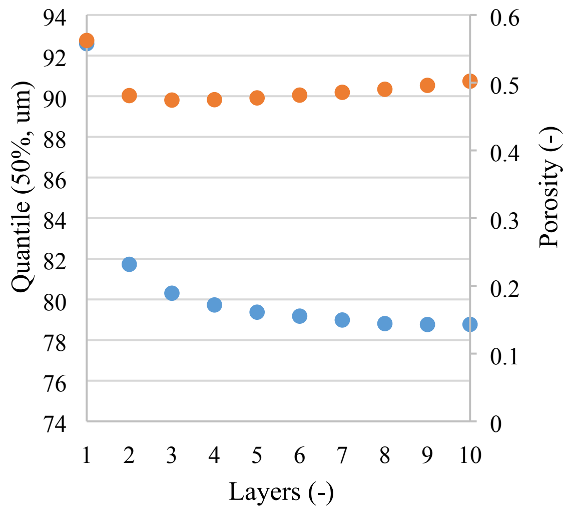

Quantile (50%, um)
94
92
90
88
86
84
82
80
78
76
74
Porosity (-)
0.6
0.5
0.4
0.3
0.2
0.1
0
1
2
3
4
5
6
7
8
9
10
Layers (-)

b

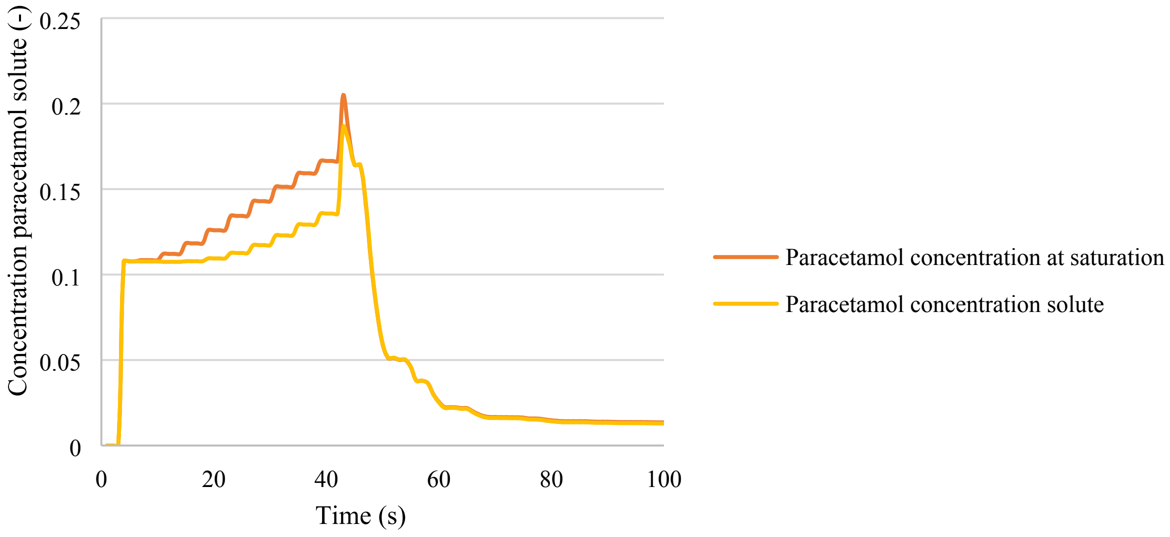

Concentration paracetamol solute (-)
0.25
0.2
0.15
0.1
0.05
0
0
20
40
60
80
100
Time (s)
Paracetamol concentration at saturation
Paracetamol concentration solute

c

Figure S28 a) Mass fraction concentration of the different species at different wash ratio simulated with model 2c for the paracetamol case, where water was selected as wash solvent. b) Mean particle size and porosity evolution during washing for the simulated paracetamol washing strategy where water was chosen as wash solvent. The raw material mean size corresponded to 77µm, while the initial cake porosity corresponded to 0.44. c) Solute concentration during the simulated washing process and respectively simulated solute concentration at saturation for the simulation of paracetamol case where water was chosen as wash solvent. This graph shows that the solute in the liquid phase is kept overall in saturated condition during the entire process (a slightly desaturation is seen during the last stages of the washing, causing risk of particle dissolution).

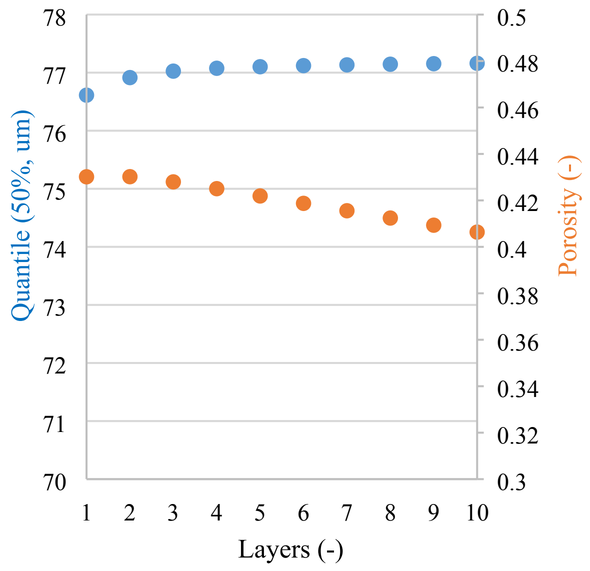

a

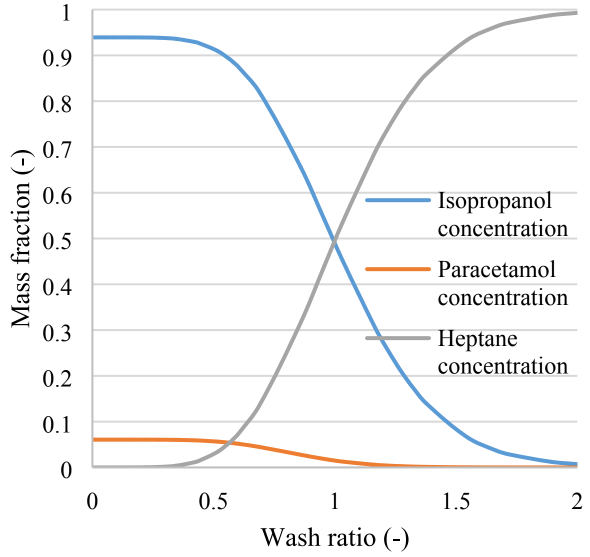

Mass fraction (-)
Wash ratio (-)
Isopropanol concentration
Paracetamol concentration
Heptane concentration

b

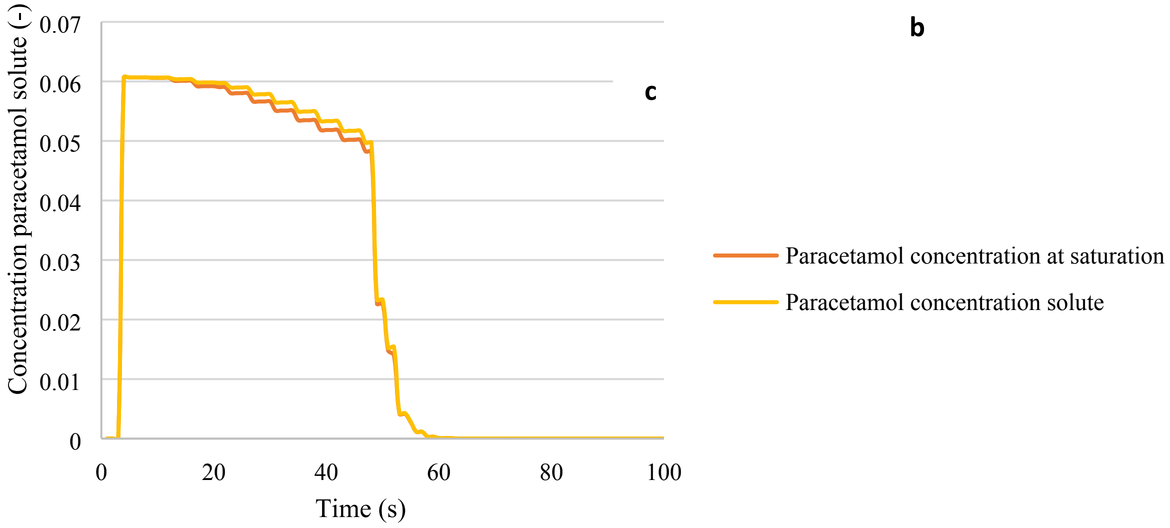

Concentration paracetamol solute (-)
Time (s)
Paracetamol concentration at saturation
Paracetamol concentration solute

c

Figure S29 a) Mass fraction concentration of the different species at different wash ratio simulated with model 2c for the paracetamol case, where heptane was selected as wash solvent. b) Mean particle size and porosity evolution during washing for the simulated paracetamol washing strategy where heptane was chosen as wash solvent. The raw material mean size corresponded to 77µm, while the initial cake porosity corresponded to 0.44. c) Solute concentration during the simulated washing process and respectively simulated solute concentration at saturation for the simulation of paracetamol case where heptane was chosen as wash solvent. This graph shows that the solute in the liquid phase is kept overall in saturated condition during the entire process (a slightly super-saturation is seen during the last stages of the washing, causing risk of deposition of solute).

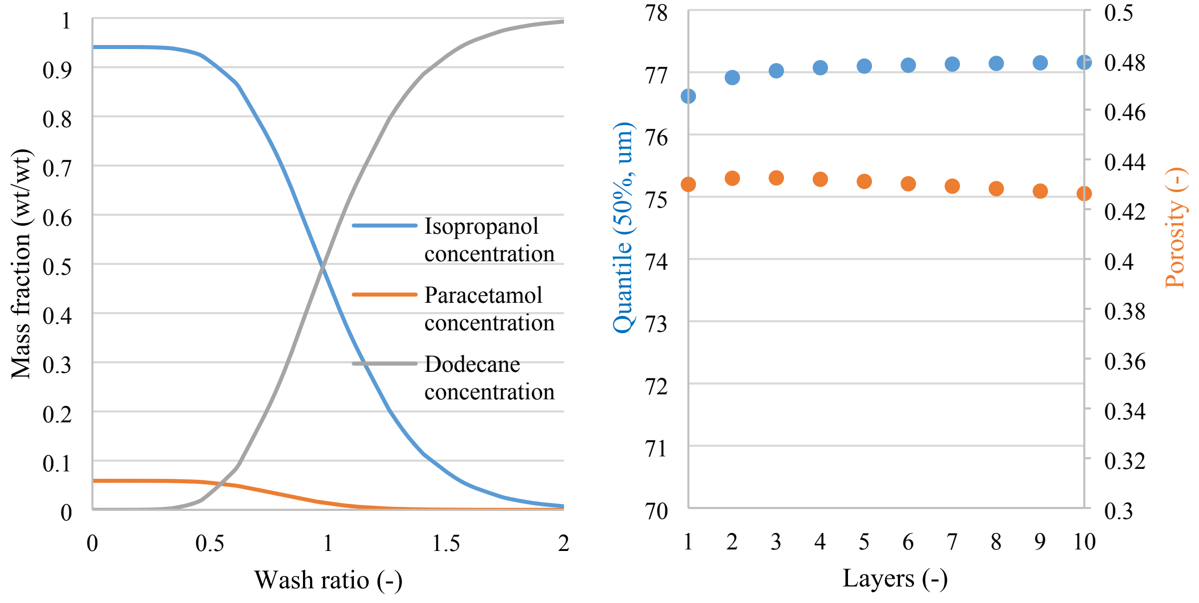

a **b**

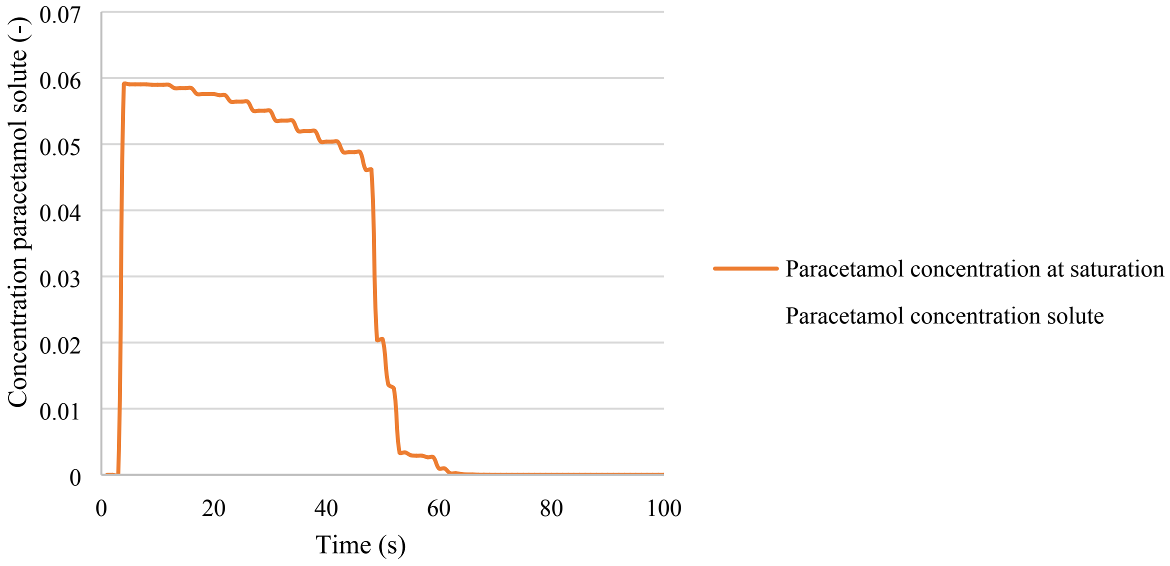

c

Figure S30 a) Mass fraction concentration of the different species at different wash ratio simulated with model 2c for the paracetamol case, where dodecane was selected as wash solvent. b) Mean particle size and porosity evolution during washing for the simulated paracetamol washing strategy where dodecane was chosen as wash solvent. The raw material mean size corresponded to 77µm, while the initial cake porosity corresponded to 0.44. c) Solute concentration during the simulated washing process and respectively simulated solute concentration at saturation for the simulation of paracetamol case where dodecane was chosen as wash solvent. This graph shows that the solute in the liquid phase is kept overall in saturated condition during the entire process (a slightly supersaturation is seen during the last stages of the washing, causing risk of deposition of solute).

Model comparisons

Table S14 Concentration of crystallization solvent (CS), solute, and wash solvent (WS), at 1 wash ratio and 2 wash ratio, and mean particle size simulated for the different models developed.

Model	Concentration CS at 1 Wr	Concentration CS at 2 Wr	Concentration solute at 1 Wr	Concentration solute at 2 Wr	Concentration WS at 1 Wr	Concentration WS at 2 Wr	D50 at 2Wr
**1a: pure displacement model**							
Paracetamol-water	0.8800	0.1200	0	0	0	1	77
Paracetamol-heptane	0.8800	0.1200	0	0	0	1	77
Paracetamol-dodecane	0.8800	0.1200	0	0	0	1	77
Paracetamol-acetonitrile	0.8800	0.1200	0	0	0	1	77
Mefenamic acid-heptane	0.9300	0.0700	0	0	0	1	93
**1b: pure dilution model**							

Paracetamol-water B no outflow	0.0958	0.0512	0.0116	0.0062	0.8926	0.9459	77
Paracetamol-water D no outflow	0.3986	0.2614	0.0481	0.0315	0.5533	0.7070	77
Paracetamol-water D inlet=outlet flow	0.2660	0.0833	0.0321	0.0101	0.7019	0.9067	77
Paracetamol-heptane B no outflow	0.1357	0.0752	0.0088	0.0048	0.8555	0.9199	77
Paracetamol-heptane D no outflow	0.5095	0.3477	0.0329	0.0225	0.4576	0.6298	77
Paracetamol-heptane D inlet=outlet flow	0.8163	0.1719	0.0527	0.0111	0.5794	0.8170	77
Paracetamol-dodecane B no outflow	0.1212	0.0692	0.0076	0.0043	0.8712	0.9264	77
Paracetamol-dodecane D no outflow	0.4644	0.3299	0.0291	0.0207	0.5064	0.6494	77
Paracetamol-dodecane D inlet=outlet flow	0.3418	0.1471	0.0214	0.0092	0.6367	0.8436	77
Paracetamol-acetonitrile B no outflow	0.1196	0.0676	0.0072	0.0041	0.8732	0.9283	77
Paracetamol-acetonitrile D no outflow	0.4714	0.3239	0.0286	0.0196	0.5000	0.6565	77
Paracetamol-acetonitrile D inlet=outlet flow	0.3375	0.1341	0.0204	0.0081	0.6420	0.8577	77
Mefenamic acid-heptane B no outflow	0.1470	0.0835	0.0034	0.0019	0.8496	0.9146	93
Mefenamic acid-heptane D no outflow	0.4454	0.3049	0.0103	0.0070	0.5443	0.6881	93
Mefenamic acid-heptane D	0.2931	0.0921	0.0068	0.0021	0.7002	0.9057	93

inlet=outlet flow							
**1c: dilution-dispersion model**							
Paracetamol-water PF	0.2869	0.0095	0.0346	0.0015	0.6785	0.9893	77
Paracetamol-water CSTR	0.3755	0.013	0.0453	0.0016	0.5792	0.9852	77
Paracetamol-heptane PF	0.4910	0.0095	0.0317	6.16E-04	0.4772	0.9898	77
Paracetamol-heptane CSTR	0.4449	0.0090	0.0287	5.82E-04	0.5264	0.9904	77
Paracetamol-dodecane PF	0.3563	0.0071	0.0224	4.4E-04	0.6213	0.9925	77
Paracetamol-dodecane CSTR	0.3537	0.0082	0.0222	5.15E-04	0.6241	0.9913	77
Paracetamol-acetonitrile PF	0.4136	0.0215	0.0251	0.0013	0.5613	0.9772	77
Paracetamol-acetonitrile CSTR	0.4064	0.0143	0.0246	8.68E-04	0.5690	0.9848	77
Mefenamic acid-heptane PF	0.1624	0.0005	0.0037	1.23E-05	0.8339	0.9995	93
Mefenamic acid-heptane CSTR	0.1380	2.87E-04	0.0032	6.64E-06	0.8588	0.9997	93
**2a**							
Paracetamol-water	0.8800	0	0.1200	0.0100	0	0.99	77
Paracetamol-heptane	0.8800	0	0.1200	5.73E-06	0	1	77
Paracetamol-dodecane	0.8800	0	0.1200	3.49E-06	0	1	73
Paracetamol-acetonitrile	0.8800	0	0.1200	0.1300	0	0.87	77
Mefenamic acid-heptane	0.9900	0	0.0090	3.18E-05	0	1	93
**2b**							
Paracetamol-water B no outflow	0.1391	0.0772	0.0411	0.0249	0.8199	0.8979	78
Paracetamol-water D no outflow	0.3566	0.2418	0.1364	0.0800	0.5070	0.6781	83
Paracetamol-water D inlet=outlet flow	0.2764	0.1142	0.0960	0.0340	0.6276	0.8518	83
Paracetamol-heptane B no outflow	0.1987	0.1136	0.0024	7.86E-04	0.7989	0.8856	77.11

Paracetamol-heptane D no outflow	0.5024	0.3471	0.0159	0.0074	0.4817	0.6455	76.41
Paracetamol-heptane D inlet=outlet flow	0.3794	0.1486	0.0089	0.0013	0.6117	0.8500	76.62
Paracetamol-dodecane B no outflow	0.1772	0.1058	0.0019	6.63E-04	0.8210	0.8936	77.12
Paracetamol-dodecane D no outflow	0.4661	0.3272	0.0132	0.0064	0.5207	0.6664	76.41
Paracetamol-dodecane D inlet=outlet flow	0.3353	0.1367	0.0067	0.0011	0.6579	0.8621	76.62
Paracetamol-acetonitrile B no outflow	0.1617	0.0936	0.0896	0.0789	0.7486	0.8276	80.14
Paracetamol-acetonitrile D no outflow	0.4212	0.2864	0.1089	0.1031	0.4699	0.6105	85.83
Paracetamol-acetonitrile D inlet=outlet flow	0.3139	0.1311	0.1051	0.0849	0.5809	0.7840	84.61
Mefenamic acid-heptane B no outflow	0.1891	0.1078	0.0090	0.0055	0.8019	0.8867	94.05
Mefenamic acid-heptane D no outflow	0.4305	0.2831	0.0173	0.0127	0.5522	0.7043	94.32
Mefenamic acid-heptane D inlet=outlet flow	0.2718	0.0760	0.0123	0.0040	0.7159	0.9197	94.23
**2c**							
Paracetamol-water	0.3502	0.0110	0.1330	0.01439	0.5168	0.9746	80.82
Paracetamol-heptane	0.4528	0.0071	0.0128	3.88E-06	0.5344	0.9929	77.04
Paracetamol-dodecane	0.3522	0.072	0.0075	3.56E-06	0.6393	0.9928	77.04
Paracetamol-acetonitrile	0.3773	0.0069	0.1085	0.0625	0.5142	0.9306	84.42
Mefenamic acid-heptane	0.1395	1.80E-04	0.0069	1.58E-04	0.8536	0.9997	94.06
